
JOURNAL INFORMATION
==============================
NLM Title Abbreviation: J Orthop Traumatol
Journal ID: J Orthop Traumatol
NLM Journal ID: 101090931

Journal of Orthopaedics and Traumatology : Official Journal of the Italian Society of Orthopaedics and Traumatology

ISSN: 1590-9921
EISSN: 1590-9999
Publisher: Springer

ARTICLE INFORMATION
==============================
PMCID: PMC4417795
DOI: 10.1007/s10195-013-0264-9
Article ID: 264
Article version: 1
Subjects: Events

In-Depth Oral Presentations and Oral Communications

Electronic publication date: 2013 Sep 18
Print publication date: 2013 Oct
Volume: 14
Issue: Suppl 1
First page: 83
Last page: 121
Copyright: © The Author(s) 2013
License: Open AccessThis article is distributed under the terms of the Creative Commons Attribution 2.0 International License (https://creativecommons.org/licenses/by/2.0), which permits unrestricted use, distribution, and reproduction in any medium, provided the original work is properly cited.
License URL: https://creativecommons.org/licenses/by/2.0/

Keywords: Total Knee Arthroplasty, Total Knee Replacement, Tibial Component, Mechanical Axis, Clubfoot
issue-copyright-statement: © The Author(s) 2013

==============================
J Orthopaed Traumatol (2013) 14 (Suppl 1):S83–S121
DOI 10.1007/s10195-013-0264-9

29 OCTOBER 2013

### In-Depth Oral Presentations and Oral Communications

### IN DEPTH ORAL PRESENTATIONS

### *A14–KNEE*

### Chondral lesions and tissue repair: clinical and radiological outcomes one year after post-operative application of platelet rich fibrin

R. Papalia*[1], F. Franceschi[1], L. Diaz Balzani[1], B. Zampogna[1], N. Maffulli[2], V. Denaro[1]

[1]Università Campus Biomedico (Rome, IT);
[2]Centre for Sports and Exercise Medicine, Barts and The London School of Medicine and Dentistry (London UK)

**Introduction** Cartilage lesions are the most common cause of chronic knee pain. The current treatment options consist in conservative strategies, such as visco-supplementation and platelet-rich plasma injections, and surgical management including debridement of the chondral lesion, bone marrow stimulation with microfractures and osteochondral transplant. In this study we combined arthroscopic microfracturing with platelet concentrates, platelet-rich plasma (PRP) and Vivostat platelet-rich fibrin (PRF).
**Materials and methods** Since September 2011 we conducted a prospective trial on 51 patients divided into 3 groups with clinical and radiological evidence of cartilage lesions. All patients underwent a knee arthroscopy with microfracture. The platelet concentrate was administered intra-operatively to the PRF group and post-operatively to the PRP group, as a cycle of 3 intra-articular injection. Finally, the microfractures group underwent only microfractures (control group). Clinical scores (IKDC, KOOS, VAS) have been administered at 6 and 12 months post-operative and was performed a radiological examination with MRI evaluating lesions of patients according to the Mocart criteria (2006).
**Results** All patients achieved a statistically significant clinical improvement from pre-operative to post-operative time. In particular, patients who were treated with platelet concentrates achieved better clinical results compared to patients treated with microfracture only ($p < 0.0001$). Comparing PRF group and PRP group patients, the first group showed a significant increase compared to the second of the IKDC scores ($p = 0.0349$), KOOS ($p = 0.0003$), VAS ($p = 0.0023$) at 6 months, with loss of significance at 12 months. From a radiological point of view the PRF group obtained better results earlier than the other two groups.
**Discussion** The application of intra-operative platelet concentrate associated with microfractures has led to significant improvement 6 months postoperatively both clinically and radiologically. The rationale is that a greater concentration of platelets and growth factors leads to a greater stimulation of the bone marrow and a earlier formation of repair tissue.
**Conclusions** Comparing Vivostat PRF application with PRP injections a more rapid and less painful recovery is evident. However, the repair tissue does not have the same characteristics of the articular cartilage. Studies of histology and microscopic anatomy are required to assess the quality of this tissue to define its characteristics.

### Novel biomimetic scaffold: pilot clinical study at 5-year follow-up

E. Kon, S. Patella, G. Filardo, A. Di Martino*, F. Perdisa, B. Di Matteo, M.L. Merli, M. Marcacci

Laboratorio di Biomeccanica/NaBi, IOR (Bologna, IT)

**Introduction** The regeneration ability of cartilage is limited and we do not have evidence-based methods for the treatment of cartilage defects in the knee which are often difficult to treat. Numerous approaches have been proposed as innovative solutions for cartilage defects with variable success rates. In particular, for lesions involving both cartilage and subchondral bone, a biomimetic scaffold has been developed to repair osteochondral defect inducing an in situ cartilage and subchondral bone regeneration.
**Materials and methods** An osteochondral scaffold was obtained by enucleating equine collagen type 1 fibrils with hydroxyapatite nanoparticles in 3 different layers with 3 different gradient ratios at physiological conditions. 30 patients (9 females, 21 males, mean age 29.3 years) affected by symptomatic grade III–IV chondral and osteochondral lesions of the knee (ICRS evaluation package) were enrolled and underwent implantation of the scaffold with a press-fit one-step technique. Twenty-five patients were analyzed prospectively at 6, 12, 24, 36, 48 and 60 months using the Cartilage Standard Evaluation Form as proposed by ICRS and high resolution MRI.
**Results** We detected a statistically significant clinical improvement in all clinical scores ($p < 0.05$) with respect to pre-operative assessment. In particular IKDC score showed a statistically significant improvement with positive trend since the 24 months follow-up and then the results were stable over time until the final evaluation. A significant increase was registered at 12 months follow-up also for Tegner score with good results confirmed even at 5 years evaluation. These results show a statistically significant improvement ($p < 0.05$) from pre-operative level, even if the final sport activity level is lower than the pre-injury one. MRI evaluation revealed a good integration of the scaffold and a satisfactory filling of the defect.
**Discussion** This minimally invasive one-step surgical approach seems to be an easy and effective procedure. The results registered are very encouraging and this procedure shows a satisfactory outcome even in large osteochondral lesions or complex cases.
**Conclusions** Further randomized studies with longer term follow-up and a higher number of patients are still needed to confirm the efficacy showed by this procedure.

### Osteochondral knee transplants: how and when

A. Ruffilli*, L. Ramponi, P. Capra, C. Pungetti, S. Giannini

Istituto Ortopedico Rizzoli (Bologna, IT)

**Introduction** Post-traumatic osteoarthritis of the knee in young and active patients represents a challenge. In literature, the monopolar allograft is well known, instead more doubts exist about massive, bipolar and total knee allograft (FTSOA). Aims of this study is to evaluate massive and partial knee allografts as substitutes for knee replacement in young patients with post-traumatic osteoarthritis and to determinate the genetic typing of chondrocytes DNA.
**Materials and methods** Fourteen patients (mean age 40 ± 9 years) affected of post-traumatic knee osteoarthritis were treated with total knee allograft (7 cases) and with a partial one (7 cases). Patients were evaluated clinically (IKDC score) and radiographically (X-rays, CT and MRI), pre-operatively and at regular follow-ups. Biopsies were obtained during overhaul and analyzed according to histological, immunohistochemical and genetic typing of DNA microsatellites.
**Results** The IKDC score in patients treated with total allograft grown up from 33.7 ± 4 points pre-operatively to 40.4 ± 13 at 12 months of follow-up. Six patients failed at 19.5 ± 3.9 months of follow-up. In the youngest patient of the group, which had a previous arthrodesis, a satisfactory result was obtained (65 points at the IKDC score at 24 months and at 48 months). In case of graft failures, a severe laxity joint has developed with joint effusion: then it was necessary to perform total joint replacement. The presence of giver's DNA suggest a host recolonization of the allograft. Patients treated with partial osteochondral transplantation achieved an improvement in the IKDC score from 32.7 ± 11.2 to 79.7 ± 14.1 points at the latest follow-up of 31.3 ± 13.8 months. Allograft consolidation and ROM recovery were satisfactory at 4–6 months.
**Discussion** BFTOA in the knee failed in most cases, despite the good integration after 6 months. However, the excessive size of the graft and the transplantation of also soft tissues probably caused inflammatory reaction and consequent instability. Partial allograft resulted an efficient treatment for unicompartmental osteoarthritis in young and active patients, suggesting that the few volume of the grafts could be positive prognostic factor for the consolidation.
**Conclusions** Massive knee osteochondral transplantation showed a high failure rate despite excellent setting, good consolidation and integration of host's cell. The partial knee osteochondral allograft proved to be a reliable treatment for unicompartmental osteoarthritis in young adult. However, controlled trials with higher number of patients should be performed and the role of immunological reactions should be analyzed.

### Patellar retention with Oxinium femoral component versus cobalt-chromium: clinical and radiological results at medium-term

F. Matassi*, G. Munz, L. Sirleo, C. Carulli, R. Civinini, M. Innocenti

CTO, AOU Careggi (Florence, IT)

**Introduction** The purpose of the present study is to analyzed the clinical and radiological outcomes of total knee replacement without patellar resurfacing in oxidized zirconium versus cobalt-chromium femoral component.
**Materials and methods** We prospectively analyzed 80 patients, 52 females and 28 males, the mean age was 79.5 years, who had total knee replacement (TKR) between April 2008 and December 2010. The cohort was divided in two groups based on type of implanted component: 40 TKR with Oxinium femoral component and without patellar resurfacing (group 1) and 40 TKR with Cr–Co femoral component without patellar resurfacing (group 2). All patients were evaluated clinically through Knee Score System (KSS) and Patello-Femoral Score (PFS) and radiologically with antero-posterior and latero-lateral projections of the knee and patellar axial view at 45° at 3, 6 months and 1, 2 years after surgery.
**Results** At the final follow-up the average Knee Score was 88.80 (range 80–97) for Oxinium group and 86.75 (range 79–95) for Cr–Co group and mean Functional Score was respectively 96.25 (range 87–100) and 92.65 (range 86–100), without any significant difference. The PFS was slight superior for Oxinium group with 89.60 (range 80–100) range points compared to Cr–Co with 85.20 (range 78–100).
**Discussion** The differences in Knee and Functional Scores between Oxinium and Cr–Co femoral component groups did not reach a statistical significance. Superior PFS was recorded for Oxinium component.
**Conclusions** In case of patellar retention the use of Oxinium femoral component is effective to allow high clinical score.

### *A15–PAEDIATRIC ORTHOPAEDICS*

### The outcome of congenital clubfoot surgical treatment: the role of tibiotalar incongruence in a long term analysis

A. Bernasconi*, A. Casaburi, I. Mercurio, F. Carbone, F. Sadile

A.O. Federico II (Naples, IT)

**Introduction** Clubfoot is the second orthopaedic congenital deformity for frequency. The natural evolution leads to a permanent disability. Where conservative treatment fails, surgical therapy is required. At the end of surgical procedure, after the section of capsule, ligaments, tendons and muscles has allowed to correct manually the deformity, the surgeon may find out a certain degree of tibio-talar incongruence, in which the distal surface of tibia does not perfectly meet the troclea of the talus. The purpose of our study was to assess the role of that incongruence in a long-term outcome (about 20 years) in people affected by clubfoot that underwent surgery in the first 12 months of their life.
**Materials and methods** We visited and interviewed 26 clubfeet by using the VAS-FA and the 4 scales developed by AOFAS. According to the presence or absence of the tibio-talar incongruence and its degree (accurately reported in the operative records), subjects have been divided in two groups: group 1 (tibio-talar incongruence absent or slight) and group 2 (strong and pronounced). To understand if there were marked differences between the two groups, it was performed an analysis of variance (ANOVA) on the results of VAS-FA scale and a Mann–Whitney U test on those of AOFAS scales.
**Results** The ANOVA demonstrated that the mean values of the two groups being compared are statistically different ($p < 0.05$) for 3 of the 4 categories considered by the score. The Mann–Whitney U test showed statistically significant differences between the two groups ($p < 0.05$) in terms of pain and function of the ankle and of the whole foot.
**Discussion** The perfect concurrence of the analysis done on both questionnaires' results does suggest a prospective usefulness of the tibio-talar morphology assessment as a long term prognostic factor.
**Conclusions** Considering clubfeet surgically treated in the first 12 months of life, not affected by other kinds of disease impairing their ability to walk, the long term outcome (20 years) is statistically different according to the presence or absence (and its degree) of a tibio-talar incongruence found out during surgical treatment.

### Evaluation by gross motor function measure of a pilot aquatic exercise program for children with cerebral palsy

L. Labianca*[1], C. Calderaro[1], P. Rota[1], M.C. Vulpiani[1], F. Turturro[1], A. Montanaro[1], F. Mirco[2], A. Ferretti[1]

[1]Azienda Ospedaliera Sant'Andrea, Università "La Sapienza" (Rome, IT);
[2]IRIFOR (Ascoli Piceno, IT)

**Introduction** Cerebral palsy (CP) is the most common physical disability in childhood. Aquatic intervention is one of the most popular supplementary treatments, but there is a lack of aquatic activity programming for this population. In this study are reported the preliminary results of a new rehabilitation program (active induced hydro-stimulating multi-sensorial method, AIHM), developed in water, that tries to get the target using the well-known water properties in rehabilitation and undergoing patients to active exercise that stimulate an improvement in muscular strength.
**Materials and methods** Descriptive measures and characteristics, such as age, gender, body weight, length, type of CP and the expanded and revised Gross Motor Function Classification Scale level (GMFCS) were recorded. The primary outcome measures were the Gross Motor Function Measure 88 (GMFM-88). The GMFM-88 is a standardised 88-item observational instrument developed to measure changes in gross motor function over time. Each participant was taught by one therapist. The therapy was focused and performed individually. Therapy consist in a first phase of about 15 min with set stretching exercises, using water resistance and specific operations performed by the therapist, 30 min of active exercise or active induced exercises (in patients with poor ability to understand instructions) and 10 min of play. Each patient underwent therapy two times a week for 6 months.
**Results** Twenty-five children with CP aged 5–14 years were recruited. All children followed a standard water therapy, have no medical contra-indications, no botulin toxin treatment or surgery in the preceding 3 months and written parental approval. Fourteen children completed the hydrostimulation protocol (HPG), and 11 children completed all measurements but only participated as control group (CG). There was no statistically significant differences between HPG and CG in age (EG: 9.21 years ± 2.45, CG: 9.92 years ± 2.32), weight and height. There was no difference in GMFM score (post-treatment score, PTS) between the groups at the start of the intervention. After the treatment period there was a statistically significant improvement in GMFM in the HPG group.
**Discussion** The present study found significant effects following a 6 months aquatic intervention on the gross motor function of children with CP comparing the new method described and standard hydrokinetic protocol. Future studies with a larger sample size and longer and more intensive interventions are needed.
**Conclusions** Early rehabilitation is crucial for children with severe disabilities. Protocols for water rehabilitation are still not well developed. We describe a new method focused on active multisensorial stimulation.

### Ultrastructural analysis of ligaments and capsules in clubfoot

A. Casaburi*, A. Bernasconi, V. Franzese, T. Busiello, F. Sadile

A.O. Federico II (Naples, IT)

**Introduction** Clubfoot is a deformity characterized by pathologic findings in both osseous and soft tissue. The aim of this study is to evaluate if there are any differences in capsule structure between people affected and not; it is made up on morphologic observation of extracellular matrix and its components (collagen and others).
**Materials and methods** We have histologically analyzed 14 samples from intra-operative excisions of capsules from 10 children, affected by severe clubfoot (4 bilaterally), underwent to achilloplasty and posterior capsulectomy under 6 months of age (cases) and we have compared them with 9 samples from no affected (controls). To obtain tissue sections, all samples were formalin-fixed, dehydrated through alcohol, embedded in a paraffin block and sectioned on a microtome. On glass slides, antigenic sites for immunohistochemistry were exposed, then visualized through immunofluorescence. Three independent observers expressed an opinion on immunopositivity at the microscope, from 0 to 5 (from absence to plenty).
**Results** At the microscope, it was pointed out a certain raise of tenascin and type I collagen and a fair raise of fibronectin in cases compared to controls. There were, instead, no marked differences in vimentin, type III and type IV collagen.
**Discussion** Histological features of extracellular matrix represent, in any case, a crucial knot in the genesis of capsules and ligaments' tightness in clubfoot. Notwithstanding this, in literature there are not many studies about it.
**Conclusions** The presence of a large quantity of tenascin, protein involved in embrionary development but poorly represented after birth, strengthens the hypothesis of its key role in the pathogenesis of clubfoot. The raise of type I collagen and the fair raise of fibronectin, instead, could explain the deep mechanic abnormalities in extracellular matrix, which are as more important as more severe is the affection. There were, instead, no marked differences in vimentin, type III and type IV collagen in cases compared to controls.

### Oblique pelvic osteotomy in bladder exstrophy in neonatal age

M. Giordano*, A. Di Lazzaro, A. Poggiaroni, R.M. Toniolo, F. Falciglia, V. Guzzanti

Ospedale Pediatrico Bambino Gesù (Rome, IT)

**Introduction** Bladder exstrophy is a rare congenital urologic anomaly, associated with skeletal defects of the pelvis and wide pubic diastasis. The goals of orthopaedic intervention is the closure of the bony pelvic ring facilitating the closure of the bladder and abdominal wall and avoiding insidious wall tension.
**Materials and methods** From November 2005 to May 2012, 14 patients (11 males and 3 females) aged from 2 days to 3 and a half months were treated for bladder exstrophy. Early complete primary reconstruction and repair was performed in five patients. In eight cases delayed surgical procedure was adopted. A patient of 6 years and 6 months was treated for recurrence of cloacal exstrophy. Bilateral oblique pelvic osteotomy was performed in conjunction with genitourinary repair according to Grady Mitchell. In neonatal age, a post-operative cast immobilization, maintained for a month, was used to stabilize osteotomy. The patient with recurrent cloacal exstrophy was stabilized by a pelvic external fixator. Patient follow-up ranged from 8 months to 6 years and 8 months. Pubic approximation was measured on antero-posterior basic radiographs before and after surgery and at final follow-up.
**Results** Clinical evaluation showed in all cases a perfect wound healing and consolidation of the osteotomies. There were no signs of infection or bone dysplasia, or tenderness in the osteotomy site. There was no recurrence of exstrophy with dehiscence of the abdominal wall. The hip function and lower-limb patterns appeared normal. In no case any significant leg length discrepancy was observed. A partial recurrence of pubic diastasis was observed in all cases.
**Discussion** Pelvic osteotomy reduces the tension of the abdominal wall during closure of exstrophy with eventual achievement of urinary continence. The surgery should be performed in the first days/weeks of life in conjunction with bladder reconstruction. The partial recurrence of pubic diastasis during growth does not adversely affect either the functional result of the urinary tract or the function of the pelvis and lower limbs.
**Conclusions** Oblique osteotomy is relatively easy in execution and little traumatic for neuro-vascular and muscular structures. It represents an effective support for urological reconstruction, by eliminating abdominal wall tension through pelvic ring closure. It results in good orthopaedic and urological function thus facilitating postoperative management.

### Comparative costs analysis in idiopathic valgus knee treatment: eight plate versus Blount staples

A. Martinelli*[1], L. Marchesini Reggiani[1], M. Cravino[2], A. Bosco[1], O. Donzelli[1]

[1]Istituto Ortopedico Rizzoli (Bologna, IT);
[2]Ospedale Infantile Regina Margherita (Turin, IT)

**Introduction** One of the classical methods of treatment of idiopathic valgus knee is the epiphysiodesis with Blount staples, in recent years a new alternative device has been proposed by Orthofix, a two holes titanium plate-screws construct, called eight-plate. The literature provides encouraging data but many authors complained about the excessive cost of this new device. Our work provides a comparative cost analysis of Blount staples and eight-plate.
**Materials and methods** A retrospective analysis of 48 patients with idiopathic knee valgus was performed, 24 (11 males, 13 females) treated with Blount staples and 24 (15 males, 9 females) treated with 8-plate. For all patients were taken into account: surgical time, days of hospitalization after surgery, number of implanted devices, devices cost, associated procedures. The operating room cost at Istituti Ortopedici Rizzoli is 3.7 Euros/min, the cost of hospitalization is 466 Euros/day. A Blount staple costs 36.4 Euros while an eight-plate construct costs 816.4 Euros.
**Results** The mean time of hospitalization after implant surgery was 3.7 days in Blount staples group and 2.4 days in eight-plate group. Mean surgical time of implantation for Blount staples was 64.7 and 36.7 min for eight-plates. Mean surgical time for staples removal was 52.5, 26.7 min for eight-plates. The mean hospitalization time after hardware removal was 2.8 days for staples and 2.3 days for eight-plates. We had no complications in the eight-plate group. A patient in the staples group underwent a second surgical procedure due to hardware mobilization. The mean cost of treatment with Blount staples resulted to be 4,968.9 Euros, 4,888.8 Euros with 8-plate.
**Discussion** Beyond the biomechanical aspects, which should be further investigated, we think that eight-plates proved to be superior as a device due to its greater simplicity and reproducibility of the surgical technique. Data found in literature also corroborate the low rate of complications we had. New products similar to eight-plate are appearing on the market and this puts forward a desirable reduction of Orthofix's device market price.
**Conclusions** The costs associated with the implantation of the 8-plate are substantially similar to those with Blount staples. The device choice in idiopathic valgus knee treatment should be primarily based on clinical and experiential parameters of the surgeon.

### *A16–HAND AND WRIST*

### Functional surgery of upper limb in tetraplegia

G. Caserta*[1], G. Petrella[1], P. Mariano[1], A. Barbati[2], A. Landi[1]

[1]Chirurgia della Mano (Modena, IT);
[2]Policlinico Universitario "S.S. Annunziata" (Chieti, IT)

**Introduction** The author describes his almost 20 year experience in functional surgery of tetraplegia.
**Materials and methods** The authors provide a description of different types of upper limb functional reconstructions such as elbow extension rebuilt and reactivation of hand grip function, through a case history of 212 patients on 268 surgical treatments.
**Results** It is described the elbow extension restoration by posterior deltoid-to-triceps transfer, through a tendon graft in 78 cases or by biceps to triceps transfer in 3. As for hand grip surgery, he describes the two steps reconstruction techniques, extensor and flexor, and shows modifications and improvements reached over the years.
**Discussion** The author provides a report of the experience, including modifications and results gained, even after 20 years of monitoring from treatment.
**Conclusions** The report describes the multidisciplinary way in which these patients are evaluated and the different evaluation procedures agreed by the multidisciplinary team.

### Treatment algorithm for scaphoid nonunion: clinical and radiographic results at a mean follow-up of five years

A. Bini[1], N. Guindani*[1], N. Migliavacca[2], G. Regazzola[1], G. Pilato[1]

[1]Uninsubria (Varese, IT);
[2]Ospedale Civile (Legnano, IT)

**Introduction** Scaphoid nonunion presents various anatomo-pathologic types, and each has been dealt with by using different strategies over time. However, to date there is still no common agreement regarding treatment. In this study we analyse the clinical and radiographic findings after scaphoid nonunion surgery and describe the process used at our institution, which is based on lesion morphology, resultant deformity, and fragment blood supply.
**Materials and methods** This is a retrospective study with an average follow-up of 5 years (range 1.5–10 years), involving 65 patients (drop out 15.6 %) treated between 2000 and 2010. Patients were grouped depending on the localisation of the nonunion: proximal (n = 8), middle (n = 36) and distal (n = 21) third. In distal and middle third, the fibrous tissue was resected, the bone defect was packed in with cancellous bone if no deformity was present, while corticocancellous bone graft was harvested in presence of dorsiflexion. Fixation was achieved with K-wires or compression screw. In proximal third we used a vascularised bone graft as described by Zaidenberg in case of avascular fragment, otherwise cancellous bone graft; fixation was achieved in both cases with K-wires. Proximal pole resection arthroplasty was performed in presence of small avascular proximal fragment. At the follow-up strength, ROM, pain and functional outcomes with DASH and Mayo Wrist score were recorded. X-ray evaluation concerned the healing rate and radiocarpal morphology.
**Results** At the follow-up scaphoid healing occurred in 87 % (n = 18) of proximal, 86 % of middle (n = 31) and 100 % (n = 8) of distal third. The mean VAS score was 0.7 in proximal, 1.2 in middle and 0.5 in distal third. The average ROM was 118° in proximal, 166° in middle and 130° in distal third. The strength compared with the contralateral side was 80 % in proximal, 86 % in middle and 90 % in distal third. Time to union was 3.7 months for the proximal third and <3 months for middle and distal thirds. Good results were achieved in 97 % according to DASH and Mayo scores.
**Discussion** The goal of treatment scaphoid nonunion is functional recovery, and to reduce the development of wrist osteoarthrosis. Clinical and radiographic results are comparable with those already described in literature. The localisation of the nonunion influenced the outcome: the more proximal, the worse the prognosis is.
**Conclusions** An algorithms process may help select the most appropriate treatment, by applying it to the anatomopathologic patterns of scaphoid nonunion in order to optimise the results.

### SNAC (scaphoid non-union advanced collapse): algorithm of treatment

M. Rampoldi[1], D. Tagliente[2]

[1]Ospedale CTO (Rome, IT);
[2]Università Roma II Policlinico Tor Vergata (Rome, IT)

**Introduction** The natural history of untreated or not healed scaphoid nonunion is a progression to secondary radiocarpal osteoarthritis and carpal collapse (SNAC wrist). Many types of surgical treatment are described, however the treatment of this pathology continues to be problematic.
**Materials and methods** Based on an experience of 38 cases, we suggest an algorithm of treatment according to stage of pathology. Nineteen patients (18 males, 1 female, mean age 43 years) with prevalent radio-scaphoid (stage I) and scapho-capitate arthritis (stage II) were treated with partial scaphoidectomy and implant of a pyrocarbon ovoid (APSI). Patients with advanced collapse and luno-capitate arthritis underwent scaphoidectomy and intercarpal arthrodesis (17 male, mean age 48 years) or first row carpectomy (2 female, mean age 72 years). In all cases radial styloidectomy was performed. Patients were evaluated radiologically and clinically (VAS and DASH score, ROM and grip strength) at a mean follow-up of 35 months.
**Results** Better results (mean VAS score 0.8, mean DASH 9, mean ROM 75 % of the opposite wrist and grip strength 86 %) were obtained in patients with pirocarboin implant. One case showed an implant instability with dorsal subluxation. DISI deformity was present in some cases but didn't show a progression in the follow-up. Patients who underwent intercarpal arthrodesis showed a limited ROM (40 % compared to the opposite site), an average DASH score of 18 and a VAS score of 1.6. All arthrodesis appeared healed. One case, due to persistent pain, underwent radiocarpal fusion. 2 elderly patients had a proximal row carpectomy with acceptable functional results and relief of pain.
**Conclusions** According to these data a treatment algorithm is proposed based on the stage of pathology, age and functional request. In stage I and II implant of APSI lead most cases to complete functional recovery and must be considered the first choice of treatment. In more advanced stage proximal row carpectomy is considered only in elderly patients while intercarpal arthrodesis are performed in younger and active people. No differences in results were noted between partial or 4 corner arthrodesis.

### ORAL COMMUNICATIONS

### *C40–INFECTIONS*

### Total knee arthroplasty infections by resistant bacteria

M. Vasso*[1], A. Schiavone Panni[1], G. Gasparini[2], S. Cerciello[1], C. Fabbriciani[3]

[1]Università del Molise (Campobasso, IT);
[2]Università Magna Graecia (Catanzaro, IT);
[3]Università Cattolica del Sacro Cuore (Rome, IT)

**Introduction** The increasing number of total knee arthroplasties that are every year performed in all over the world, has resulted in a concomitant rise in bacterial infections. Controversy remains about the best management of the knee peri-prosthetic infections by resistant organisms. The purpose of this study was to determine if a two-stage reimplantation protocol was effective in eradicating infection and restoring a functioning prosthesis, and to report the percentage of patients who finally maintain an arthroplasty when resistant organisms are involved at the site of a an infected total knee arthroplasty.
**Materials and methods** Twenty-nine total knee arthroplasties infected by resistant bacteria were consecutively managed at the orthopaedic department of Catholic University (Rome, Italy). The patients included 21 females and 8 males with a median age of 72 (54–89) years. All patients were managed through a two-stage reimplantation strategy. Between the stages, intravenous antibiotics were administered for a median period of 8 (6–12) weeks. Median follow-up was 8 (5–11) years.
**Results** Two-stage reimplantation strategy resulted successful in 24 (83 %) patients to eradicate infection. Infection recurrence with the same organism occurred in 5 (17 %) patients. All these 5 patients did not finally keep a knee prosthesis.
**Discussion** Two-stage reimplantation certainly remains the best surgical solution for the management of the peri-prosthetic knee infections, with reported success rates almost constantly higher than 90 % in eradication of infection and preserving a functional prosthesis. However, these data have not been stratified on the aetiology of the infecting organism. When resistant bacteria are involved, overall outcomes certainly appear less reliable, with possible higher reinfection rates and final implant loss.
**Conclusions** The reinfection rate reported in the present study was quite similar to those in reports on two-stage reimplantation without organism stratification; therefore, two-stage protocol remains a viable option for patients with peri-prosthetic knee infections by resistant organisms. However, reinfections by resistant bacteria could be more frequent and to present severe complications, with higher rate of final prosthesis (and limb) loss.

### Hyperbaric oxygen therapy in the prevention of complications of opens fractures (nonunions, osteomyelitis)

R.A. La China*[1], V. Triolo[1], G. D'Amico[2], A. Triolo[3], A. D'Arienzo[1], M. D'Arienzo[1]

[1]U.O. Ortopedia e Traumatologia, Policlinico (Palermo, IT);
[2]Dipartimento di Anestesia e Rianimazione, Policlinico (Palermo, IT);
[3]U.O. Chirurgia Plastica Ricostruttiva ed Estetica, Policlinico (Palermo, IT)

**Introduction** In recent years there has been an increased incidence of open fractures due to the risk of high-energy trauma. The open fractures with any mortification of the soft tissue hesitate in 30–50 % of cases in nonunions and osteomyelitis. Numerous studies in literature have demonstrated the important role that hyperbaric oxygen therapy plays in supporting proper healing of bone and soft tissue as it has a positive effect on the neovascularization, on the reproduction of bone and antibacterial activity.
**Materials and methods** From January 2011 to December 2012 at our U.O. 16 cases of patients with open fractures of the lower limb were treated, including 4 cases associated with a large exposure with loss of substance of the soft tissue (Gustilio 3). All patients were treated in urgency by stabilization of the fracture, surgical debridement, OTI and already at the first post-operative day, they underwent broad-spectrum antibiotic therapy.
**Results** The 16 treated patients were subjected to clinical, radiographic examinations and serum-blood investigations up to 6 months. In 13 patients clinical and radiographic cure and the normalization of inflammatory markers were achieved. Only in one patient with trauma due to the crushing of a foot, after 15 sessions of OTI, we resorted to amputation of the first toe. The intervention of coverage of the loss of substance with vascularized free strip was necessary for two out of four patients with fracture associated with a serious loss of soft time.
**Discussion** This therapeutic protocol has allowed reduction of immediate post-operative complications in 96 % of cases, as well as promotion of good consolidation of the fracture in patients monitored for at least 6 months.
**Conclusions** The association OTI, broad-spectrum antibiotic therapy and surgery was successful in reducing the incidence of osteomyelitis and nonunion, ensuring a good revascularization and soft tissue healing, and also reducing the healing time of the fractures.

### Decennial clinical records of Codivilla-Putti Institute about the treatment of infected nonunions using Ilizarov's method

F. Centofanti[1], L. Fisichella*[2], R. Orani[1], P. Ditto[2], M. Maio[2], M.A. Rosa[2]

[1]Istituto “Codivilla Putti” GIOMI SpA (Cortina d'Ampezzo, IT); [2]Scuola di Specializzazione in Ortopedia e Traumatologia, Dipartimento di Scienze Biomediche e delle Immagini Morfologiche e Funzionali, Sezione di Ortopedia e Traumatologia, Università degli Studi di Messina (Messina, IT)

**Introduction** The increasing number of traumas and of the resulting surgical treatments in the orthopaedic field has characterized the growth of complications due to the surgical act itself, as the pseudo-arthrosis and infections. The result of the connection among multiple complications makes the problem worse to solve, since the orthopaedist should solve the entire problem. Authors mean to show the decennial clinical records concerning the treatment of infected pseudo-arthrosis, due to the failure of internal stabilization, using Ilizarov's method, founded on the stimulus of the revascularization of the infected site by corticotomy (osteomyelitis burns in the flame of the regenerate).
**Materials and methods** In this research 2 types of internal stabilization have been examined: plate and screws fixation and intramedullary nail fixation. Number of treated patients: 390; 286 with tibial location (54 intramedullary nail, 232 plates and screws), 104 with femur location (42 intramedullary nail, 62 plates and screws). In all of them it has been utilized the method of bone transport with Ilizarov's apparatus, which has been enhanced through adapted antibiotic therapies and specific diagnostic test, however linked to an adapted debridement of the infection's site. The transport has been monofocal in 286 cases and bifocal in 104.
**Discussion** After the decennial experience of the Putti Institute of Cortina d'Ampezzo, authors consider Ilizarov's method still actual in infected pseudo-arthrosis with bone loss treatments. In fact, thanks to a better stabilization and a multiplanar control, the Ilizarov's method allows the simultaneous management of different problems.
**Conclusions** This method should be addressed to the selected cases because it requires serial controls, a good compliance of the patient and longer times compared with other treatments. The Ilizarov's method allows to be drastic but not wreckers.

### A new approach to antibiotic prophylaxis in prosthetic joint surgery

M.C. Ferrari*, F. Astore, F. Della Rocca, F. Traverso, D. Ricci, M. Scardino, G. Grappiolo

Istituto Clinico Humanitas (Rozzano, IT)

**Introduction** Prosthetic joint infections are rare but devastating complications. Antibiotic prophylaxis is mandatory because of its efficacy to prevent such condition. Many different protocols are published in literature using first- or second-class cephalosporin or vancomycin/clindamycin in beta-lactamic allergic patients as well as in high methicillin-resistant *Staphylococcus aureus* incidence hospitals. The importance of short duration and proper timing of prophylaxis is well demonstrated.
**Materials and methods** From January 2011 continuous intravenous antibiotic prophylaxis was introduced starting in the operating theatre for a duration of 24 h using cefazolin or vancomycin. Infection data from 2009 to 2011 was evaluated with a follow-up ranging between 12 and 24 months.
**Results** In 2009 there was a 0.68 % of incidence of new cases (8 out 1,180 patients); in 2010 there was an increase to 1.19 % (16 out 1,350 patients); in 2011, with the new prophylaxis, the infections rate was 0.39 % (6 out of 1,545 surgeries) ($p = 0.012$).
**Discussion** In primary and revision total hip and knee arthroplasty, the duration of the procedure is often unpredictable. Therefore, we decided to use continuous 24 h using cefazolin or vancomycin intravenous antibiotic prophylaxis demonstrating a significant decrease in infections.
**Conclusions** The current prophylaxis for primary and revision total hip and knee arthroplasty should be considered.

### The sonication in microbiological diagnosis of prosthetic infections

F.R. Evola*[1], L. Costarella[1], V. Pavone[1], D. Bongiorno[2], L. Sessa[2], S. Stefani[2], S. Avondo[1], G. Sessa[1]

[1]Clinica Ortopedica dell'Università di Catania (Catania, IT); [2]Dipartimento di Scienze Microbiologiche, Università degli Studi di Catania (Catania, IT)

**Introduction** Prosthetic infections are one of the most serious and devastating complications in orthopaedics. The identification of the microorganism remains, therefore, an essential requirement in order to be able to undertake the most appropriate therapy. Recently, a role is given to the use of the sonicator; this instrument can break the biofilm prosthetic through the use of ultrasound at low energy (40–60 kHz for 5–30 min). This technique frees the microorganisms from the polysaccharide matrix, thus making them more easily cultivated. The purpose of this work is to evaluate the effectiveness of this method in the treatment of prosthetic infections.
**Materials and methods** We performed a prospective clinical study of 25 revisions of hip and knee arthroplasty infected with the objective to compare the bacterial isolation obtained by conventional culture of peri-prosthetic tissues and fluids with the one developed by the sonication of prosthetic implants removed. The standard samples were obtained by needle aspiration or intra-operative biopsy. The process of sonication, however, provides for the immersion of the prosthesis removed in physiological solution and the subsequent cultivation of the fluid so obtained using sonicated culture media for aerobic and anaerobic bacteria.

**Results** The study shows that the culture of samples obtained from the analysis of the surfaces of the prosthetic components proves to be more sensitive than that obtained from peri-prosthetic tissues. This method, however, has a lower specificity due to the risk of contamination during the phases of manipulation of the sample. By the sonication was possible in some cases more easily identify the pathogen involved, in other show additional populations involved in the infective process, which had not been detected with the standard method, in still others, the result was similar to that obtained from conventional diagnostic tests.

**Discussion** The microbiological diagnosis often does not allow the isolation exact infectious agent due to a low microbial load, of inadequate samples, of microorganisms incorporated into biofilm, bacteria difficult to cultivable. Today the microbiological diagnosis relies on the examination of the synovial fluid and biopsy samples of peri-prosthetic tissues. Sonication, as other methods that study the surface of the prosthesis, shows a higher sensitivity than the standard methodology, especially in the presence of antibiotic therapy and polymicrobial flora.

**Conclusions** The authors report preliminary data obtained on samples treated with prosthetic sonication, confirming that although this methodology is still under development and is burdened by false positives, it has a good chance of becoming a valid instrument for microbiological diagnosis of infections.

### Comparison of two different techniques for two-stage revision of total hip arthroplasty complicated by infection

M. Romagnoli*, M. Cadossi, E. Chiarello, A. Mazzotti, A. Shehu, S. Giannini

Istituto Ortopedico RIzzoli (Bologna, IT)

**Introduction** The two-stage revision in infected hip prosthesis is the most widely used and the most satisfactory treatment in terms of infection eradication. This technique can be performed either with the use of a temporary antibiotic-impregnated cement spacer or leaving the hip joint without any spacer till re-implantation of the hip prosthesis. The aim of our retrospective cohort study was to compare the efficacy of these different treatments in terms of infection eradication, complication rate and limb-length discrepancies associate.

**Materials and methods** Between 2009 and 2012 we performed 22 two-stage revisions for late infection in hip replacement. In 13 cases, (mean age was 72 years), we used an antibiotic-mix spacer, whereas in 9 cases (mean age was 60), we did not place any kind of spacer. We then evaluated the number of peri-operative complications, re-interventions, percentage of re-infections and dysmetria.

**Results** In those cases treated with antibiotic-loaded spacer we performed 4 re-operations for the spacer's replacement. There were two dislocations, two femoral fractures during the spacer replacement and an average limb-length discrepancy resulting in shortening of 0.8 cm. The average waiting time for the re-implantation was 5 months. In those cases treated without spacer we found no peri-operative complications, no neurological damage and a limb-length discrepancy resulting in shortening of 1.9 cm. The average waiting time for the re-implantation was 4 months and a half. With both techniques, at a 1 year follow-up, we found no re-infection.

**Discussion** Several advantages are reported related to the use of an antibiotic-impregnated spacer when an infected prosthesis is removed. These advantages include: preserving joint space, making re-implantation less demanding and avoiding an excessive shortening of the limb. However, we found a higher complications rate and even a greater number of re-operations in patients with the spacer. On the contrary, the technique without spacer use resulted in less complications although it was related to a much severe leg length discrepancy.

**Conclusions** With both techniques, the two-stage revision showed excellent results in eradicating the infection. Despite the limited number of patients and the short follow-up (1 year), our data suggest that avoiding the use of antibiotic spacer results in a lower complications rate with the only disadvantage of a more pronounced dysmetria.

### *C41–KNEE-PROSTHESES 1*

### Popliteus tendon role in CR and PS TKA stability: a cadaver study

U. Cottino*[1], M. Bruzzone[1], F. Olivero[1], D.E. Bonasia[2], F. Dettoni[1], A. Marmotti[1], R. Rossi[1]

[1]SCDU Ortopedia e Traumatologia, Ospedale Mauriziano Umberto I, Largo Turati 62 (Turin, IT);
[2]Dipartimento di Ortopedia e Traumatologia, Città della Salute e della Scienza, Presidio Ospedaliero CTO-M. Adelaide, Università degli Studi di Torino, Via Zuretti 29 (Turin, IT)

**Introduction** Popliteus tendon role in TKA biomechanic is still really controversial. Even if a lot of studies have been conducted on this topic, the results are not unique. We still don't exactly know its function and the effect on a TKA when inadvertently cut. The study was designed to identify the role of popliteus tendon in primary CR and PS TKAs.

**Materials and methods** We used 10 knees of 5 cadavers. On one side a PS design prosthesis was performed and a CR on the other side (NexGen, Zimmer, Warsaw, In, USA) randomly assigned. We measured with a digital caliper after tensioning with a dynamometric lamina spreader (90N) static flexion and extension gaps. We first measured the gaps with the intact tendon and then with the cut tendon again. The results were analyzed using the Student's *t* test.

**Results** After popliteus tendon resection we observed a statistically significative ($p < 0.05$) medial and lateral gap increase in flexion and extension in PS and CR designs. Gap increase was symmetrical in extension, in flexion we observed a greater increase in lateral gap. CR and PS data have been compared and we don't observed statistically significant differences between the two groups in flexion and extension.

**Discussion** Results obtained in our study suggest that in CR and PS primary TKAs popliteus tendon stabilizes the lateral compartment and in greater measure when in flexion. Preserving PCL in the central pivot do not influence lateral compartment stability in case of popliteus tendon resection.

**Conclusions** Popliteus tendon has an important role in TKA stability, in CR and PS design both. Intentional cut of the popliteus tendon has to be considered as a corrective step in severe valgus deformities only. Accidental resection of the tendon determines implant instability, in this case it is useful to utilize a more constrained TKA.

### The problems in the revision of unicompartmental prosthesis with total knee arthroplasty

D. Rosa*[1], C. Zorzi[2], V. Madonna[2], A. Russo[1], N. Auletta[1], V. Crispino[1]

[1]Dipartimento di Ortopedia, Università Federico II (Naples, IT);
[2]Ospedale Sacro Cuore, Don Calabria Negrar (Verona, IT)

**Introduction** The unicompartmental prosthesis represents a valid option for the treatment of osteoarthritis limited to only one of the compartments of the joint, most often confined to the medial compartment. Appears to have been well codified indications, contraindications, and surgical techniques related to unicompartmental arthroplasty. Nonetheless, it remains a discrete failure rate due to the loosening of the implant or to the progress of osteoarthritis of the other two compartments. The aim of this study was to analyze the technical difficulties encountered during the total knee replacement in unicompartmental tibio-femoral prosthesis outcomes.
**Materials and methods** We analyzed 33 revisions of unicompartmental tibio-femoral (28 medial and 5 lateral), performed between 2009 and 2011. Four patients of were treated for septic mobilization, 8 for arthritic progression, 12 for aseptic mobilization of one or more components, 4 for pain without mobilization, 2 for polyethylene wear, 1 for tibial plateau fracture and 2 for excessive laxity. In septic failure of unicompartmental prosthesis we performed two-step surgery. In one case we chose a constrained prosthesis, in the remaining cases we preferred a sliding systems bicompartmental (20 cases), CCK plant (8 cases) and hybrid systems (4 cases).
**Results** In almost all cases we have prescribed a standard rehabilitation protocol. We observed greater caution in cases where bone grafts have been used. The mean flexion was 110° in three patients we recorded a modest laxity, while in 8 cases we had stiffness, partially resolved with intensification of FKT. The subjective outcome was very satisfactory for 60 % of patients, satisfactory for 32 and unsatisfactory for 8 %.
**Discussion** The bone loss is the main problem, most frequently charged to the tibial side, especially in the case of metal-backed tibial prosthesis. Although less frequent, the loss of femoral bone is more difficult to treat, and involves major pitfalls especially in the choice of the rotation of the femoral component. We recommend to use intramedullary stems, especially if the defect is charged to cortical bone. We observed a lower utilization of prosthesis with a greatest constraint compared the prosthesis in the outcomes of HTO. In general it is more easy to solve a problem of infection than total replacement.
**Conclusions** An accurate preoperative planning helps to determine the complexity of the revision, noting that it is not particularly complicated surgery, but it requires precision, skill and experience.

### Is unicompartmental arthroplasty an acceptable option for spontaneous osteonecrosis of the knee?

D. Bruni*, I. Akkawi, F. Iacono, G. Raspugli, M. Gagliardi, M. Nitri, A. Grassi, M. Marcacci

[1]Istituto Ortopedico Rizzoli (Bologna, IT)

**Introduction** The literature suggests survivorship of unicompartmental knee arthroplasties (UKAs) for spontaneous osteonecrosis of the knee ranges from 93 to 97 % at 10–12 years. However, these data arise from small series (23–33 patients), jeopardizing meaningful conclusions. We determined (1) the longer-term survivorship of UKAs in a larger group of patients with spontaneous osteonecrosis of the knee; (2) their subjective, symptomatic, and functional outcomes; (3) the percentage of failures and reasons for failures to identify relevant indications, contraindications, and technical parameters for treatment with a modern implant design.
**Materials and methods** We retrospectively evaluated all 84 patients with late-stage spontaneous osteonecrosis of the knee who had a medial UKA from 1998 to 2005. All patients had preoperative MRI to confirm the diagnosis, exclude metaphyseal involvement, and confirm the absence of major degenerative changes in the lateral and patellofemoral compartments. The mean age of the patients at surgery was 66 years and mean BMI was 28.9. We conducted Kaplan–Meier survival analysis using revision for any reason as the end point. Minimum follow-up was 63 months (mean, 98 months; range, 63–145 months).
**Results** Ten-year survivorship was 89 %. Ten revisions were performed; the most common reasons were subsidence of the tibial component (four) and aseptic loosening of the tibial component (three). No patient underwent revision for progression of osteoarthritis in the lateral or patellofemoral compartments.
**Conclusions** Our data suggest spontaneous osteonecrosis of the knee may be an indication for UKA, provided secondary osteonecrosis of the knee is ruled out, preoperative MRI documents the absence of disease in other compartments, and there is no overcorrection in any plane.

### *C42–KNEE-PROSTHESES 2*

### Total knee arthroplasty in patients with extra-articular deformity

D. Vanni*, A. Pantalone, S. Raimondi, A. D'Apolito, G. Allevi, R. Campese, F. Fascione, E. Andreoli, V. Salini

Clinica Ortopedica e Traumatologica (Chieti, IT)

**Introduction** The implant of total knee arthroplasty (TKA) in patients with extra-articular deformity is a complex procedure. The deformity can be the result of malunion, metabolic disease, osteomyelitis or previous osteotomy. It can affect femur, tibia or both. It can involve coronal, sagittal, or transverse plane, appearing mono, bi or triplanar (Wang JW et al., J Bone Joint Surg Br 2010).
**Materials and methods** From January 2009 to January 2012, 5 semi-constrained arthroplasties (CCK) were implanted in patients with knee osteoarthritis associated with extra-articular deformity. In 4 patients the femoral deformity was the result of malunion due to a previous diaphyseal fracture and in 1 patient it was subsequent to osteomyelitis. In 4 cases the deformity was presented monoplanar (varus) and in one case, biplanar (varus and antecurvatum). An extramedullary alignment system has been used in 3 patients and an intramedullary alignment system in 2. A clinic (Oxford Knee Score) and radiographic (orthostatic) follow-up was performed at 1, 3, 6 and 12 months (Xiao-Gang Z et al., Int Orthop 2012).
**Results** There were no cases of implant failure, and no revision was performed. The mean Oxford Knee Score improved from a preoperative score of 17 pts to an average of 38 pts. Knee ROM improved from a preoperative value of 46° of flexion to an average of 102°, with complete extension recovery. There were no episodes of patellar dislocation, nor cases of patella baja or ligamentous instability.
**Discussion** In patients affected by gonarthrosis associated with extra-articular deformity does not exist only a surgical treatment strategy. It is possible to perform a corrective extra-articular osteotomy before or during the prosthesis implantation (Papagelopoulos PJ et al., Knee 2002) or an intra-articular asymmetric resection at the level of the joint (Wang JW et al., J Bone Joint Surg Am 2002). The extra-articular osteotomy, one or two steps, should be reserved only for cases of deformity in the coronal plane >20° in the femur and 30° in the tibia. Otherwise, it is appropriate to use an intra-articular asymmetric resection.

**Conclusions** TKA in patients with extra-articular deformity is a complex and difficult procedure. Asymmetric intra-articular resection represents an effective solution, able to ensuring satisfactory functional results in patients with monoplanar deformity below 20° for the femur and 30° for the tibia. A balance between the prosthesis constraint and the soft tissue balancing is mandatory (Papagelopoulos PJ et al., Orthopedics 2007).

### Intra-operative verification of planning with conventional instruments and surgeon's experience: bone resections, size and 3D alignment of the components

L. Matascioli*, V. Morea, D. Giglio, F. Terragnoli

Fondazione Poliambulanza (Brescia, IT)

**Introduction** PSI technology involves the creation of a three-dimensional anatomical model based on the patient's MRI, used to manufacture custom pin guides that conform precisely to the patient's anatomy. Purposes of this technology are optimization of mechanical axis and the femoral rotation, reduction of intra-operative bleeding and surgery duration. The aim of the study was to assess the achievement of those objectives.

**Materials and methods** Between June 2011 and September 2012, 50 patients underwent TKA surgery; all procedures were performed by our senior surgeon (F.T.) using PSI technology. Before surgery, all patient underwent MRI for 3D model creation. Final planning was performed after clinical re-evaluation of the patient considering full bearing anatomical axis, knee's range of motion, ligamentous laxity. Intra-operative measure of femoral and tibial bone resections were recorded. A comparison between planned and implanted component's size was performed. Femoral components rotation and thickness of the inserts were recorded too. Duration of procedures, amount of blood loss and transfusion rate were compared with those obtained using conventional technique.

**Results** We couldn't find significative differences between planned and verified bone resection; we had 12 re-cuts, (8 femoral and 4 tibial), 4 size changes (2 femoral and 2 tibial), 5 variation of the rotation, after control with standard technique. Those changes were affected by a manufacturing error, admitted by the manufacturer. Primarily were used 10 and 12 mm inserts (48 and 40 %, respectively). All post-operative measures of the HKA axis were situated in a range of 180° ± 3°, considered as satisfactory. Duration of surgery has been gradually reduced, becoming similar to that for conventional technique. Transfusion rate was 32 % for PSI vs 62 % for conventional technique.

**Discussion** Pre-operative plannings for PSI technology are reliable: there was a substantial accordance between planned and implanted component's sizes. Duration of operations gradually became comparable to those for conventional technique. Intra-operative bleeding was decreased.

**Conclusions** Radiographic study of the patient must be recent; planning must be performed considering clinical condition of the patients (software evaluates only bony parameters). Patient's MRI and manufacturing of custom pin guides affect the costs of this techniques; on the other hand, through pre-operative determinations, PSI technology can facilitate a reduction in conventional instrumentation requirements (2 boxes vs 6–8 boxes) increasing efficiency of the operating room. PSI technique may bring benefits to TKA surgery, but further investigations, involving larger series with long-term follow up, are required.

### Joint line restoration after primary and revision total knee arthroplasty

A. Rota*, P. De Santis, P. Rota, A. Aureli

Ospedale S. Pertini (Rome, IT)

**Introduction** Epidemiological studies show that from 1990 to 2003 the percentage of primary total knee arthroplasty had tripled to 100,000. Several studies show how the position of the joint line (position of the tibio-femoral joint space after total knee arthroplasty) has direct effects on postoperative outcomes (ROM, functional knee scores and stability); the joint line position have significant effects on the patello-femoral kinematics (rotation and abduction-rotation) but is not important for the tibio-femoral joint kinematics. The normal height of the patella preserving femoral bone stock or using femoral augmentation restores the joint line during TKA revisions.

**Materials and methods** From June 2005 to January 2011, at the Orthopaedics and Traumatology Department of Policlinico Casilino Hospital and S. Pertini Hospital (Rome), 196 patients with primary gonarthrosis were treated and 54 revisions of total knee arthroplasty (29 aseptic loosening, 22 infections, painful prosthesis) performed. In all cases, is valuated the radiographic joint-line pre-operative and post-operative and Knee Society Score (KSS).

**Results** The average follow-up is 13.5 months (range 7–21 months). We evaluated the clinical score in relation to the position of the joint line. If the joint line position is more than 4 mm, the average KSS is 125 points, if the joint line is <4 mm, and the mean score is 141 points.

**Discussion** Incorrect joint line changes the knee kinematics and favours the failure of primary total knee arthroplasty and revision (pain-painful prosthesis anterior subluxation of the patella, polyethylene wear, aseptic loosening).

**Conclusions** In agreement with the literature is important the distal femur managing, avoiding the proximal position of the femoral component that increasing the patello-femoral contact forces. Very important is the accurate preoperative planning (contralateral JL studying, reproducible radiographic landmarks, the joint line position between ±4 mm).

### Deformity correction in the conservative surgery of the knee: the tibio-femoral realignment

P. Barbato*, W. Leonardi

ARNAS Garibaldi (Catania, IT)

**Introduction** The deformities that are created are due to overloading joints that can occur: during the growth period with worsening of the deformity or deformity secondary compensation, after the growth with-arthritic degenerative changes affecting the medial tibial compartment, lateral patello-femoral.

**Materials and methods** For evaluation of osteoarthritis of the medial compartment, we used the classification of Ahlback changed. For the evaluation of articular cartilage lesion to Outerbridge. To pre-operative planning, we evaluated: the patient's age, occupation, clinical examination, X-ray in comparative limbs, TAC NMR trace the deformity on a transparent background for predict corrections. The axis correction tibio-femoral was executed by use of the FE (circular, hybrid, axial) with the technique of compattotomia. Patients treated from 1995 to 2012 are 173, aged between 35 and 72 years, follow-up: 1–10 years.

**Results** Range of deviation of the mechanical axis from the centre of rotation articular in varus deformity we have complied with the Fujisawa Point in valgus deformity of the load was distributed according to the diagram of Hsu. Joint pain: before correction was present, after was absent.

**Discussion** The factors that lead to arthritic knee varus decompensated are: failure of LCA resulting in anterior subluxation of the tibia, wear osteo-arthritic cartilage that will bring back postero-medial aspect of the tibial plateau. The care of osteoarthritis of the medial compartment must be secured in two basic stages: correction of axial deviation and roundabouts: osteotomy, arthroscopic treatment of intra-articular lesions.

**Conclusions** The bone and joint deformities of the knee should be corrected early to avoid overloading joints, responsible in young-adult degenerative arthritic alterations, in the elderly, the worsening state of decompensation resulting in arthritic joints. The femoral or tibial realignment is indicated up to a state arthritic grade 4 (Ahlback modified) and the names of the 70 years limit is not absolute, partly because the average life of the individual is exalted, the emicompattotomia angular distractive is the only method that allows to correct the deformity of the knee even after the patient has started to load; in the tibial or femoral realignment estimate especially the distance from the centre of rotation of the mechanical axis of the knee, the appropriate distribution of the loads, in two compartments of the knee qualify the good and the bad clinical functional outcome; the correction, performed in healthy adults, it can avoid the risk of knee arthroplasty in the elderly can only delay it, the weather arthroscopic aims to take away the pain immediately after surgery and improve joint function.

### *C43–TUMORS 1*

### Pedicled and free fibula grafts in primary reconstructions of the tibia: which and when?

M. Colangeli*[1], M. Manfrini[1], L. Campanacci[1], M. De Paolis[1], M. Ceruso[2]

[1]Istituto Ortopedico Rizzoli (Bologna, IT);
[2]Azienda Ospedaliera Universitaria Careggi-Chirurgia e Microchirugia Mano (Florence, IT)

**Introduction** The authors report their experience with vascularised fibula autograft (VFA), either free, or ipsilateral pedicled, in large tibia defects, analysing indications and results.

**Materials and methods** From 1994, VFA was used for tibia defects in 50 patients (median age 14 years, range 8–38) with bone sarcomas (44 cases with neoadjuvant chemotherapy, 3 with radiotherapy). In two patients, VFA was implanted alone, in one autografts were added. In 47 cases, VFA was associated to MBA. All cases had synthesis with plates. Defect ranged 9–22 cm, involving the diaphysis in 27 cases. In 21 cases, the resection included a proximal intra-epiphyseal osteotomy; in 2 patients involved a distal intra-epiphyseal osteotomy. In 24 cases contralateral VFA was harvested as free-flap and micro-anastomosis performed between fibular vessels and anterior-tibialis vessels of the recipient leg. In 26 cases ipsilateral VFA, harvested through postero-lateral approach opposite to medial approach used for resection, was transposed to fill the tibia defect, maintaining the vascularity. Implant outcome was investigated on serial radiology in 45 cases with 12 months follow-up. Function was evaluated according MSTS.

**Results** At median follow-up of 83 months, there are 42 disease-free survivors. Two patients died for toxicity and 6 of disease. There were 5 local recurrence: three were amputated, one revised with mega-prosthesis, one patient had local radiotherapy. There were 2 infections (one in each group) necessitating implant removal: both were reconstructed (one megaprosthesis, one Ilizarov technique). Mechanical complications (delayed union, fracture) occurred in 14 patients. Five healed without surgery. Nine patients were revised but only 2 (one in each group) had VFA removed and substituted with new grafts. These patients were the only ones with no changes in serial radiology. Functional analysis showed 80 % excellent and good results (78 % in free VFA and 82 % in pedicled VFA).

**Discussion** Both free and pedicled VFA have 95 % chance to maintain viability and mechanically adapt to tibia reconstructions. Association VFA/MBA: serial radiological analysis demonstrates intense remodelling suggesting biological, efficient and durable reconstructions.

**Conclusions** Pedicled VFA should be the first choice in diaphyseal defects but it's also effective in proximal intra-epiphyseal reconstructions. Previous radiotherapy, fractures or leg abnormalities may force to prefer free VFA.

### Surgical treatment of tibial metastases

B. Rossi, G. Scudieri, C. Graci, A. Ziranu, G. Maccauro*

Policlinico "Gemelli", Università Cattolica Sacro Cuore (Rome, IT)

**Introduction** Bone metastases are more frequent than primary tumours, but distal extremity locations are rare. Tibial metastases account for only 4 % and occur in an advanced stage of disease with predominant metaphyseal location. Treatment options vary from conservative treatment with chemo-radiotherapy and bisphosphonates to as much different surgical procedures, such as minimally invasive techniques, curettage and cement or resection and prosthetic reconstruction or amputation. Main indications for surgery are unique lesion, pathological fracture and prognostic favourable histotype. The aim of the study is to report our experience on surgical treatment of tibial metastases.

**Materials and methods** Eighteen patients with tibial metastases were treated from 1998 to 2012. Malignancies were breast (5 cases), kidney (4), lymphoma (2), bladder (3), myeloma (3), colon (2), stomach (1). Eight lesions were metadiaphyseal, 6 at the proximal epiphysis and 4 at distal tibia. Surgical planning options depended on the following parameters: location, histotype, visceral metastases, single or multiple bone lesions. Metadiaphyseal lesions in plurimetastatic patients were treated with locked intramedullary nailing. Proximally located epiphyseal lesions were treated with curettage and acrylic cement reinforced with pins. Unique lesions in the shaft and proximal metaphysis were treated respectively with intercalary and intra-articular resection and reconstruction with spacer and modular prostheses.

**Discussion** The median survival was 24 months (range 6 months–6 years). In most cases, implants were stable and durable. Almost all patients were satisfied with significant reduction of pain. There were no post-operative hardware failure nor infections or deep venous thrombosis. Only in 1 case the progression of local disease after intramedullary nailing has necessitated transfemoral amputation.

**Conclusions** Distal to elbow and knee metastases account for only 7 % of secondary long bones lesions. The most common histotypes at these sites are renal clear cell and lung carcinomas. Surgical treatment of metastases of the tibia is recommended in patients with a life expectancy >3 months, taking into account the staging of cancer disease and it should be minimally invasive except for some prognostic favourable tumours such as renal cell carcinoma. Appropriate surgery of tibial metastases ensures mechanical stability, improves the quality of life favouring early mobilization of cancer patients.

### Results and prognostic factors in 15 patients with peripheral dedifferentiated chondrosarcoma

P. Ruggieri[1], E. Pala[1], E. Henderson[1], E. Rimondi[2], M. Hassani[1], A. Piccioli[3], M. Maraldi[1], C.N. Abati[1], S. Giannini[1]

[1]III Department of Orthopaedics, University of Bologna, Istituto Ortopedico Rizzoli (Bologna, IT);
[2]Department of Radiology, Istituto Ortopedico Rizzoli (Bologna, IT);
[3]Department of Orthopaedic Oncology, CTO (Rome, IT)

**Introduction** Dedifferentiated chondrosarcoma is an uncommon tumour that is known to arise from pre-existing, low-grade cartilage lesions. Peripheral dedifferentiated chondrosarcomas (PDC) arise from pre-existing exostoses, or extracortically, and may appear as a peripheral chondrosarcoma without the features of its dedifferentiated counterpart. Dedifferentiated chondrosarcoma has a very poor prognosis. Aim of this study was to evaluate the survival of patients with peripheral PDC and to evaluate possible prognostic factors.
**Materials and methods** Between 1980 and 2006, 15 patients were treated for PDC: 11 males and 4 females, mean age of 42 years. In 1 case tumour was located in the humerus, in 3 in distal femur, in 1 in emianterior chest, in 5 cases in ileums, in 2 in scapula, in 2 proximal femur, in 1 proximal fibula. The de-differentiation was in malignant fibrous histiocytoma in 9 cases, osteosarcoma in 5 cases and spindle cell sarcoma in 1 cases. Fourteen patients received surgery (one patients was not operable for multiple distant metastases): tumour resection in 9 cases, amputation in 5. Chemotherapy was given to 8 patients.
**Results** Four patients (26.6 %) were NED at a mean follow-up of 14.7 years and 11 patients DWD at a mean time of 2.6 years. The overall survival of patients was 34 % at 10 years. There was no significant difference in survival between patients with PDC of the trunk and those with PDC of the extremities ($p = 0.2397$). There was no significant difference in survival with chemotherapy and surgery or with surgery only ($p = 0.6269$).
**Discussion** The literature describes many factors that significantly influence prognosis: presence of metastases at diagnosis, tumour volume, histology of the de-differentiated component, age at diagnosis, resection margins and pathological fracture.
**Conclusions** The prognosis for patients with PDC remains dismal. Surgery with wide margins remains the principal treatment for this condition. There was no statistical evidence of any beneficial effect from chemotherapy.

### Ewing sarcoma of the sacrum: clinical outcome of 19 patients from a single institution

P. Ruggieri[1], X. Sun[1], G. Drago[1], A. Angelini[1], C. Romagnoli[1], M. Maraldi[1], A. Piccioli[2], M. Hassani[1], S. Giannini[1]

[1]III Department of Orthopaedics, University of Bologna, Istituto Ortopedico Rizzoli (Bologna, IT);
[2]Department of Orthopaedic Oncology, CTO (Rome, IT)

**Introduction** Ewing's sarcoma occurs rarely in the sacrum with incidence of 1–2 %. Although overall results of treatment of Ewing's sarcoma have improved with multimodal strategies, unfortunately, in the sacrum it has worse prognosis than in other sites. A retrospective analysis describes our experience with respect to oncological outcome and neurologic function.
**Materials and methods** We retrospectively reviewed 19 patients with Ewing's sarcoma of the sacrum treated between September 1980 and December 2011. Pain and neurologic impairment were the most common symptoms. The mean duration of symptoms was 7.8 months. Three patients received surgery with or without radiation and chemotherapy. One patient had radiotherapy alone. Chemotherapy was given to 18 patients, in 10 of them followed by radiation.
**Results** The mean follow-up was 7.26 years (range 6 months–27 years). In 2 cases we performed surgery, both of them developed local recurrence. Seven patients had metastases at diagnosis while other 5 patients developed metastases during follow up. Overall 13 patients died at mean of 4.72 during the follow-up. The 5 year overall survival (OS) and the 5 year event-free survival (EFS) were respectively 47.3 and 31.5 %. Gender and age did not appear to influence OS or EFS statistically.
**Discussion** The outcome of Ewing's sarcoma of the sacrum was unrelated to gender, age, metastasis at diagnosis and local treatment strategy. The data collected do not allow to determine the actual efficacy of local radiotherapy or surgical treatment.
**Conclusions** Our experience showed that although multimodal treatment could improve the overall survival, Ewing's sarcoma of the sacrum had a significantly worse outcome than in other primary locations.

### Total femur prosthesis for reconstruction after sarcomas resection

P. Ruggieri[1], G. Drago[1], E. Pala[1], G. Trovarelli[1], T. Calabrò[1], A. Angelini[1], A. Piccioli[2], C. Romagnoli[1], S. Giannini[1]

[1]III Department of Orthopaedics, University of Bologna, Istituto Ortopedico Rizzoli (Bologna, IT);
[2]Department of Orthopaedic Oncology, CTO (Rome, IT)

**Introduction** The choices of treatment for patients with extensive tumours of the femur include total femur mega-prosthesis or large allograft-prosthetic composites. Previous reports suggested variable survival ranging from 60 to 70 % at 1–2 years. However, these studies described earlier prostheses and techniques. To confirm previous reports we determined risk of local recurrence, overall survivorship and function in patients with total femur reconstructions for tumours.
**Materials and methods** We retrospectively reviewed 26 patients with total femur mega-prostheses implanted between 1987 and 2010 after resection of bone tumours. Two patients lost at follow-up were excluded; the remaining 24 included 15 males and 9 females with a mean age of 27.2 years. The mean follow-up was 5.3 years (range, 5 months–23 years). Function was assessed according to the MSTS system II.
**Results** One patient developed a local recurrence during follow-up (4.1 %). At last follow-up, ten patients were continuously disease free at a mean of 11.1 years, one patient had no evidence of disease after treatment of a recurrence, another patient had no evidence of disease after treatment of a pulmonary metastasis, and 12 patients died of their disease at a mean time of 1.5 years. In 21 patients evaluated with the MSTS score, the mean score was 68.41 %; seven patients had over 75 %, eleven from 51 to 75 %, three from 26 to 50 %. Four patients (16.6 %) had complications requiring further surgery in absence of trauma. A fifth patient had a post-traumatic peri-prosthetic fracture.
**Discussion** According to the literature, our results showed that a total femur prosthesis allows a limb-preserving procedure in tumours with extensive femoral involvement or in the presence of a skip lesion along the femur.
**Conclusions** The prognosis of these tumours requiring total femur resection is poor, but this reconstruction provides good function with a relatively low rate of major complications.

### *C44–TUMORS 2*

### Proximal humerus reconstruction in bone tumor surgery: osteoarticular allografts versus allograft-prosthesis composites in different techniques

D.A. Campanacci*, G. Beltrami, G. Caff, P. Cuomo, N. Mondanelli, R. Capanna

Ortopedia Oncologica (Florence, IT)

**Introduction** After proximal humerus tumour resection there are different limb salvage reconstructive options including prostheses, massive allografts or a combination of both techniques. Osteoarticular massive allografts (OA) allow a biologic reattachment of tendinous structures of the host providing stability and favourable functional recovery. The rational of allograft-prosthesis composites (APC) is to associate functional advantages of massive allografts to long term durability of prosthetic implants, resulting in lower fracture and failure rate with time. The objective of the present study was to compare osteoarticular allograft with APC reconstruction with different assembling techniques, evaluating functional outcome, complications and survival of reconstruction.
**Materials and methods** Between June 1996 and January 2011, 49 patients at an average age of 33 years (7–75) received an OA or APC of the humerus following resection of a primary bone tumour. The series of patient was divided in groups according to different assembling techniques: OA filled with cement (14); total OA humerus filled with cement (2); APC with resurfacing prosthesis (4); long stem APC (9); long stem reverse APC (9); short stem reverse APC with plate fixation (2); total humerus APC using reverse shoulder prosthesis and conventional elbow prosthesis (1). Complication rate of different techniques was evaluated and correlation between MSTS functional score and different assembling technique was assessed. The mean duration of follow-up was 57 months (4–164).
**Results** The following complications occurred: diaphyseal fracture in cemented OA (12 %, 2 of 16); subchondral fracture in cemented humeral OA (6 %,1 of 16); non-union in proximal humerus OA (21 %, 3 of 14) and in conventional long stem APC (11 %, 1 of 9); dislocation of conventional long stem APC (11 %, 1 of 9) and long stem reverse APC (9 %, 1 of 11); prosthetic loosening in long stem reverse APC (22 %, 2 of 9). These complications were observed at a median FU of 38 months (14–93). The mean MSTS score was 77 % (47–90 %).
**Discussion** Nonunion and diaphyseal fracture are frequent in OA of the humerus leading to implant failure with time. Instability seems to be the early problem of artificial prosthesis. Aseptic loosening was the major revision cause in cemented long stem reverse APC. Short stem APC allows to maintain bone stock but nonunion may occur.
**Conclusions** APC with resurfacing prosthesis is particularly indicated in children in order to preserve glenoid and distal humerus bone stock.

### Regenerative medicine in the treatment of pseudotumoral lesions: polytherapy role

P. De Biase[1], D.A. Campanacci[2], R. Capanna[2], O. Pecchioli[2], P. Cuomo[2], G. Scoccianti[2]

[1]Azienda USL 8 Po San Donato (Arezzo, IT);
[2]AOU Careggi (Florence, IT)

**Introduction** The treatment of pseudotumoural cavity lesions has seen an increase of studies on the use of growth factors and autologous bone marrow concentrate. These methods can be used with open surgical techniques or by percutaneous techniques. The purpose of the study is to present a decade of experience in the use of these methods of regenerative medicine.
**Materials and methods** We reviewed 122 cases of pseudotumoural cavity lesions of bone, corresponding to 114 patients (72 males, 42 females) which we treated from 2000 to 2012 with an enriched graft. In detail we studied 66 aneurismal bone cysts; 45 unicameral bone cysts; 10 aneurismal bone cyst associated to fibrous dysplasia; 7 cases stage I giant cell tumour; 2 cases of benign fibrous histiocytomas; 5 chondroblastomas; 7 nonossifying fibromas; 1 Ewing sarcoma; 1 desmoid tumour and 1 adamantinoma. All patients were treated with a graft enriched with an autologous concentrated bone marrow obtained always with two different procedures (SEPAX and Regen) based on the amount required. To reproduce the osteoinductive and osteoconductive component required for bone regeneration, to platelet-rich plasma, DBM, bone grafts autologous, homologous or synthetic were added; individually or in combination.
**Results** In total, we obtained a recovery in 68.85 % of cases with no distinction between the two methods of bone marrow aspiration. In particular, the addition of the DBM has led to a recovery of 70 %, while using in combination an osteoconductive has increased the cure rate up to 79 %; cases where the platelet-rich plasma had also been used observed a healing of 87 %.
**Discussion** Summarizing the data collected we can say that the techniques of tissue engineering applied by us were safe and effective for the patient which open up developments and applications in other diseases. Among the additions of other growth factors to concentrated bone marrow, the PRP is the most effective one, while the DBM has not shown better results.
**Conclusions** In conclusion, we can say that, after more than 10 years of using a graft enriched with autologous bone marrow concentrate and other products such as autologous platelet-rich plasma, the surgical technique has proven to be safe for the patient, non-invasive and effective.

### Morbidity rate in en bloc resection of spinal tumors. Long-term results of 188 cases

S. Colangeli*, A. Gasbarrini, S. Bandiera, G. Barbanti Brodano, S. Terzi, R. Ghermandi, L. Babbi, S. Boriani

Istituto Ortopedico Rizzoli (Bologna, IT)

**Introduction** En bloc resection is described as the surgical procedure aiming to remove the tumour as a whole, fully covered by a continuous shell of healthy tissue, called margin. The most aggressive the tumour, the most important the margin for local control and for final outcome, more acceptable functional sacrifices, as balanced by tumour control. In case the margins include relevant anatomical structures, a functional loss of different gravity will follow the wide resection. Aggressive benign tumours and malignant tumours are accepted indications for en bloc resection.
**Materials and methods** En bloc resection was performed in 188 out of 1,247 patients surgically treated for spinal tumour from 1990 to 2012 (98 males, 90 females, age 44 ± 18 years). One hundred and thirty-four patients were affected by primary tumours, 54 by metastases. The mean follow-up was 5 years (0–214 months). Reconstructive surgery of the anterior column was performed using carbon or titanium prosthetic implants, massive allografts or combination the two previous systems.
**Results** The major surgical complications (bleeding/hematoma, dural, pleural or peritoneal lesions, pulmonary embolism) were 49 (26 %), 3 patients (1.6 %) died (1 death for intra-operative injury of the vena cava, 1 death in the immediate post-operative for pulmonary complications, 1 death for post-haemorrhagic anaemia). Surgical minors complications (dehiscence of wounds, loosening of screws or breaking of the rods during follow-up) were 29 (15 %). The principle factors that influenced morbidity were: multi segmental resections, double surgical approach and previous surgery.

**Discussion** En bloc resection is a demanding procedure and requires a careful planning after a well conducted decision making process. The patient has to correctly understand the purpose of the surgery—based on oncological staging—in order to accept—or not—morbidity and functional loss as counterbalanced by the expected final result.

**Conclusions** The severe morbidity of en bloc resection must be considered in the decision making process of bone tumours of the spine. Further, the functional loss consecutive to sacrifice of structures invaded by the tumour or to be resected to provide the required margin should always be counterbalanced by the expected oncologic outcome, as the local control is the primary target of tumour surgery. Intentional transgression to oncologic principles and margin violation can be considered for saving anatomical structures and related functions if required by the patient. In these cases, combination with radiotherapy or medical oncology can be considered.

### Surgical resection of sacral chordoma: an update of the Rizzoli experience on 71 cases

P. Ruggieri[1], A. Angelini[1], G. Drago[1], F. Jorge[1], C. Romagnoli[1], A. Piccioli[2], A. Ferraro[1], S. Giannini[1]

[1]III Department of Orthopaedics, University of Bologna, Istituto Ortopedico Rizzoli (Bologna, IT);
[2]Department of Orthopaedic Oncology, CTO (Rome, IT)

**Introduction** Our aim was to analyze the outcome in a large series of patients with sacrococcygeal chordoma at long-term follow-up, in order to help to define the role of previous inadequate surgery, surgical margins and the relationship with local recurrences.

**Materials and methods** We retrospectively reviewed 71 patients with sacral chordomas treated with surgical resection. Forty-eight resections were proximal to S3, 23 below or at S3. Proximal resections required an anterior and posterior approach with the exception of the 8 patients treated with a new technique using a posterior approach only, as well as 3 cases of distal resection. Eleven received previous intralesional surgery elsewhere. Three reconstructions were performed.

**Results** Margins were wide in 44 resections, wide but contaminated in 11, marginal in 9 and intralesional in 7. Patients previously treated had wide margins in 7 cases, wide but contaminated in 2, marginal and intralesional in one each. Three patients died for postoperative complications and were excluded from further analysis. Overall survival was 92, 65 and 44 % at 5, 10 and 15 years respectively. At a mean follow-up of 9.5 years (0.5–32 years) 37 are NED (54.4 %): 27 continuously NED, 5 $NED_1$, 1 $NED_2$, 4 died of different disease. Twenty-three died with disease (33.8 %) and 8 are alive with disease (11.7 %). Relapses included 15 local recurrences, 6 distant metastases, 17 both. Local recurrence rate was strictly related with margins. Local recurrence rate was significantly higher in patients that received previous intralesional surgery ($p = 0.0217$). Factors that influence local recurrence rate were margins other than wide ($p = 0.0339$) and tumour volume at different cut-offs ($p < 0.01$), whereas level of resection was not significant ($p = 0.5883$). Multivariate analysis confirmed the role of tumour volume. Complication rate was high (80.9 %) with an infection rate of 41.2 %.

**Discussion** The treatment of choice in sacral chordoma is surgical resection. Wide margins obtained at initial surgery are the primary factor to improve survival. Complication rate is high, also without a sacral reconstruction.

**Conclusions** The most prominent adverse factor for local recurrence was previous intralesional surgery. Local recurrence rate was related with inadequate margins and tumour volume. Oncologic outcome of major resections is comparable to minor.

### Proximal humerus reconstruction with MRS Bioimpianti prostheses after resection of bone tumors: an analysis of 255 cases

P. Ruggieri, T. Calabrò, A. Angelini, C.N. Abati, E. Pala, M. Hassani, M. Maraldi, I. Piraino, S. Giannini

III Department of Orthopaedics, University of Bologna, Istituto Ortopedico Rizzoli (Bologna, IT)

**Introduction** Limb salvage using endoprosthesis is considered as a treatment of choice for bone sarcoma involving the extremities with relatively low incidence of major complications. Modular prosthetic reconstructions is the most frequently used type of reconstruction after resection of the humerus. Aim of this study was to review the experience of the Rizzoli Institute with prosthetic reconstruction after resection of bone tumour in the humerus.

**Materials and methods** Between 1975 and 2010, 255 modular prostheses (alone or in association with allografts) type MRS of the proximal humerus were implanted. Population included 154 males and 101 females with mean age 40 years (range 5–81). In two patients tumour involves humeral diaphysis, in all others the proximal part. Histology showed 91 osteosarcomas, 52 chondrosarcomas, 61 metastatic carcinomas, 10 GCT, 10 MFH, 9 Ewing's sarcoma, 22 other diagnoses.

**Results** Major complications causing implant failure were infections (19 cases, 7.4 %), aseptic loosening (4 cases, 1.5 %) and breakages (3 cases, 1.2 %). Local recurrence occurred in 8 patients (3 %). Survival in patients with primary tumours was 35 % at 10 years and survival in patients with metastasis was 3 % at 10 years. Implant survival to all major complications was more than 80 % at 10 years and 20 years.

**Discussion** The reconstruction of the proximal humerus with modular endoprotheses is the treatment of choice in all patients in whom, for adequate surgical margins, it has become necessary the sacrifice of the rotator cuff, deltoid, and the axillary nerve. The high incidence of infection, in agreement with the literature, may be in part due to poor coverage of the implant with soft parts. Despite many times this prosthesis acts as a simple spacer, is well accepted by patients both from the point of view of aesthetic and functional.

**Conclusions** This prosthesis is actually a simple spacer, therefore it is indicated for resections of tumours where it is not possible to spare the abductor apparatus (deltoid, axillary nerve, rotator cuff). Otherwise we prefer different reconstructions. This simple modular prosthesis provides satisfactory results, but not abduction.

### *C45–TUMORS 3*

### Surgical treatment of the cervical spine tumors. A retrospective study on 251 out of 1,247 patients surgically treated from 1990 to 2012

S. Colangeli*, A. Gasbarrini, R. Ghermandi, G. Barbanti Brodano, S. Bandiera, S. Terzi, L. Babbi, S. Boriani, A. Lampner

Istituto Ortopedico Rizzoli (Bologna, IT)

**Introduction** Excluding metastases and plasmacytomas, most bone tumours of the cervical spine are benign. The surgical treatment of bone tumours of the cervical spine, when indicated, is difficult and frequently requires multiple approaches. Intralesional surgery, combined with modern techniques of radiation therapy, or radiation alone, are frequently an option in malignant tumours. Intralesional surgery or minimally invasive techniques such as percutaneous radiofrequency ablation can also effectively replace surgical excision in some cases of benign tumours.

**Materials and methods** Two hundreds and fifty-one patients (182 suffering from primary tumours and 69 metastases) of 1,247 were surgically treated from 1990 to 2012 (139 males, 112 females, age 18–44 years). The surgical techniques used were en bloc resection in 3 cases (2 chondrosarcoma, 1 chordoma), debulking and stabilization in 139 cases (55.5 %) and decompression + stabilization in 109 cases (43.5 %). The mean follow-up was 5 years (min 1–513 months).

**Results** The major surgical complications (respiratory problems for glottis oedema, pulmonary embolism, dysphagias) were 28 (11 %) including 2 peri-operative deaths (1 pulmonary embolism, 1 respiratory failure). The minor surgical complications (wound dehiscence, loosening of the screws or breakdown of the rods during follow-up) were 21 (8.5 %). The local control of the disease was achieved in 90 % of cases, local recurrences in fact were 26 (10 %), 17 of which required a new surgical treatment. In the other 9 cases palliative radiotherapy was performed. In all patients, comparison between the pre-operative neurological status and the post-operative one, evaluated by Frankel classification, showed no aggravations, while a clinical improvement was observed in approximately 78 % of cases.

**Discussion** Oncological and surgical staging are mandatory to decide about the treatment. Adjuvant therapies like radiation, embolization and chemotherapy must be considered by the multidisciplinary team. The patient has to correctly understand the purpose of the surgery—based on oncological staging—in order to accept—or not—morbidity as counterbalanced by the expected final result.

**Conclusions** An adequate treatment for cervical spine tumours is mandatory at the first time. Incomplete or inadequate treatments that expose the patient to the risks of recurrence or, even worse, an adverse survival outcome must be avoided. On the other hand, overaggressive treatments may expose a patient at unnecessary risks without an appropriate oncological indication. Diagnosis, staging and treatment should be centralized in a referral centre where all necessary information should be obtained before any invasive intervention, including biopsy.

### A database for bone and soft tissue tumors: it is necessary for a reference centre, but which are the problems and the objectives?

M. Boffano*, U. Albertini, E. Boux, S. Marone, A. Ferro, R. Piana

S.C. Ortopedia Oncologica e Ricostruttiva, AO Città della Salute e della Scienza (Turin, IT)

**Introduction** Primary bone and soft tissue tumours are rare. Their treatment should be centralized from the beginning (diagnosis and imaging) in a reference centre following national and international guidelines. The aim of this study is to demonstrate that centralization improves the prognosis and lowers complication rate.

**Materials and methods** The expertise of a national reference centre for the diagnosis and treatment of bone and soft tissue sarcomas goes through a management database for data collection and treatment schedule. In this study 4 different databases have been evaluated. Two databases were realized with Access (Windows Office, Microsoft), one with a dedicated software and secondarily converted in Active HTML, and one using the institutional software for the management of inpatient/outpatient treatments all over the hospital. Creation and management of a waiting list, data recovery for scientific purpose, data collection have been evaluated. Furthermore patients lost at follow-up and the delayed call of a patient in waiting list have been evaluated as potential pitfalls.

**Results** No tested database fully responds to all the criteria of treatment management, data collection and recovery for scientific purpose. The softwares altogether represent a good compromise but also a big loss of time.

**Discussion** The necessity to add many entry variables demonstrates the complex management of bone and soft tissue sarcomas, even if rare tumours. The number of patients in a reference centre is huge. The accuracy in data collection and data updating is fundamental for scientific purpose but often one or more data managers are necessary. Also updating stadiation and treatment information requires a dedicated nurse/physician. The experience of international reference centres confirms these problems.

**Conclusions** At present there is no internationally recognized database because it often reflects the necessities of the local system. The necessity to use an institutional software for inpatient/outpatients management represents a big loss of time. The common experience of reference centres in patient management and data collection softwares could improve, if compatible, multicentric studies and case series.

### Electrochemotherapy: a new strategy against the cancer

M. Gallo*[1], G. Colella[2], F. Fazioli[1]

[1]INT “Fondazione Pascale” (Naples, IT);
[2]AOU Federico II (Naples, IT)

**Introduction** Electrochemotherapy (ECT) is a local treatment that is based on electroporation of the cellular membranes, through the local application of intense and short electrical pulses that reversibly permeabilize the cell membranes facilitating the entry of the drug into the tumour cells. This system allows the administration of low doses of antiblastic drug (bleomycin, cisplatin) whose cytotoxic activity is strongly enhanced by the electroporation.

**Materials and methods** We treated with ECT 12 patients who had different types of injuries, including: a verrucous carcinoma of the gluteus, a dermatofibrosarcoma of the shoulder, a coccyx bone metastasis.

**Results** The response to the therapy has proved particularly interesting in these patients, as there was a significant regression of the lesions and a complete stability of the secondary bone lesions after 90 days from the treatment. We therefore believe that this technique is particularly interesting for both the good pain control, both for local control of the disease. Furthermore, the complete absence of complications has allowed us to discharge the patients from a distance of 24 h after treatment.

**Discussion** The procedure, that requires a general anaesthesia, consists of administering the antiblastic drug intravenously and then, after about 8 min, performing the electroporation. Electrochemotherapy is a simple treatment lasting a maximum of an hour. It results to be efficient even after one single session. It allows to preserve the margins of healthy tissue and the organ function. Thanks to electroporation you can use reduced doses of medication.

**Conclusions** The electrochemotherapy is a technique easy to use and extremely efficient both for what concerns the control of pain that for local control of the disease.

### Ultrasound guided needle biopsy: a reliable option for bone lesions? Preliminary results

S. Marone*[1], A. Ferro[1], U. Albertini[1], M. Boffano[1], A. De Marchi[2], S. Pozza[2], A. Linari[3], C. Faletti[2], R. Piana[1]

[1]SC Ortopedia Oncologica e Ricostruttiva, AO Città della Salute e della Scienza (Turin, IT);
[2]Dipartimento di Diagnostica per Immagini, AO Città della Salute e della Scienza (Turin, IT);
[3]Servizio di Anatomia Patologica, Ospedale OIRM/S. Anna (Turin, IT)

**Introduction** Needle bone biopsy is usually performed under CT guidance. Compared to US guided biopsy, CT guided needle biopsy is extremely precise but takes much more time in execution and, in same geographical regions, is difficult for less available CT machines. The aim of this study is to propose US guided needle biopsy for bone lesions presenting with bone breech in the cortex without soft tissue involvement.
**Materials and methods** From January till December 2012, on 11 patients (7 males, average age 57 years, range 28–81) an us guided tru-cut biopsy was performed for a bone lesion. All the lesions presented with a bone breech in the cortex and were located in the lower limb (3 cases), upper limb (4), chest wall (3), and pelvis (1). MyLab Twice sonography (Esaote, Genova, Italy) with multifrequency probes and sterilizable biopsy kit was used. In 9 patients only a tru-cut needle had been used, whilst in 2 cases was necessary to take same samples also with a trephine bone needle. The agreement between bioptic and final diagnosis (either histological or clinical diagnosis, if a surgical treatment was not necessary) was evaluated in order to assess the diagnostic accuracy (Cohen's kappa coefficient).
**Results** In 9 cases out of 11 the diagnosis was correctly obtained: myeloma (3 cases), metastases of carcinoma (2), metastases of sarcoma (2), giant cell tumour of the bone (2), Tietze's disease (1). In 2 cases there was a not diagnostic sample. On 11 patients 5 histological types were diagnosed, so that 5 items were considered for Kappa coefficient calculation. In the remainder 9 patients a perfect agreement was observed ($k = 1$, $p < 0.05$).
**Discussion** US guided needle biopsy is a faster technique and does not require ionizing radiations. The learning curve is similar to CT guided biopsy. Often, even in reference centres, the choice of imaging technique in bioptic guidance is conditioned by the availability of Imaging instrumentations.
**Conclusions** In selected cases a bone biopsy can be performed under US guidance. Further studies are necessary to confirm our results.

### Tumours of the foot: epidemiologic analysis and principles of treatment. The Rizzoli Institute experience

P. Ruggieri[1], A. Angelini[1], F. Jorge[1], X. Sun[1], G. Guerra[1], E. Pala[1], I. Piraino[1], A. Piccioli[2], S. Giannini[1]

[1]III Department of Orthopaedics, University of Bologna, Istituto Ortopedico Rizzoli (Bologna, IT);
[2]Department of Orthopaedic Oncology, CTO (Rome, IT)

**Introduction** Tumours of the foot are rare. Although most of these are benign, a failure to appreciate their presence may delay diagnosis and treatment. The knowledge of differential diagnosis and an appropriate pre-operative planning are the most important factors for adequate treatment. Aim of this study was to evaluate the incidence, histological features and treatment strategy of the most common tumours of the foot.
**Materials and methods** From 1900 to 2009, 1,170 tumours of the foot were retrospectively analyzed. Imaging included radiographs in all patients, and CT and MRI when available. Diagnosis was established in all cases with biopsy and histological slides were reviewed. There were 189 and 981 soft tissue and bone lesions, respectively. Localizations were phalanges (240; 20 %), metatarsal region (245; 21 %) and hindfoot (685; 59 %). Benign or pseudotumoral lesions were 870 (74 %): multiple chondromas (168), osteoid osteoma (164), solitary osteochondroma (47), Nora disease (78), calcaneal cyst (51), aneurysmal bone cyst (45) were the most frequent lesions observed. Malignant lesions were 300 (26 %): Ewing's sarcoma (44), central chondrosarcomas (29), metastatic carcinoma (24) and other more rare entities.
**Results** Benign and pseudotumoral lesions are generally treated with curettage with and without bone grafting. Neoadjuvant and adjuvant chemotherapy associated with surgery, is required for responsive malignant lesions. Amputation may be required for tumours involving the hindfoot.
**Discussion** Clinical trials on numerous series have shown that the incidence of benign and malignant tumours of the foot and ankle is increased compared to the past. A careful anamnestic, clinical and imaging analysis is needed to diagnose and treat any lesion of the foot, including those lesions with a seemingly regular course. In consideration of the anatomy of the foot, tumours often invade other compartments than that of origin, thus making indispensable an early diagnosis. Unfortunately, many tumours of the foot are still diagnosed too late even though the symptoms are relatively early compared to lesions in other locations. Malignant tumours of the foot are rare and often their malignant potential is underestimated.
**Conclusions** Malignant tumours are relatively rare, but a high level of attention on imaging and clinical examination is required, even when diagnosis seems straightforward. With few exceptions, a biopsy is recommended before proceeding to surgery.

---

### *C46–HIP 1*

### Metal on metal total hip replacements: functional and radiographic evaluation and evaluation of serum levels of metal ions up to a 5-year follow-up

A. Moroni*[1], D. Leonetti[2], M.T. Miscione[3], R. Orsini[3], G. Micera[3]

[1]Dipartimento di Scienze per la Qualità della Vita, Università di Bologna (Bologna, IT);
[2]Ospedale Maggiore Bologna (Bologna, IT);
[3]Istituto Ortopedico Rizzoli, Universita di Bologna (Bologna, IT)

**Introduction** Metal-on-metal total hip replacement with (MOM-PTA) allowing the use of large diameter femoral heads reduce the risk of dislocation and play a physiological joint biomechanics with greater range of motion. However it has recently been reported in the literature an increase in the serum level of metal ions due to corrosion phenomena that occur between the collar of the prosthesis and the femoral heads. The aim of this study was to retrospectively evaluate a series of 53 patients operated on for PTA-MOM by measuring serum levels of chromium (Cr), cobalt (Co) functional and clinical outcomes after 5 years of implantation.
**Materials and methods** We retrospectively evaluated 53 patients, mean age of 64 ± 3 years, operated with cementless hip prosthesis. In all cases we used a system consisting of: synergy stem, femoral head BHR and cup BHR of cobalt chromium alloy. All patients, at the last follow-up were subjected to a venous blood sample for the measurement of serum levels of ions, and they were clinically evaluated by means of the Harris Hip Score and Oxford Score were performed radiographic evaluation.

**Results** The median serum levels of ions measured were, respectively, 1.3 μg/l (0.1–9 μg/l) for chromium and 2.9 μg/l (0.85–13.8 μg/l) for cobalt. The average value of ions measured was 1.28 ± 0.52 μg/l for chromium and 4.26 ± 1.57 μg/l for cobalt. The HHS has increased from a mean pre-operative value of 39.3 ± 3.6 to a mean post-operative value of 93.2 ± 1.9. The Oxford hip score increased from a mean pre-operative value of 15.3 ± 2.7 to a mean post-operative value of 44.9 ± 1.2. There were no cases of pseudotumour or general complications from metallosis. In the radiographic evaluation were not highlighted in any case signs of osteolysis or loosening of the implant.

**Discussion** PTA-MOM allow excellent clinical and functional results but lately there have been reported increased serum levels of metal ions with different related complications. Unlike in our study we found an increase of moderate entity serum levels of Cr and Co without any consequences on local and systemic level.

**Conclusions** Therefore we believe that the total hip replacements with metal-on-metal (MOM-PTA) still represent a viable alternative for the treatment of coxarthrosis and that high increases in serum levels of metal ions reported in the literature are to be referred to defects in tribology of the materials of some specific installations.

### The ceramic–ceramic Maxera Cup large diameter heads: preliminary results

P. Caldora*[1], D. Lup[1], R. Guarracino[1], E. Bartoleschi[2]

[1]Ospedale S. Donato Arezzo e S. Margherita Cortona, ASL 8 Arezzo (Arezzo, IT);
[2]Clinica Ortopedica, Università degli Studi (Siena, IT)

**Introduction** The authors report the preliminary results of their experience with a new ceramic–ceramic coupling system with large diameter heads (Maxera Cup-Zimmer, Warsaw, Indiana, USA) in total hip arthroplasty. This system incorporates ceramic Biolox delta, which is characterized by low wear, high fracture strength and excellent biocompatibility, which make this system an appropriate choice for the treatment of younger and more active patients.

**Materials and methods** In our unit, from April 2011 to November 2013, 66 cups were implanted with mono-block ceramic insert Maxera Cup. In all cases we used a stem Fitmore. Forty patients were male and 26 were women, and their average age was 55 years (range 30–67 years). The pre-operative diagnosis was osteoarthritis in 51 cases and vascular aseptic necrosis in 15 cases. In all patients we performed a mini-posterolateral access. The clinical evaluation pre- and postoperative was performed using the Harris Hip Scores (HHS). Radiographic analysis postoperative was performed to assess the correct positioning of the components.

**Results** After a follow-up period of 16 months (range 21–3 months), clinical and radiographic results were good and satisfactory in all patients. We found an increase ROM compared to the previous standard plants. We observed no cases of dislocation, infection and early aseptic loosening. We have obtained excellent results in all post-operative radiographic cases.

**Discussion** The large diameter heads have a high degree of stability and allow a greater range of motion respecting conventional articulation. The coupling ceramic–ceramic is also characterized by low wear, so this system is particularly suitable for young and active patients and it is an appropriate alternative to metal–metal articulation.

**Conclusions** In our brief experience, the total hip prosthesis heads of large diameter ceramic–ceramic is a very reliable choice in young patients and a safe procedure with acceptable learning curve of the surgeon.

### Mid-long term experience with ceramic–ceramic biarticular cups

A. Olmeda*, L. Friso, S. Zanarella

Azienda Ospedaliera di Padova (Padua, IT)

**Introduction** Femoral neck fractures represent one of the toughest problems for our national health system. There are no proved differences in outcome between partial arthroplasty with bipolar head and total hip arthroplasty in elderly patients. The main cause of bipolar head failure is due to the polyethylene wear, that leads to a grip of the inner head with the external cup. Ceramic on ceramic bipolar head can avoid this complication, and allow to extend the life of the prosthesis quite as long as a THA, with a less invasive and cheaper surgery.

**Materials and methods** From 2005 to 2012, we performed partial hip replacement with ceramic–ceramic bipolar head in 406 consecutive patients. We implanted only uncemented press-fit stems SL Plus or Proxy Plus (Smith & Nephew), associated with CeramTec Duo bipolar heads. The mean age at the time of surgery was 81.5 (range 51–98), the timing of intervention was 36 h. Full load on the side of prosthesis was restored after 3 days from surgery. All of the patients were controlled with clinical and radiographical examination 6 weeks after the surgery. The minimum follow-up was 6 months (mean 28.5), 85 % had long-term revision with clinical examination or telephonic interview.

**Results** We have seen 2 peri-prosthetic fractures, 1 infection, 2 dislocations (all treated for pertrochanteric fracture), 2 asymptomatic fibrous stabilization. We have seen no cases of intolerance or ceramic rupture. At the last follow-up, the sinking of the stem was under 1.5 mm. Pain was absent or mild in 84.9 % of patients. Six months after the surgery, 82 % of patients returned to normal life and were able to walk individually, or using only one cane or one crouch.

**Discussion** With the new ceramic–ceramic bipolar head we haven't noticed new or unusual complications, the intra-operative assembling is easy and does not extend the time of surgery. The follow-up was free from accidents, and the new bipolar head seems reliable. Considering the problematic outcome of THA in elderly patients, and of resurfacing in younger ones, bipolar ceramic–ceramic cup may become an ultimate solution for partial hip arthroplasty in elderly patients, and a good solution for the other categories.

**Conclusions** On a mid-long term follow-up we can demonstrate that no particular complications have been observed. In our experience, outcome of bipolar ceramic–ceramic cup has no difference in comparison with the other kind of implants.

### *C47–HIP 2*

### Thigh pain after cementless total hip arthroplasty with the short stem Tri-Lock BPS, Gription coating

M. Ulivi*[1], L. Orlandini[1], V. Meroni[1], M. Bassi[1], V. Sansone[2]

[1]Istituto Ortopedico Galeazzi IRCCS (Milan, IT);
[2]Clinica Ortopedica dell'Università degli Studi di Milano, Istituto Ortopedico Galeazzi IRCCS (Milan, IT)

**Introduction** Thigh pain prevalence after cementless total hip arthroplasty varies in literature between 1.9 and 40.4 % according to stem type used. Incidence of thigh pain with the original Tri-Lock design varies from 2 to 9 %. The Tri-Lock short stem (Tri-Lock BPS) reduced in dimension and geometry in comparison to the original one, is available in Europe since 2010. Surface treatment employs Gription TM DePuy, a 3D matrix highly porous and rough. Aim of the study is to evaluate if the new stem is able to reduce thigh pain incidence.
**Materials and methods** Since March 2010 to date 197 (121 females, 76 males, mean age 69.65 years) consecutive patients have been enrolled. All patients received Tri-Lock BPS stem, Pinnacle cup, PE Marathon (DePuy) liner and ceramic ball head. Patients are prospectively followed-up at 6, 12 and 24 months post -intervention. Clinical evaluation is done with a visual analogue scale (VAS) including pain mapping, Harris Hip Score and standard radiology. For thigh pain is utilized the Barrack definition: only pain reported in the anterior aspect of the thigh and distal to the inguinal ligament is considered significant.
**Results** We report preliminary results concentrated on the primary objective of the study (thigh pain). To date are available evaluations at 6 months for 145 patients, at 12 months for 86 patients and finally at 24 months for 24 patients. Thigh pain at 6 months is present in 12 subjects with an incidence of 8.2 % (VAS mean value 3.25, range 2–6) at 12 months in 4 subjects with an incidence of 4.6 % (VAS mean value 3, range 2–3). Finally at 24 months on 24 subjects evaluated no thigh pain has been reported. No correlations of thigh pain with clinical or stem related variables could be detected.
**Discussion** With the increase in the prospective follow-up observations of patients it looks like that the light thigh pain present at short term in 4.6 % of subjects tends to subside and disappear at long term. This data are in contrast with the incidence of long term thigh pain reported for the stem in its original design which has been documented being up to 9 %.
**Conclusions** Gription porous coating offers and adequate primary fixation which is important in high functional demand patients.

### 7-year Kaplan–Meier survival of more than 1,000 consecutive total hip arthroplasties with the Trabecular Metal Modular Acetabular Shell

F. Magli*[1], A. Caramagno[1], F. Gotti[1], G. Li Bassi[1], D. Giglio[2]

[1]Poliambulanza (Brescia, IT);
[2]Ospedale S. Giovanni di Dio (Agrigento, IT)

**Introduction** The Trabecular Metal Modular Acetabular Shell is an elliptical-shaped cup made out of Tivanium alloy. The aim of this study is to present the 7 year Kaplan–Meier survival data of a consecutive series of total hip arthroplasty (THA) patients with the Trabecular Metal Modular Acetabular Hip Shell.
**Materials and methods** A total of 1,042 THA procedures in 985 patients, which were performed in one centre between September 2003 and October 2008 by two surgeons, were evaluated. Mean patient age at the time of surgery was 67.6 years (range 22–93 years). There were 637 female and 405 male patients. In all 985 patients a posterior–lateral approach was used. A Trabecular Metal Modular Acetabular Shell with a Longevity Highly Crosslinked Polyethylene Liner was used in all patients together with mainly SL-Plus Stems (Smith & Nephew Orthopaedics AG, Rotkreuz). Survival analysis for cup and stem were performed according to Kaplan–Meier using as end point revision due to aseptic loosening and revision for any reason.
**Results** Three cups had to be revised. One cup was revised due to septic loosening and 2 cups were revised due to peri-prosthetic fractures caused by trauma. The Kaplan–Meier survival rate of the cup for any reason was 99.7 % (95 % CI 99.3–100) at 7 years. Additionally 2 stems and 1 liner had to be revised during this follow-up period. The Kaplan–Meier survival with endpoint revision of any component for any reason was 99.0 % (95 % CI 98.1–100).
**Discussion** Survival rate at 7 years of the Trabecular Metal Modular Acetabular Shell is excellent, showing strong mid-term performance of the Trabecular Metal Modular Acetabular Shell.
**Conclusions** Even if mid-term results are excellent, long-term clinical and radiological data are needed in order to generate a complete and conclusive statement on the performance and safety of the Trabecular Metal Modular Acetabular Shell.

### Ceramic-on-metal hip prosthesis: study on 197 cases

C. Grinberg[1], M. Gison[1], G. Toro[2], G. Calabrò[1], A. Toro*[1]

[1]Ospedale “Martiri del Villa Malta” (Sarno, IT);
[2]Ospedale San Luca (Vallo della Lucania, IT)

**Introduction** Ceramic-on-metal hip prostheses have been produced searching the best compromise between the metal-on-metal, metal-on-polyethylene, ceramic-on-polyethylene debris and the ceramic-on-ceramic prosthesis squeaking. The aim of this study is to evaluate the clinical and functional outcome and the possible lysis due to debris.
**Materials and methods** From January 2008 to January 2013, we implanted 197 ceramic-on-metal hip prosthesis, 72 males 125 female. The mean age was 67, the hip replacement was performed by the same surgeon through the same access. Outcome evaluation through clinical and radiographic exams, and collected data were used to calculate Harris Hip Score. Moreover a randomized group performed CT and DEXA scan to evaluate prosthesis osteointegration and the possibility of lysis.
**Results** According to Harris Hip Score, follow-up data showed positive results in 95 % of cases; among these 74 % Excellent (HHS 100–91), 21 % Good (HHS 90–81) and 5 % Fairly good. CT and DEXA scan revealed a perfect osteointegration and absence of lysis in the Charley and Gruen zones in all the exams performed.
**Discussion** Coxarthrosis high incidence justifies the increase of hip replacement. Every year a million and half hip prosthesis are implanted, 300,000 in USA. Between European countries, Italy is at first place for hip replacements (about 100,000 intervention of all 700,000 European hip prosthesis by year). Ceramic-on-metal prosthesis could be favourable for many reasons: the possibility of use a 36 mm head with a 50 mm cup, while with other materials this is possible only with a 52 mm cup; absence of squeaking, frequent with ceramic-on-ceramic hip prosthesis; debris production similar to ceramic-on-ceramic hip prosthesis, as demonstrated in vivo and in vitro.
**Conclusions** Five years follow-up showed that the ceramic-on-metal prosthesis are a very good chance, because of the high percentage of positive results and the reliable osteointegration. However the high cost of materials induce to limit its indication to a very limited number of patients.

### Analysis of metal–metal: experience of the Livio Sciutto Foundation

G. Grappiolo*[1], G. Mazziotta[1], A. Camera[2], D. Ricci[1], A. Baldini[1], F. Traverso[1], M. Scardino[1], F. Astore[1]

[1]Istituto Clinico Humanitas (Rozzano, IT);
[2]Ospedale S. Corona (Pietra Ligure, IT)

**Introduction** The metal-on-metal total hip prosthesis is in great discussion. The warnings of National Registers and the literature led us to analyse our cases shared with the Livio Sciutto Foundation.
**Materials and methods** From March 2004 to February 2012 in Livio Sciutto Foundation Database (GAPII), 909 prostheses were implanted with metal-on-metal, 742 males (81.6 %) and 167 females (18.3 %) of mean age 52.7 (16–82). Were implanted 711 Durom cups and 198 Magnum cups. 6 implants were reviewed only for metallosis. We did a clinical evaluation with Harris hip score and radiographic evaluation. Were determined blood concentrations and urinary Cr and Co.

**Results** The patients had a good clinical post-operative HHS (HHS 94 % >90). There were no problems of impingement or dislocation. In one case there was evidence of radiographic signs of loosening for whom the cup was revised. At radiographic evaluation, prosthetic components were within the correct angles of orientation. The analysis of biological fluids showed a concentration of the metal ions only modestly increased.

**Discussion** Besides the initial enthusiasm which supported the adoption of increasingly frequent mating metal–metal, were highlighted progressively in recent years some concerns in relation to possible local and systemic reactions derived from the release of metal ions such as metallosis, pseudotumour and ALVAL (aseptic lymphocyte dominated vasculitis associated lesions). The international guide line has taken a position cautionary about recommending the suspension of metal–metal coupling in the conventional prosthesis in that area are still accepted specific indications. Despite fears, in our study we recorded a low incidence of complications, despite a relative increase of metals in biological fluids below the threshold values. We believe it is essential to obtain the most proper positioning of the prosthetic components to minimize wear and impingement. To obtain this, since 2010 we introduced the surgical technique of the femur first.

**Conclusions** The results obtained are very interesting for the low incidence of complications than the literature and we correlate this to the positioning of the prosthetic components. We observed the advantage of the technique of the femur first, but it remains the limit of a relatively short follow-up.

### *C48–HIP 3*

### Peri-prosthetic bone remodelling, PTA comparison with short stem and traditional stem

M. Giorgini*, L. Cavalli, A. Macera, R. Civinini, M. Innocenti

CTO, Università degli Studi di Firenze (Florence, IT)

**Introduction** The purpose of this study was to study the peri-prosthetic bone remodelling of a short stem to the comparison of a rod using a traditional method densitometric dual photon absorptiometry (DEXA).

**Materials and methods** Thirty patients were enrolled prospectively. In 15 cases was implanted a short stem GTS (Biomet) commitment to the prevailing metaphyseal was implanted in 15 cases a straight stem traditional PPF (Biomet). All cases were subjected to clinical evaluation (Harris Hip Score), radiographic and densitometric preoperative, immediate postoperative, 6 months and 1 year. The densitometric examination was performed with a Hologic device and peri-prosthetic remodelling was assessed by analyzing the rate of change between the two stems of the bone mineral density assessed in the 7 Gruen zones. For the short stem the Gruen zones have been adapted in a manner proportional to the length of the stem.

**Results** For 1 year after no cases were lost to follow-up, it was reviewed, and in no case were visible radiographically pathological peri-prosthetic bone. Regarding the densitometric evaluation areas zones 1 and 7, in the group of short stems there had been a decrease in the density bone significantly lower. Equally in the area 4 the increase in bone mass was significantly lower.

**Discussion** The use of short stems allows a less invasive in the diaphysis, saving the region trochanteric bone and a greater retention of the femoral neck, also the minor length of the rod should theoretically ensure better bone remodelling, decreasing the stress shielding. This advantage can be evaluated only in a quantitative manner with the use of densitometric methods and this has been the rationale of our study.

**Conclusions** The results show densitometers 1 year as a short stem with a reduced commitment diaphyseal allows a better peri-prosthetic bone remodelling, decreasing the distal distribution of loads and retaining most of the metaphyseal bone stock level.

### A long follow-up of the stem CLS: our experience

F.R. Evola*, L. Costarella, V. Pavone, G. Caff, L. Cannavò, S. Avondo, G. Sessa

Clinica Ortopedica dell'Università di Catania (Catania, IT)

**Introduction** The biological fixation showed greater long-term survival than cemented to the point of becoming the gold standard in hip arthroplasty. Stem CLS is one of the commonly used uncemented implants, with a proximal anchorage and a distal thinning that allows to not fill the femoral canal, reducing thigh pain. Purpose of this study was to evaluate retrospectively the CLS stem, in order to be able to evaluate complications related to the system and the long-term results.

**Materials and methods** From January of 1992 to January of 2012, 956 CLS stems were implanted in patients with degenerative or traumatic conditions of the hip. Of all these implants only 780 prostheses are available for a clinical and radiographic evaluation with a follow-up up to 20 years. The sample consisted of 412 women and 368 men, with an average age of 62 (range 48–79) at the time of the intervention. The indication for surgery was osteoarthritis in 643 patients, rheumatoid arthritis in 51, dysplasia in 34, avascular necrosis in 30, and trauma in 22. The patients were evaluated clinically before and regularly after intervention through the use of Harris Hip Score and through conventional radiography at 3, 6, 12 months, and each year thereafter.

**Results** The mean follow-up was 11.3 years (range 1–20). The clinical and radiographic follow-up performed at regular intervals over time have shown satisfactory results. From the clinical point of view it was found an increase in the average pre-operative HHS from 43.2 to 88.1. Radiographically, patients showed no signs of loosening or osteolysis or migration of plants, except for 15 plants that have been revised to aseptic loosening, 8 removed for infection, and 7 for peri-prosthetic fracture.

**Discussion** The survival of the cementless stem depends on the initial stability obtained during surgery and the biological properties of the implant surface, such as to promote osseointegration, which allows a gradual distribution of the loading forces from the proximal portion of the femur to the distal. The results demonstrate a high rate of long-term survival of this system.

**Conclusions** The CLS stem in several clinical studies has proven to be a safe and reliable system. The clinical and radiographic results of the CLS stem with a follow-up up to 20 years are encouraging, especially when the plant is well located and not undersized.

### Is it possible to resume sport activities after hip resurfacing? Retrospective clinical study on 82 cases

A. Moroni*[1], D. Leonetti[2], M.T. Miscione[3], R. Orsini[3], G. Micera[3]

[1]Dipartimento di Scienze per la Qualità della Vita, Università di Bologna (Bologna, IT);

[2]Ospedale Maggiore Bologna (Bologna, IT);
[3]Istituto Ortopedico Rizzoli, Università di Bologna (Bologna, IT)

**Introduction** An increasing number of young people undergo total hip replacement surgery and want to resume sports activities previously practiced. Several authors evaluated the possibility of sport resuming after a hip replacement, advising against high-impact sports such as football, basketball and baseball because there is a high risk of peri-prosthetic fractures, early loosening and dislocations. The resurfacing is a great alternative to traditional THR for the treatment of coxarthrosis and indicated in young and active patients. The aim of this work was to evaluate a series of 82 people treated by hip resurfacing arthroplasty that resumed high-impact sports activities.
**Materials and methods** We retrospectively reviewed 82 patients treated by hip resurfacing with a mean age of 45 ± 7.8. All patients were examined at 1, 3, 6 months, 1 year and then annually until the last follow-up (average 4.1 ± 1.5 years). At last follow-up score evaluation was repeated according to the Oxford Hip Score and the radiographs, racing resumed and the personal satisfaction of each patient were also evaluated.
**Results** The Oxford hip score increased from a mean pre-operative value of 24.4 ± 9.9 to a value of 46.6 ± 2.5 at the last follow-up. More than 90 % of patients resumed high-impact sports activities such as football and skiing 6 months after surgery. There were no failures of the systems.
**Discussion** The recovery in high-impact sports after total hip arthroplasty is not recommended. However, several authors reported good results with no increased risk of fracture after the resumption of high-impact sports activity in patients operated arthroplasty coating.
**Conclusions** The resurfacing is an excellent solution for the treatment of coxarthrosis in young patients and allow the resumption of high-impact sports 6 months after surgery without increased risk of prosthetic revision in the medium term.

### *C49–HIP 4*

### 3D volumetric evaluation of acetabular bone deficiency in case of cup loosening in total hip arthroplasty

M. Stamilla*[1], M. Benzid[2], S. Chemouny[2], F. Bonnel[2]

[1]Catania, IT;
[2]Montpellier, FR

**Introduction** Bone acetabular deficiency, in case of cup loosening in total hip arthroplasty, is generally evaluated with standard X-ray (Vives, 1988; D'Antonio, 1989; Paprosky, 1994). Past experience shows that the bone deficiency peri-operative appreciation is always more important than expected from imaging. The aim of our study is to evaluate first the preoperative real bone deficiency, furthermore to measure a cup loosening evolution.
**Materials and methods** We report a prospective series of 14 acetabular component loosening cases. There were 10 women and 4 men. The mean age at surgery was 68 years. The group of the patients was revised clinically and radiologically (standard X-ray and TAC). Tomodensitometric images were analyzed by means of specific program that evaluated 3D volumetric bone deficiency and bone peri-prosthetic density.
**Results** The bone deficiency has been divided into 3 stages: stage 1 the volume was between 10 and 20 $cm^3$; stage 2 the volume was between 20 and 40 $cm^3$; stage 3 the volume was more than 40 $cm^3$. In 2 cases, the follow-up at 6 months of an early acetabular component loosening showed evolution rate at 5 %. The volumes correspondence between stages observed by means standard X-ray and those measured by TAC did not show a significant correlation.
**Discussion** Comparing the two methods of volume measurement showed that the tomodensitometric was more accurate. The images preparation for segmentation was semiautomatic and required 30 min; the presence of prosthetic material did not induce artefacts. Results acquisition was automatic. 3D representation allowed evaluation, in the preoperative time, of acetabular areas most involved; also allowed to plan surgical acts and instruments to be used. The density analysis evaluation allowed us to determine the bone stock quality and the limit between healthy tissues, pathological tissues and the presence of cement that participated in the increase of bone deficiency.
**Conclusions** The automation of measurement techniques make it possible to reduce the analysis time. The accuracy of the measurements will be a complementary factor for the determination of the evaluation stages in case of bone deficiency as well as in the quantification of bone transplants replacements. This method is available for all joints.

### Revision arthroplasty in large acetabular bone defects: results and prognostic predictors

M. Mariconda, G. Costa, P. Recano*, G. Orabona, E. Aitanti, S. Cerbasi

Dipartimento Ortopedia, Università Federico II (Naples, IT)

**Introduction** The aim of this study was to assess results of different revision techniques for large acetabular defects and the identification of possible predictors of these results.
**Materials and methods** This retrospective study was carried out on 33 consecutive patients (22 females, 11 males) who underwent acetabular revision at our Institution for Paprosky grade II B and III (GIR 3 or 4) acetabular bone defects. The average age at surgery was 67.1 years (range 42–86 years). Burch-Schneider reinforcement cage and cemented cup was used in 25 cases, trabecular metal revision shell in 6 cases, and LOR cup in 2 cases. The mean time between primary and revision procedure was 9.1 years and the mean follow-up was 48 months (range 14–112). The Harris Hip Score (HHS) was obtained before surgery and at the latest follow-up control in all patients. Pain at follow-up was evaluated using a 10-cm Visual Analog Scale (VAS). In 27 patients a follow-up radiographic evaluation was also performed. Determinants of HHS score at follow-up were assessed by linear regression analysis, using age, sex, BMI, rate of co-morbidity (FCI), use of reinforcement ring, and time elapsed from the primary surgery as explanatory variables.
**Results** The average pre-operative and follow-up HHS score was 41 ± 16 and 79 ± 13, respectively ($p < 0.001$). The average pain reported by patients on the VAS scale at follow-up was 3.2 ± 1.7. The overall re-revision rate was of 18 % (6/33 patients). At the multivariate analysis the only negative determinant of the HHS at follow-up was FCI ($c = -5.3$, $p = 0.007$). Different surgical options were not associated with differences in the functional outcome. Three Burch-Schneider reinforcement cages showed slight signs of radiographic loosening. The use of trabecular metal components was associated, in most cases, to the presence of newly formed acetabular bone at the follow-up.
**Discussion** Newly formed bone on tantalum components was observed. Co-morbidity is a negative predictor of hip function. Our results are in keeping with previously published data.
**Conclusions** Positive clinical results were detected with both acetabular reinforcement cages and trabecular metal revision shells in severe acetabular defects.

### Treatment of the dysplastic hip with shortening osteotomy: surgical technique and up to 20-year follow-up

G. Grappiolo, F. Astore*, G. Mazziotta, F. Della Rocca, F. Traverso, A. Baldini, M. Scardino

Istituto Clinico Humanitas (Rozzano, IT)

**Introduction** Hip dysplasia in the presence of congenital dislocation is associated with angular deflection and torsion of the femur with increased complications after hip replacement. The purpose of this study is to evaluate the surgical technique of shortening osteotomy for the prosthetic treatment of severe hip dysplasia.
**Materials and methods** In this retrospective study, 1983 to 2010, we evaluated the results and complications of 52 patients (40 females, 12 males, mean age 55 ± 14 years) underwent implantation of total hip replacement (37 bilateral, 89 implants) for Crowe type IV congenital hip dysplasia. All prosthesis were implanted by placing the acetabular component in the anatomical centre of rotation in combination with an subtrochanteric femoral shortening osteotomy. The patients were re-evaluated with a mean follow-up of 8 years (2–20 years).
**Results** Only one patient reported clinical worsening, all the others have reported a significant clinical improvement with growth of average Harris Hip Score from 41.79 to 87.87. Were only used uncemented stems and is usually synthesized osteotomy with wire while in a few cases there has satisfied the press fit of the stem. Two dislocations in postoperative mobilization of the proximal stump treated with reduction and new synthesis. Four cases reviewed for non-optimal synthesis of the osteotomy. No damage to the sciatic nerve respecting a maximum elongation of 4 cm through the osteotomy. A case has been reviewed for the presence of heterotopic bone (grade 4). A case of soft tissue infection was treated with antibiotics. Two revisions of acetabular wear for 8 and 12 years after surgery.
**Discussion** The anatomical abnormalities associated with congenital hip dysplasia increase the complexity of a hip replacement. Subtrochanteric femoral shortening osteotomy ensures rapid bone consolidation, optimal management of muscle tendons in particular medium-gluteus and also the correct positioning of trochanter. We use rarely and in special cases (results of osteotomies) other techniques for the management of congenital hip dysplasia but the risks of nonunion of the greater trochanter are higher.
**Conclusions** The subtrochanteric femoral shortening osteotomy preserves the anatomy of the proximal femur, avoids the problems associated with the synthesis of the greater trochanter and facilitates a femoral reconstruction in relatively young patients.

### *C50–HIP 5*

### Does hip osteoarthritis have a protective effect on proximal femoral fractures? A retrospective study

F. Calderazzi*, F. Pogliacomi, G. Groppi, A. Ricotta, F. Ceccarelli

Azienda Ospedaliero-Universitaria, Ospedale Maggiore (Parma, IT)

**Introduction** To date, the inverse relationship between proximal femoral fractures and hip osteoarthritis remains controversial. Several studies distinguishing fractures of the femoral neck from those to the trochanter, however, do agree that femoral neck fractures rarely occur in patients with hip osteoarthritis, thus suggesting a protective effect of osteoarthritis limited to this type of fractures. We analysed the hypothesis that hip osteoarthritis would not protect from proximal femoral fractures, but only affect the location of the fracture in the proximal femur.
**Materials and methods** A retrospective study was performed by examining the radiographs of all patients who came to the emergency room of our Hospital for direct trauma in the trochanteric region from January 2010 to January 2011. The 190 patients analysed were divided into three groups according to the outcome of the trauma: femoral neck fracture (NF), trochanteric fracture (TF), and no fracture (NOF). Radiographic hip osteoarthritis was scored according to Kellgren and Lawrence (K/L). We separately compared the grade of osteoarthritis as (i) the presence of a proximal femoral fracture, (ii) the outcome of the trauma, and finally (iii) the location of the fracture by using Chi-squared test.
**Results** No relationship between the grade of hip osteoarthritis and the presence of a proximal femoral fracture was found. However, the grade of osteoarthritis was strongly related both to the outcome of the trauma ($p < 0.0001$) and to the location of the fracture ($p < 0.0001$). In fact, we found that patients with osteoarthritis of the hip presented a three-fold increase in trochanteric fractures than in those of the femoral neck.
**Discussion** Osteoarthritis does not protect against proximal femoral fractures, but strongly affects the location of the fracture in the proximal femur, increasing the possibility of a trochanteric location.
**Conclusions** The present study represents a starting point for future studies on the role played by HOA in processes underlying the fracture of the neck and of the inter-trochanteric region of the proximal end of femur.

### Cement augmentation method for inter-trochanteric fracture in osteoporotic elderly patients treated by intra-medullary nailing: a 6-year follow-up

T. Maluta*[1], C. Dall'Oca[1], B. Magnan[1], N. Leone, S. Mezzari

Azienda Ospedaliera Universitaria Verona (Verona, IT)

**Introduction** Trochanteric fractures are common in elderly people and their treatment has a rate of complications due to technical failure (cut out-head rotation).
**Materials and methods** We studied 62 patients (38 females, 24 males) with an average age of 84.36 years (range 80–95 years). They had an unstable trochanteric fracture, defined as fractures with three fragments or more, age more than 80 years and severe osteoporotic bone (1 or 2 Singh score). All patients were treated by Gamma Nail standard technique and augmentation was done with The Locker system (Tecres SpA) inserted through the cannulated cephalic screw at its apex. The evaluation is based on: operating time, early functional recovery using the modified Harris hip score, Rx TAD and sliding screw, mechanical and biological complications. During the follow-up we lost 16 patients. No one died because of surgical and anesthesiological complications or other causes due to the surgical procedure.
**Results** The HHS average score was 50.4 after 1 month post-operation, 56.21 after 3 months, 55.7 after 6 months, 59.45 after 12 months, 60.67 after 24 months, 61.55 after 36 months, 61.65 after 48 months, 62.55 after 60 months and 62.28 after 72 months. The average decrement of haemoglobin was 1.82. In 2 cases we reported a cement migration through the femoral head. No other complications (infection, cut out and femoral head necrosis) have been reported at the follow-up.
**Discussion** The review of recent literature highlights the need for augmentation as support of fractures due to osteoporosis, both for the upper and the lower limbs. The poor quality of the bone tissue would not provide a sufficient substrate in which the means of synthesis can be anchored stably leading to complications in topics.

**Conclusions** Literature review and the observations based on the reported results allow to conclude that the cement augmentation in severe osteoporotic bone could improve the mechanical stability of the implant, ensuring early functional recovery.

### The functional recovery of patients with fragility hip fracture after different rehabilitation procedures

C. Corradini, F. Boisio*, S. Pasqualotto, D. Tradati, C. Verdoia

Clinica Ortopedica dell'Università degli Studi di Milano (Milan, IT)

**Introduction** Because of the increasing of the survival in the general population and the increase of the hip fractures' worldwide incidence, the evaluation of the residual disability after surgery and rehabilitation is very important.

**Materials and methods** We considered 73 patients with medial or lateral hip fracture operated by the same surgeons. For each one we collected co-morbidities at the moment of the hospitalization, the age, complications occurred during the recovery, evaluation of pain with the VAS scale and disability with Barthel index during the visits, the analysis of the time needed to start walking and the typology of rehabilitation. A subanalysis of the patient treated in orthopaedic vs general rehabilitative department were conducted.

**Results** We found that patients with lateral fracture had a quicker recovery ($p < 0.05$), they started walking before the patients with medial fracture. Patients with medial fracture had slower recovery, more serious concomitant pathologies, and were older. The residual disability was significantly higher in patient with medial fracture ($p < 0.05$). Patients who received orthopaedical rehabilitation were found more functionally active than those treated in general rehabilitation.

**Discussion** Functional outcome after hip fracture is influenced by the type of fracture, co-morbidities and rehabilitation. It's very important that in future we will have the possibility to cure the patient with hip fracture, in a medical team coordinated by the orthopaedic surgeon and the physiatrist and composed by different figures for example as physiotherapist, cardiologist, geriatrician, nurses and others. The application of a specific rehabilitation determines also a better recovery.

**Conclusions** Medial and lateral hip fractures must be considered separately, not only surgically but also for rehabilitative period. The presence of co-morbidities suggest multidisciplinary approach possibly coordinated by orthopaedist.

### Atypical femoral fractures by bisphosphonates: retrospective study on over 250 femoral fractures and presentation of clinical cases

V. Bottai*, S. Giannotti, G. De Paola, G. Bugelli, E. Cei, G. Guido

Clinica Ortopedica e Traumatolgica, II Università di Pisa (Pisa, IT)

**Introduction** Bisphosphonates are the most highly used drugs in osteoporosis treatment especially in corticosteroid induced osteoporosis. However there have been increasing concerns about the potential risk of development of atypical femoral fractures on bisphosphonate therapy as defined by the Task Force of the American Society for Bone and Mineral Research. There are an increasing number of studies that highlight their possible relationship with the occurrence of atypical fractures defined by the Task Force of the American Society for Bone and Mineral Research.

**Materials and methods** The authors have reviewed over 250 Rx of femoral fractures in patients over 60 treated from January 2011 to February of 2013 at their centre. The fractures were divided by anatomical site. Among the subtrochanteric and diaphyseal fractures those with the largest radiographic criteria for the definition of atypical fractures were 3. To these must be added a case of atypical fracture hesitated in pseudoartrosis. Two fractures were associated with bisphosphonate taking and one was consequence of a melanoma. All cases in which there was association with bisphosphonates were subjected to radiographic evaluation of the contralateral femur, densitometric evaluation and biohumoral exams. It was also used SPECT-CT in the evaluation of the contralateral pre-fracture when present a contralateral cortical thickening.

**Results** In one of the three cases of fractures associated with bisphosphonates, healing occurred on schedule, another one is still under treatment and in the case of non-union the patient underwent re-intervention coming then to healing. In any case it has always been suspended the therapy with bisphosphonates and started the therapy with teriparatide. In two patients the femoral involvement was bilateral and in both cases SPECT-CT didn't show an increase of the uptake at the level of cortical thickening CT detected.

**Discussion** At present there is difficulty in defining the true incidence of atypical fractures associated with bisphosphonate as the studies are retrospective and there is no diagnostic code ICD-9 identification of this disease. However, our case study seems to indicate that the incidence of atypical fractures is higher than that reported in the literature when compared to fractures typical that occurred in the same anatomical site.

**Conclusions** Atypical fractures are rare, extremely underhanded and often unrecognized, may be bilateral, and seem to have a worse prognosis then typical fractures. Therefore it is necessary a careful assessment of patients treated by bisphosphonates, especially on long term treatments, for a correct diagnosis and an appropriate treatment setting.

### Proximal cut-out in the pertrochanteric fracture

R. Valentini*, L. Zandanel, M. Martino, G. Piovan, G. Fancellu

Clinica Ortopedica e Traumatologica, Università degli Studi di Trieste (Trieste, IT)

**Introduction** The worldwide incidence of fractures of the proximal femur is increasing. For pertrochanteric fractures the most frequent mechanical complication of the traditional systems of osteosynthesis is the cut-out of the proximal cephalic screw that occurs with an incidence of 4–12 %.

**Materials and methods** Our study took into account patients with cut-outs of the cephalic screw in previous surgery for fracture fixation with intramedullary nail pertrochanteric in Orthopaedic-Traumatology Clinic of Trieste from January 2003 to July 2011.

**Results** In this period there were 15 cases of cut-out (5 males and 10 females). The average age of the patient at the time of the second operation was 83 years (± 2.5) and the average time elapsed from the first osteosynthesis operation was 6.2 months. Specifically, out of 15 cases of cut-out, 4 patients were treated with PFN, 3 and 8 with gamma3 with IMHS CP. In 8 cases whose fracture radiographically had characteristics of successful consolidation, it was carried out by removing the cephalic screw, in cases where the fracture was not established, it was carried out by replacing the system in toto (4 cases), by replacing only the cephalic screw (2 cases) or performs partial unscrewing (1 case). The mean follow-up after revision surgery was 18 months.

**Discussion** All 8 patients with fracture-established after removal of the cephalic screw showed a fairly good functional recovery with no cases of refracture. The 4 patients undergoing removal of the nail and positioning of prostheses showed the best results from a functional point of view with the recovery of a normal gait. In the two cases where we have replaced the single cephalic screw, the results were not satisfactory, with persistent hip pain and severe functional limitation. The indication for early loading was placed in all patients.

**Conclusions** Some possible mechanical causes of cut-outs are still debated, but they are certainly related to the type of fracture, the stability of the reduction, the neck-shaft angle of the system and the positioning of the cephalic screw. The choice of surgery for revision must be mainly based on the time elapsed since the first surgery, which took place on whether or not consolidation of the fracture and the involvement of the acetabulum. A good positioning of the cephalic screw is extremely important.

### Unstable pertrochanteric fractures: lights and shadows

R. Valentini*, L. Zandanel, M. Martino, G. Piovan, G. Fancellu

Clinica Ortopedica e Traumatologica, Università degli Studi di Trieste (Trieste, IT)

**Introduction** The fractures of the trochanteric region of the femur represent a challenge to orthopaedic surgeons, among them those unstable added as problematic being biomechanically unfavourable. Theoretically the forces that tend to breaking down the fracture are better transmitted by an implant near the centre of the axis of the load so as to reduce the bending moments acting on the fracture.

**Materials and methods** In our department between 2008 and 2011, 396 patients were treated for pertrochanteric and subtrochanteric fractures of which 72 showed an unstable fracture. Complete data were available for 52 patients (42 treated with IMHS with or without added metal cerclages, 10 with screw and plate). The purpose of this retrospective study is to compare the clinical and radiographic outcomes between groups. The parameters evaluated were: operative time, time to radiographic consolidation, the period between the intervention and the granting of full load on the operated limb, the presence of complications, survival and duration of hospitalization.

**Results** The data of both groups are essentially super-imposable. The only significant difference was noted in the rate of failure, with a worse outcome in the group treated with screw plate ($p = 0.032$). In the osteosynthesis with IMHS and the cerclages we have noticed an increased operative time (about 40 min difference, $p = 0.0048$). A difference, though not significant ($p = 0.11$), was noted in the failure rate of the synthesis, with 10 cases in the group treated without the metallic cerclage (7 cut-out, 1 secondary failure and 2 post-operative diaphyseal fractures) and no cases in patients in whom has been necessary to add one or more cerclages.

**Discussion** From the results we can say that the two methods are similar with regard to operative time and the time required for the clinical and radiographic healing. The rate of a specific complication, the secondary scomposition of the fracture, has shown higher in patients treated with the screws and plate. At this point the IMHS seems to be more efficient, perhaps due to the fact of keeping intact the lateral cortex and acting with an effect of lateral buttress.

**Conclusions** There are not substantial differences between treatment with screw plate and intramedullary nailing, with or without the use of additional cerclages. The intramedullary nailing has shown, however, the best to avoid breakdowns secondary demonstrating greater stability of the synthesis. The addition of cerclage in more complex fractures seems to have a favourable effect on outcomes.

### *C51–HIP 6*

### Hip replacement in iliac Crowe 4 dislocations: surgical technique with relocation in paleocotile and femoral shortening osteotomy. Report on 35 cases

A. Dettoni*, M. Roselli, F. Amberti, A. Molinar Min

ASL TO2, Torino Nord, Ospedale Maria Vittoria (Turin, IT)

**Introduction** Aim of this study was the evaluation of technical surgical replacement with shortening osteotomy and repositioning it in paleocotile in iliac Crowe 4 dislocations.

**Materials and methods** Thirty-five cases treated by a single surgeon with the technique of repositioning in paleocotile and femoral shortening osteotomy. Description of pre-operative planning and tests and postoperative results.

**Results** The authors report good and excellent results with recovery of pain, length of leg and step pattern.

**Discussion** The authors give absolute chirurgical indication in unilateral dislocation with important hip pain an relative chirurgical indication in case of purely functional disorder. In bilateral dislocated hip, the indication is to be evaluated very carefully, because it may be preferable to refrain from surgery. The surgery should be performed on clinical symptoms and rarely acclaimed as a preventive measure. The technique need an accurate preoperative planning in positioning acetabular cup in paleocotile, using CT scan. If is needed a hip centre of rotation lowering higher than 3–4 cm is indicated subtrochanteric femoral shortening osteotomy and the use of special stems.

**Conclusions** The authors recommend this technique for this type of disorder. Excellent results and good restore of biomechanical parameter are achieved. We have to emphasize, however, that it is not simple technique and requires a skilled surgeon and an absolute respect of planning and surgical steps.

### Capsular management in arthroscopic treatment of FAI

F. Della Rocca*, D. Ricci, G. Grappiolo

IRCCS Istituto Clinico Humanitas (Milan, IT)

**Introduction** Recent anatomical and biomechanical studies confirm the importance of capsular ligaments in hip joint stability, even when bone containment is normal. In arthroscopic treatment of FAI more or less extensive capsulotomy and capsulectomy have been described, with or without capsulorrhaphy. Our aim was to check with the aid of clinical tests for the presence of instability after anterior capsular suture.

**Materials and methods** We clinically evaluated 23 sport practicing patients (16 males, 7 females), mean age 38 years (18–55), with mean follow-up of 14 months (12–19) undergoing hip arthroscopy for unilateral symptomatic FAI (CAM or mixed), between March and November 2011, when a capsular suture was performed after interportal or T capsulotomy (north–south and east–west sutures). Preoperatively patients showed no signs of generalized ligamentous laxity, no stiffness, capsulitis or synovitis. X-ray confirmed normal containment or minimal bone deficiency (Wiberg CEA >22°, Lequesne VCAA >20°, Tonnis AI <12°, stability index SI >16°) and Tonnis grade $\leq 1$. Post-operatively patients began an immediate flexion–extension (0°–90°) to prevent capsulo-labral adhesions, no external rotation and extension >0° for 2 weeks.

**Results** At follow-up, mHHS showed an average increase from 61 to 85 and SF12 from 66.35 to 87.42. The anterior apprehension test was negative in all patients. The log roll test for pain was always negative, with 2 cases of reduced elastic recoil. The symmetry of external rotation with extended limb showed 2 cases with an increase of 10° and 20°, and 1 case with loss of 10°. We did not have major complications.
**Discussion** Good clinical results and very rare cases of dislocation (usually in dysplasia) without capsulorrhaphy have been reported in the literature after arthroscopy for FAI. Atraumatic instability is a little recognized entity, often seen in sport practicing populations, not an infrequent reason for arthroscopic revision. According to some authors the capsular suture gives greater possibility to maintain or restore stability, and can also reduce the risk of joint stiffness and adhesions, allowing early external rotation. The specific tests for joint stability we have carried out, are in accordance with these hypotheses.
**Conclusions** We believe that a complete as possible routine suture of the capsule, is also helpful for patients without significant radiographic and clinical instability, or stiffness, in recovering anatomical alignment of the ligaments. It reduces the possibility of iatrogenic macro and microinstability, and can speed up the recovery process after hip arthroscopy. However, the learning curve and longer surgical time must be taken into account.

### Natural course of early radiological signs of femoro-acetabular impingement in an asymptomatic population

C. D’Arrigo, F. Alberti*, A. Speranza, R. Alonzo, S. De Sanctis, B. Maestri, A. Ferretti

Centro di Traumatologia dello Sport Kirk Kilgour, Azienda Ospedaliera Sant’Andrea, Università degli Studi di Roma “La Sapienza” (Rome, IT)

**Introduction** Femoro-acetabular impingement (FAI) has been recognized as a risk factor for development of osteoarthritis (OA) of the hip. Early surgical treatment has been advocated as a valuable method to prevent development of FAI and subsequent OA. The purpose of this study is to evaluate the radiological and clinical outcome of randomly early detected radiological signs predisposing towards FAI.
**Materials and methods** All 2,360 computed tomography (CT) scans executed in the Emergency Department of our Institution in the year 2006 were examined. Criteria of inclusion were: age (ranging from 20 to 40 years of age), ability of the scan to comprehensively evaluate hips. Patients with history of previous hip problems or trauma were excluded. The presence of the following bone abnormalities predisposing towards FAI were investigated: centre edge angle, acetabular version angle, crossover sign. 44 patients (88 hip joints) were included in this retrospective study. At a minimum follow-up of 5 years 42 patients were reviewed and clinically evaluated using Hip Outcome Score. Moreover 19 patients repeated radiological examination.
**Results** In 34 out of 88 hip joints at least one radiological sign predisposing towards FAI was initially identified. At follow-up no patients developed symptoms. In no cases hip joints with one or two radiological signs at initial examination were found to have an increased number of signs at follow-up.
**Discussion** This is the first study, according to our knowledge, that investigate radiological signs predisposing towards FAI and development of clinical symptoms. This study shows that number and severity of occasionally detected early radiological signs of FAI don’t increase and that patients remain asymptomatic at medium term follow-up.
**Conclusions** On the basis of this study the actual role of early detected radiological factors predisposing towards FAI and subsequently towards OA remains unclear in asymptomatic patients, as well as time of development of FAI as a distinct clinical entity.

### Capsule management in the arthroscopic treatment of femoro-acetabular impingement

L. Pierannunzii*, A. Guarino

Istituto Ortopedico Gaetano Pini (Milan, IT)

**Introduction** Nowadays hip arthroscopy is considered the gold standard to address femoro-acetabular impingement (FAI). Several techniques were developed, but no significant evidence is provided so far to help the surgeons’ choice. Particularly, capsule management is extremely variable during femoral osteochondroplasty, resulting in capsulectomy, capsulotomy (sutured or not) or complete capsule preservation. The present study compares the two extreme options: capsulectomy *versus* capsule preservation.
**Materials and methods** Twenty cam-type FAI cases were prospectively evaluated: 10 cases underwent anterior capsulectomy (with central compartment first approach) and were included in group A, while 10 cases had the capsule preserved (with peripheral compartment first approach and mild capsular release) and were included in group B. Operative times, complications, VAS, mHHS were compared both at discharge and at subsequent visits for a 6 month follow-up.
**Results** Operative times were similar as overall duration, but traction time was significantly shorter in group B. As for the scores, 48 h VAS, 1 month mHHS and 3 month mHHS were better in group B ($p < 0.05$). As for the complications, one patient of group A had a transient sciatic nerve sensory damage, while no complications occurred in group B. In two cases the capsular release produced an accidental capsular perforation, but this did not result in any changes of the surgical technique.
**Discussion** The 6 month scores did not show significant differences between the two groups, however group B patients had better short-term results. Moreover the shortened traction time might lead to a lower incidence of traction-related complications. Although both sample size and follow-up are limited, this is the only comparative trial available so far.
**Conclusions** Even though functional midterm results are similar, capsule-sparing femur osteochondroplasty seems to be better tolerated than the capsulectomy technique in the early postoperative time.

### CT scan study of 32 patients with idiopathic femoro-acetabular impingement: etiopathogenetic assumptions

T. Maluta*, C. Dall’Oca, T. Romeo, B. Magnan

Azienda Ospedaliera Universitaria Integrata (Verona, IT)

**Introduction** The femoro-acetabular impingement (FAI) is currently recognized as one of the most important pathophysiological mechanisms underlying primary osteoarthritis of the hip. In recent years, scientific attention has been intensely focused on the analysis and the study of this pathophysiological process and subjects presenting clinical and radiological features of FAI with a history of previous pathologies undetected for joint hip joint.
**Materials and methods** In our clinic we studied 32 patients with FAI (9 CAM, PINCER 7 and 16 mixed) suffering from hip pain. All of them were waiting for hip arthroscopy. We analyzed, using CT scan with 3D reconstructions, the femoral and acetabular ante-retroversion. Crossing the data obtained through the surveys dynamic gait analysis performed on 28 patients under the age of 65 years with a full-blown picture of osteoarthritis waiting for prosthetic replacement surgery, we studied the biomechanical and pathophysiological characteristics of the idiopathic FAI.

**Results** The results of our study have shown a close correlation between the femoral retroversion and CAM impingement and between acetabular retroversion and PINCER impingement.
**Discussion** Recent biomechanical studies, performed on healthy hip joints, described a functional retroversion of the femoral neck in spite of a physiological anti-version. These evidences seem to support our hypothesis on the onset of the FAI. A functional retroversion, coupled with a pathological retroversion of the femoral neck, although partially offset by a gait in external rotation, as evidenced by our studies of gait analysis, will probably lead to the formation of a femoro-acetabular impingement.
**Conclusions** Analyzing the biomechanics of the hip joint affected by FAI, we met many limitations: the CT scan is a static form of examination and the number of our patients was too small. The results obtained from this study are encouraging but still incomplete. They underline the importance of assessing fully the lower limb in the approach to FAI.

### *C52–PROSTHESES-KNEE I*

### Knee arthroplasty: hospital length of stay and co-morbidity

G. Ciapini*, V. Cervi, L. Dolfi, P. Parchi, M. Lisanti

Ortopedia e Traumatologia I Cisanello (Pisa, IT)

**Introduction** Total knee replacement (TKR) is one of the most common orthopaedic procedures performed in Italy with an increase practice of 11 % from 2001 to 2009. The current need is therefore to reduce hospital length of stay in order to meet the increased demand and lower the costs of hospitalization.
**Materials and methods** Post-operative outcomes of patients who underwent TKR at the 1st Orthopaedic Division of Pisa University between April 2008 and September 2009 were evaluated retrospectively. The sample consisted of 100 patients with a mean age of 70 years. All patients diagnosed with knee osteoarthritis undertaking a primary TKR were included in the study. Referring to fast-track protocols we have considered: age, sex, BMI, co-morbidities and ASA score, number of blood units transfused, post-operative analgesia protocol, physiotherapy protocol, day of the week when surgical procedure was performed, surgical access route. Each parameter was correlated with length of stay in order to identify whether, and how it would influence the post-operative period. The physiotherapy protocol for all patients began the first post-operative day. Post-operative analgesia was based on the use of ketorolac and morphine in elastomeric pump for 48 h, with paracetamol as rescue treatment. The surgical route was Midvastus in all patients and all surgical procedures were performed by the same operator.
**Results** In our series (2008–2009), the average length of hospital stay was 9.25 days. Among analyzed factors, the following resulted to be predictive of length of stay ($p < 0.05$): ASA score $\geq$3 (+1.5 days), BMI $\geq$ 30 (+2.3 days), need for blood transfusion $\geq$2 (+3.26 days), age $\geq$75 (+2.12 days).
**Discussion** Bleeding was shown to be the most important amendable variable that if controlled could lead to a significant reduction in length of stay. Obesity and consequently reduced mobilization could be addressed with a more aggressive and early rehabilitation protocol. ASA and age which had a significant impact with length of stay are not modifiable.
**Conclusions** The identification of the variables influencing length of stay is important to reduce costs and deliver a better care for patients. In view of the results of this audit, we have monitored more closely bleeding by introducing the use of the tranexamic acid and promoting early mobilization in obese patients, in agreement with the most recent literature. The preliminary results obtained, following this new approach, appear to be satisfactory (TKR mean hospital length of stay reduced of 2 days).

### Intra-medullary guide for distal femoral cutting: the weak point of standard instrumentation in TKA

A. Ferretti, G. Bolle*, F. Conteduca, D. Mazza, L. Valeo, J. Conteduca, C. Iorio, P. di Sette, F. Alberti, A. Redler, R. Iorio

Ospedale Sant'Andrea (Rome, IT)

**Introduction** Previous studies documented that computer-assisted surgery (CAS) total knee arthroplasty (TKA) provide better alignment as compared with standard technique. However few studies investigated whether the greater accuracy provided by CAS is specifically related to the femoral or tibial resections or both. Our hypothesis is that in standard technique errors can occur due to the rougher accuracy of femoral intra-medullary guide as compared with extra-medullary tibial one.
**Materials and methods** Between June 2008 and December 2011, 279 patients with primary gonarthrosis were prospectively enrolled for TKA. Patients were randomly divided into two groups: group A, 124 patients operated with conventional surgery; group B, 155 patients operated with computer assisted surgery. Post-operative leg alignment was measured on long-leg weight-bearing X-rays in full extension in all patients (mean 6 months, range 3–8). Radiographic measurements included the following parameters: angle A, mechanical leg axis in the coronal plane, planned value is defined as 0° ± 3°; angle B, tibial component in the coronal plane, is defined as 90° ± 2°, with reference to the coronal tibial mechanical axis; angle C, femoral component in the coronal plane, planned value is defined as 90° ± 2°. Mean values and standard deviations of radiographic measurements results were analyzed in each group via standard Student's $t$ test. The critical level of significance was set at $p < 0.05$.
**Results** Angle A was 2.6° ± 1.2° in the group A and 1.3° ± 1.5° in the group B. This difference was statistically significant ($p < 0.001$). Angle B was 90.2° ± 1.5° in the group A and 89.7° ± 1.8° in the group B. This difference was not statistically significant ($p > 0.05$). Angle C was 92.0° ± 2.1° in the group A and 90.3° ± 1.9° in the group B. This difference was statistically significant ($p < 0.001$).
**Discussion** One of the parameters we evaluated, coronal femoral component alignment, showed a statistically better accuracy using navigation compared with the conventional technique. The outcome was not surprising because the CAS system's algorithm can allow for a more accurate detecting the centre of the femoral head. Femoral bow, as well as the variability in canal diameter, the point of entry of the rod and the mismatch in the diameter of the intra-medullary guide and the canal diameter, all influence the distal femoral cut in the conventional technique resulting in a quite inaccurate position of the femoral component.
**Conclusions** This study using CAS suggest that the intra-medullary device for cutting guide positioning of the femoral component is the weak point of the standard/manual instrumentation.

### Radiographic analysis of an accelerometer-based system for the tibial resection in total knee arthroplasty

A. Ferretti, D. Mazza*, F. Conteduca, A. Redler, G. Bolle, J. Conteduca, P. di Sette, C. Iorio, F. Alberti, L. Valeo, R. Iorio

Ospedale Sant'Andrea (Rome, IT)

**Introduction** In total knee arthroplasty extramedullary tibial guides could not to be as accurate as requested in obtaining proper alignment perpendicular to the mechanical axis. The aim of this study was to determine the accuracy of an accelerometer-based system (KneeAlign 2; OrthAlign Inc, Aliso Viejo, California) as evaluated by post-op X-rays analysis.

**Materials and methods** Between March 2012 and May 2012 fifty consecutive patients with primary gonarthrosis were selected for unilateral total knee arthroplasty (TKA) using a handheld surgical navigation system to perform the tibial resection. Post-operatively, standing antero-posterior hip-to-ankle radiographs and lateral knee-to-ankle radiographs were performed to determine the varus/valgus alignment and the posterior slope of the tibial components relative to the mechanical axis in both the coronal and sagittal planes. The difference between the intra-operative reading of the tibial varus/valgus alignment and posterior slope provided by the system was compared to the radiographic measurements obtained postoperatively for each respective case. Differences were analysed via standard Student's *t* test. The critical level of significance was set at $p < 0.05$.

**Results** Intra-operatively, the average reading provided by the system with regard to varus/valgus alignment before performing the tibial resection was $0.3° \pm 0.3°$ relative to the mechanical axis and $5.4° \pm 0.9°$ in the sagittal plane. The average tibial component alignment postoperatively in the knees with was $0.6° \pm 0.3°$ in the coronal plane ($p = 0.07$) and $4.7° \pm 0.9°$ in the sagittal plane ($p = 0.07$). In no case a difference >2° from the planned resection was detected in both coronal and sagittal plane.

**Discussion** The system combine the accuracy of computer-assisted surgery systems with the ease of use and familiarity of conventional instrument. There are several advantages of portable navigation compared with large-console systems. Accelerometer-based, portable navigation avoids the use of additional pin sites and reference arrays in the femur and tibia, does not require a large computer with an infrared camera, eliminates intra-operative line of sight issues. Lastly, the system provides a degree of familiarity to surgeons accustomed to using conventional alignment guides because the tibial jig is similar to an EM alignment guide, avoiding any substantial increase in surgical time.

**Conclusions** This study demonstrates that the system is highly accurate for performing the tibial resection in TKA, as 100 % of the tibial components were aligned within 2° of perpendicular to the tibial mechanical axis.

### Videofluoroscopic accuracy assessment of two different patient-specific instrumentation systems for total knee arthroplasty

A. Timoncini*[1], A. Ensini[1], F. Cenni[2], S. Tamarri[2], A. Feliciangeli[1], S. Giannini[1]

[1]Istituto Ortopedico Rizzoli, Clinica I (Bologna, IT);
[2]Istituto Ortopedico Rizzoli, Laboratorio Analisi del Movimento (Bologna, IT)

**Introduction** The assessment of component positions and alignments in total knee arthroplasty (TKA) is carried out traditionally by radiography or CT, the latter being the only one which can also measure the alignment in the transverse plane. In recent years, videofluoroscopic analysis has been used to measure the three-dimensional relative movement between prosthetic components. This technique could allow matching computer tridimensional models of the prosthetic components also with the prepared bones, for the final component-to-bone alignment to be derived. The purpose of this study is to assess with an original videofluoroscopic analysis the accuracy of two different patient-specific instrumentation (PSI) systems, for the first time in terms of position and alignment discrepancies of the final prosthetic components with respect to the preoperative planning.

**Materials and methods** From January 2011 to April 2012, 44 knees affected by tricompartmental arthritis were replaced: 23 using the CT-based MyKnee system (group A), and 21 the MRI/X-ray-based Visionaire system (group B). At 5th post-operative day, coronal and sagittal fluoroscopic acquisitions of the replaced knee were performed. Each single fluoroscopic scan was processed by the Kneetrack software, in order to determine the antero-posterior, medio-lateral, and distal-proximal positions and the coronal, sagittal and transverse alignments of the prosthetic components with respect to the bone. Discrepancies larger than 3° were considered as outliers. PSI systems were statistically compared.

**Results** In both groups, the largest mean positions discrepancy in each of the three directions was 3.1 mm. Group A showed significantly larger ($p < 0.05$) discrepancies in the anterior-posterior and the medio-lateral direction for the tibia positioning. All mean alignment discrepancies were not larger than 1.9°. Group A showed the largest percentage of outliers, 20 %, for the femoral in the sagittal plane.

**Discussion** The videofluoroscopic analysis can be a viable tool to assess the three-dimensional positions and alignments of prosthetic components. This analysis showed a good accuracy of both PSI systems, especially in the coronal plane.

**Conclusions** The videofluoroscopic analysis showed that both PSI systems had similar accuracy in prosthetic components positions and alignments.

### Functional evaluation of the post-operative tibio- and patello-femoral kinematics in total knee replacement with fluoroscopy, gait analysis and electromyography

A. Feliciangeli*, A. Ensini, F. Cenni, C. Belvedere, A. Leardini, F. Fusai, S. Giannini

[1]Istituto Ortopedico Rizzoli (Bologna, IT)

**Introduction** Total knee replacement (TKR) is an effective surgical treatment, although patients do not fully recover the original knee kinematics and muscle activity. Unlike the tibio-femoral joint (TFJ), the patello-femoral joint (PFJ) has been little studied, although it is essential in the extensor mechanism. An in-depth kinematic analysis could then be useful for a complete evaluation of patients operated of TKR. This study reports an evaluation by gait analysis (GA) and electromyography, along with careful monitoring of movement of the prosthetic components (including patella), via three-dimensional video-fluoroscopic analysis (FA).

**Materials and methods** Six patients operated of TKR posterior-stabilized were analyzed 6 months after the surgery. GA was carried out during walking, ascent of the stairs, lift-and-sitting from chair, flexion and extension against gravity using a 8 camera system, synchronized with electromyography. At the same time FA was carried out for the same motor tasks. Three tantalum spheres, inserted in the polyethylene component during surgery were used to track the movement of the patella relative to the femur. All patients were evaluated with pre- and post IKS.

**Results** Gait analysis showed similar kinematic trends in both limbs. During flexion-against-gravity and the extension-against-gravity from the FA, the rotations of the PFJ were respectively: 64.4° and 64.4° in the sagittal-plane (patellar flexion), 6.7° and 6.8° in frontal-plane (patellar rotation), 6.3° and 8.5° in the transversal-plane (patellar tilt). Greater adduction and abduction of the TFJ occurred respectively in patients with a larger lateral and medial patellar rotation. Patients with lateral patellar rotation showed a prolonged activity of the extensor mechanism. In contrast, a more natural kinematic performance together with a normal muscle activity, occurred in patients with medial rotation of the patella in flexion.

**Discussion** The present study provides a comprehensive view of the functional recovery after TKR, through the combination of GA and FA and the analysis of both the TFJ and the PFJ, the latter monitored for the first time with an original technique. Traditional measures have been enhanced by new observations on the behaviour of the PFJ post-operatively in relation to rotations during patellar flexion. Patients with abnormal rotations of the PFJ show abnormal kinematics.
**Conclusions** When a sufficient number of cases will be reached, the functional assessment post-operatively with these accurate techniques will allow to evaluate PFJ and TFJ kinematic abnormalities in relation to patellar rotations and any relationship with clinical results.

### Does total knee arthroplasty modify flexion axis of the knee? An *in vivo* study

D. Bruni*, F. Iacono, B. Sharma, I. Akkawi, M. Lo Presti, G. Raspugli, S. Zaffagnini, M. Marcacci

Istituto Ortopedico Rizzoli (Bologna, IT)

**Introduction** The optimal reference for rotational positioning of femoral component in total knee replacement (TKR) is debated. Navigation has been suggested for intra-op acquisition of patient's specific kinematics and functional flexion axis (FFA). To prospectively investigate whether pre-operative FFA in patients with osteoarthritis (OA) and varus alignment changes after TKR and whether a correlation exists between post-operative FFA and pre-operative alignment.
**Materials and methods** A navigated TKR was performed in 108 patients using a specific software to acquire passive joint kinematics before and after TKR. The knee was cycled through three passive range of motions (PROM), from 0° to 120°. FFA was computed using the mean helical axis algorithm. The angle between FFA and surgical TEA was determined on frontal (αf) and axial (αa) plane. The pre- and post-op hip-knee-ankle angle (HKA) was determined.
**Results** Post-operative FFA was different from pre-op FFA only on frontal plane. No significant difference was found on axial plane. No correlation was found between HKA-pre and αA-pre. A significant correlation was found between HKA-pre and αF-pre.
**Conclusions** TKR modifies FFA only on frontal plane. No difference was found on axial plane. Pre-op FFA is in a more varus position respect to TEA. The position of FFA on frontal plane is dependent on limb alignment. TKR modifies the position of FFA only on frontal plane. The position of FFA on axial plane is not dependent on the amount of varus deformity and is not influenced by TKR.

### Self-alignment technique of extramedullary tibial guide may improve the accuracy of bone cut in total knee arthroplasty

G. Cinotti, P. Sessa, F. Ripani, A. Della Rocca, W. Salustri, F. Gabriele

Orthopaedic Department, University La Sapienza (Rome, IT)

**Introduction** Several investigations have shown that a proper implant alignment may reduce the risk of aseptic loosening and improve the longevity of the implant in total knee arthroplasty (TKA). However, implant misalignment >3° were found in 2–40 % of patients when standard TKA are performed. In the present investigation we assessed the accuracy in tibial cut alignment using a new surgical technique for the orientation of extramedullary guide.
**Materials and methods** Eighty patients undergoing standard TKA were analysed prospectively. In 40 patients (group A) the orientation of extramedullary guide was aligned proximally on the medial 1/3 of the tibial tuberosity and distally on a point located 5–10 mm medially to centre of intermalleolar distance. In the remaining 40 patients (group B), the extramedullary guide was aligned, proximally, as in group A and distally using a self-alignment technique. This included that the extramedullary guide was left in the middle of the perimalleolar clamp without align it to any definite anatomical landmark of the ankle joint. The coronal alignment of the tibial cut was assessed intra-operatively using navigation and postoperatively on standing radiographs.
**Results** Intra-operative evaluation showed a varus-valgus cut >3° in 11 cases (27.5 %) in group A and in 3 cases (7.5 %) in group B ($p = 0.03$). Of the patients showing misalignment, a bone cut in varus was found in 10 subjects in group A and in 1 of those in group. A misalignment >4° was found in 2 patients (5 %) in group A and in no patient in group B. Percentage of outliers on post-operative radiographs were similar to intra-operative evaluation, being 12 and 2 cases in group A and B, respectively.
**Discussion** Previous studies showed that tibial torsion may play a role in reducing the accuracy of extramedullary instrumentations either because tibial torsion was found to affect the distal alignment of extramedullary guide and because tibial torsion showed a wide variability among patients, which is difficult to recognize during surgery. This potential source of error in the alignment of extramedullary guide could be reduced by using a surgical technique which is not affected by tibial torsion, as the self-alignment technique used in this study.
**Conclusions** Using standard instrumentation for TKA, the percentage of outliers in tibial cut orientation may be reduced using a surgical technique in which the extramedullary instrumentation is aligned to the proximal tibia only.

### *C53–PROSTHESES-KNEE II*

### Mechanical axis versus functional axis in total knee arthroplasty: preliminary results

A. Timoncini*[1], A. Ensini[1], A. Leardini[2], P. Barbadoro[1], F. Fusai[1], M. d'Amato[1], S. Giannini[1]

[1]Clinica I, Istituto Ortopedico Rizzoli, (Bologna, IT);
[2]Laboratorio Analisi del Movimento, Istituto Ortopedico Rizzoli, (Bologna, IT)

**Introduction** A post-operative mechanical axis (MA) within 3° of varus or valgus deformity seems not necessarily related to better long term clinical outcomes. On the other hand, some recent studies suggest that the alignment of femoral and tibial components to the functional axis (FA), i.e. the axis of the cylinder best fitting the two posterior condyles of the knee, can improve the clinical outcomes and the implant longevity because of the better ligament balancing. The purpose of this study is to compare three groups of patients implanted with total knee arthroplasty (TKA): two according to MA, using Conventional Instrumentation (CI) or Patient-Specific Instrumentation (PSI), one according to FA using PSI. The assessment was based on clinical and radiological measurements.
**Materials and methods** Eighteen patients were implanted with Triathlon CR TKA (Stryker, USA): 9 according to MA, 6 using CI (group A) and 3 using PSI (group B) respectively, and 9 according to FA with PSI (group C). All PSI patients underwent pre-operative MRI scans of the hip, knee and ankle joints according to the Otismed protocol for the creation of pre-operative web-based planning and patient-matched cutting blocks. In group A and B, the prosthetic components were aligned to 0° MA in the coronal plane, in group C to the patient-specific FA. Clinical evaluation was performed with IKS pre-operatively, at 45 days, 3 and 6 months post-operatively. Final coronal component alignments and mechanical axis were measured on weight-bearing X-ray of the lower limb at 45 days post-operatively. X-ray measured alignments were compared with the corresponding planned alignments. Discrepancies larger than 3° are considered as outliers.
**Results** Group A showed a mean preoperative IKS scores of 46 ± 12 and at 6 months follow-up of 78 ± 19; corresponding values in group B were 49 ± 19 and 78 ± 23, in group C 59 ± 11 and 91 ± 12. Group A showed the highest percentage of outliers, 20 %, in the prosthetic components alignment.
**Discussion** Prosthetic component alignments to the FA showed the best clinical results at 6 months post-operative. PSI group showed more accurate coronal component alignments and post-operative mechanical axis with respect to the CI groups.
**Conclusions** FA seems to be a viable alternative to the MA for the coronal alignment of the prosthetic components in TKA.

### TKA patient matched: a clinical and X-rays alignment evaluation of 150 cases

A. Colombelli*, Y. Rizqallah, E. Lupetti, A. Belluati

Santa Maria delle Croci (Ravenna, IT)

**Introduction** The patient matched technology uses MRI's information to realize personalized cutting blocks for primary TKA. These blocks determine the thickness and the angles of cuts as well as the rotations of the components, reducing the possibility of malposition.
**Materials and methods** The report is based on three parallel studies: retrospective study of 150 Visionaire TKA (3 years follow-up), case–control study of two homogeneous groups of 30 patients treated with Visionaire technology and traditional technique, and radiographic study of the alignment of the prosthesis in the entire population. Clinical studies are based on the interpretation of KSS and KOOS questionnaires administered at various stages of post-operative: in the first case, the scores were related to those published internationally. Radiographic study takes into account the femoral–tibial axis, coronal, and sagittal alignment-slope of the tibial component.
**Results** The KOOS show values 34.8 pre-operative, 71.4 at 1 year, 79.3 at 2 years and 81.3 at 3 years. The KS score and function pre-operative 42–46.5 have grown 74.7–82.9 at 1 year, 76.3–85.6 at 2 years and 82.5–86.6 at 3 years. The two groups showed homogeneous case–control values, respectively KOOS 37.6–36.8 pre-operative, 78.6–76.8 at 1 year and 84.5–84.6 at 2 years ($p \geq 0.1$) and values KS score and function pre-operative [43.5–45.5]–[44.5–46], at 1 year [78.4–77.9]–[78–79.4] and at 2 years [82.4–84.6]–[81.9–86.1] ($p \geq 0.1$). The post-operative tibio-femoral angle's average was 4.9° valgus, tibial coronal alignment was 90.3°, while the slope of 3.3°: respectively 99.3, 98.7 and 99.7 % of patients is part of an ideal group ± 2.5° with respect to the desired values.
**Discussion** The clinical results obtained did not show statistical significance ($p \geq 0.1$), for which there is no scientific evidence that the system harbours a clinical benefit in the short to medium term to the patient. Deserves a special mention discussion on the radiographic findings, which instead show a significant reduction in the percentage of misalignment.
**Conclusions** The patient-matched system is an effective, reproducible, user friendly. The surgical times are lower and the instrumentation is simplified. The clinical results in the short to medium term are comparable to the traditional method, but they deserve a long term appreciation, according to the significant improvement in axial alignment of the prosthetic components.

### Reliability of computer assisted measured resection techniques in TKA

R. Mugnai*[1], V. Digennaro[1], A. Ensini[2], M. Gialdini[1], G. Grandi[1], F. Catani[1]

[1]Azienda Ospedaliero-Universitaria Policlinico di Modena (Modena, IT);
[2]Clinica Ortopedica II, Istituto Ortopedico Rizzoli (Bologna, IT)

**Introduction** The success of TKA depends on restoration of limb alignment, accurate implant position and optimal gap balancing. Possible sources of errors in navigated measured resection technique could be caused by the distorted landmarks in a deformed arthritic knee; the fact that soft tissues releases often affect flexion and extension gaps asymmetrically contribute to inaccuracies in restoring normal balance; variation of the saw cut within the cutting jig; and human errors in landmarks acquisition.
**Materials and methods** Eighty-five navigated primary TKA were performed between 2007 and 2009. All prostheses were implanted with the aid of surgical navigation (Stryker Knee Navigation). Once the distal femur and proximal tibia cuts were made the extension gap was measured by the navigation system. Gaps in extension and at 90° of flexion and femoral component rotations recorded in the navigation system. The navigation system was used to assess dynamically the knee deformity in both frontal and sagittal planes of the osteoarthritic knee.
**Results** The mean difference between flexion and extension (F/E) gap was 0.7 mm, and more balanced and equal extension and flexion gaps were obtained in patients with neutral alignment in the sagittal plane, whereas knee deformities associated to hyperextension and excessive laxity demonstrated the higher difference in F/E gap, and these differences proved to be statistically significant. The mean medial and lateral asymmetry was 1.7 mm (wider in the lateral side for varus and in the medial side in valgus).
**Discussion** Measured resection technique assisted by navigation system allowed to obtain an almost equal flexion and extension gap, resulting in an overall good tensioning of the collateral ligaments. The data obtained in this series showed as the navigation system was fundamental in guiding the surgeon in balancing the flexion with the extension gaps and in adjusting the thickness of the femoral resections on the base of the osteoarthritic knee deformity. Interestingly we noted that in patients with flexion contracture deformity we performed further resection of the distal femur and proximal tibia. In subjects with hyperextension deformity we have, instead, performed thinner resections, and these differences proved to be statistically significant.
**Conclusions** The surgical navigation was able to achieve precise soft tissue balance, and can be an useful and effective aid in correcting both frontal and sagittal plane deformities in TKA. Moreover it was fundamental in guiding the surgeon in balancing the flexion with the extension gaps and in adjusting the thickness of the femoral resections on the base of the osteoarthritic knee deformity.

### Patient reported outcome measures in the new Persona total knee arthroplasty

M. Ghiara*, A. Combi, L. Perticarini, S.M.P. Rossi, F. Benazzo

Policlinico San Matteo (Pavia, IT)

**Introduction** The new Persona total knee arthroplasty gave in the short follow-up excellent clinical and functional results. The patients answered to the questions of the Patient Reported Outcome Measures, used in the UK to have an objective and impartial evaluation of the surgical results.
**Materials and methods** From 15 May 2012 to February 2013 we implanted 101 Persona prostheses, 62 patients had a mean 5 month follow-up (range 3–8 months). The mean age of this group (23 males, 38 females) was 69 years (range 53–86 years). We used the score of the English Patient Reported Outcome Measures, that is the Oxford Knee Score, the EQ-5D Index and the EQ-VAS. We compared these results with a group of 58 patients, treated in the same centre but with the implant of the prosthesis Nex-Gen LPS (mean follow-up almost 9 months) and with the group of the English PROMs (beyond 43,000 patients, mean follow-up 6 months).
**Results** The post-operative OKS had a mean value of 39.6 points (range 11–47) with a mean improvement of 18.2 points (range −5 to 35). The post-operative EQ-5D Index had a mean value of 0.848 (range 0.274–1) with a mean improvement of 0.632 (range 0–1.181). The postoperative EQ-VAS had a mean value of 76.9 points (range 20–100) with a mean improvement of 36.7 points (range −30 to 95). The patients defined the surgical results as excellent in 46 % of the cases, very good in 16.3 %, good in 26.3 %, fair in 8.2 % and poor in 3.2 %.
**Discussion** The evaluation of the patients of the Persona group showed an excellent improvement of the OKS, specific for the knee, with always greater values than the values of the control groups (except one case with a substantially identical value). The EQ-5D Index and EQ-VAS scores showed less homogeneous results, because these scores reflect not only the status of the knee but also the general health and the emotional condition.
**Conclusions** The results showed an excellent outcome of this implant, similar to the results obtained with a patients group with longer follow-up and a well-known implant (Nex Gen LPS Flex). Persona, despite the short follow-up, can be defined as an implant that fulfils the expectations and the requirements of the patients already in the early postoperative period.

### Notching of anterior femoral cortex and risk of peri-prosthetic fractures after total knee arthroplasty

P. Sessa, A. Della Rocca, F. Gabriele, G. Cinotti

Orthopaedic Department, University La Sapienza (Rome, IT)

**Introduction** Peri-prosthetic fractures after total knee and hip arthroplasty have been reported with an increased incidence in the recent years. Although definite risk factors, beside a reduced BMD and high co-morbidity, are yet to be identified, it has been suggested that a violation of the anterior femoral cortex in the positioning of total knee arthroplasty (TKA) may increase the risk of sovracondylar fractures in operated knees. Aim of this study was to assess the incidence of femoral fractures in patients undergoing TKA with or without anterior femoral notching.
**Materials and methods** We retrospectively analysed postoperative radiographs of 160 patients who underwent TKA. Forty-six of these (29 %) showed an anterior notching of femoral cortex, the entity of which was assessed in 4 degrees according to Tayside classification. A minimum of 5 years after surgery (range 5–8 years), patients were asked if they had had any trauma, with or without fractures, during the follow-up period at the operated or contralateral limb.
**Results** Of the 46 patients included in the study, the anterior femoral notching was classified as grade I in 28 cases, grade II in 13 and grade III in 5. In no patient a grade 4 notching was observed. No periprosthetic fractures was reported by any patient, although 3 of them had an early orthopaedic visit due to a trauma in the operated limb.
**Discussion** Anterior notching of femoral cortex may occur inadvertently or deliberately, the latter to avoid the use of a large size with femoral condyles overhanging to restore the posterior condylar offset. In this respect it may be useful to assess the medium and long-term clinical history of patients with notching of the anterior femoral cortex.
**Conclusions** The results of present investigation have shown that grade I to III of anterior femoral notching does not increase the incidence of periprosthetic fracture at medium term follow-up after TKA.

### *C54–PROSTHESES-KNEE III*

### Assisted pre-operative navigation in knee prosthesis: preliminary results of a prospective randomised and controlled study

V. Sansone*[1], C. Bonora[1], N. Ursino[2], L. Tagliabue[2], S. Scarponi[1], V. Pascale[1]

[1]Università degli Studi di Milano (Milan, IT);
[2]Istituto Ortopedico Galeazzi (Milan, IT)

**Introduction** Navigation MRI-based surgery has been proposed as a technique to improve implant alignment and soft tissue balancing during total knee arthroplasty (TKA). A digital 3D reconstruction of the knee allows the production of custom-made plastic guides that should ensure proper bone resection and implant placement. The aim of this study is to establish whether navigation-assisted surgery leads to an improvement in the early radiological, functional and clinical parameters over conventional TKA techniques.
**Materials and methods** This is a prospective, randomised, controlled study. Between January 2010 and February 2011, fifty consecutive patients received a TKA for primary osteoarthritis. In all cases a Vanguard, posterior-stabilised prosthesis was implanted (Biomet UK) Twenty five patients were operated using the MRI-based Signature knee navigation system and 25 patients with conventional instrumentation. We evaluated the post-operative radiographic alignment, pain and functional parameters using WOMAC, FIM and Barthel Scores. The duration of the surgery and blood loss were recorded, and any changes to the pre-operative planning (e.g. bone cuts, component sizes) were also noted. All patients were followed-up at 15 and 45 days and at 1 year.
**Results** At the 1 year follow-up there were no statistical differences between the two groups for all clinical, functional and radiographical parameters. In the navigated group the need for blood transfusion was reduced by 50 %. The prosthesis alignment and rotation were correct in both groups, and the patella position was normal according to the Insall-Salvati Ratio. There was no radiographical evidence of loosening. In 20 % of the navigated patients the implanted femoral component was smaller than planned, in 28 % a tibial recutting was necessary and mean surgical time was 17 min longer.
**Discussion** Our preliminary results in 25 preoperative assisted TKAs show good restoration of limb alignment and component rotation in all cases. Component sizing seems to be less accurate, although the choice of a 2 mm-smaller femoral component may be highly subjective. The operative times were similar, although the first cases of the PAN group were surely affected by a learning curve and by the prudence of the surgeons who double-checked the positioning of the jigs using conventional means.
**Conclusions** Further larger controlled studies, with longer follow-up and a proper analysis of cost-effectiveness will be necessary to validate this new system, although our initial results seem to be encouraging.

### Use of intravenous tranexamic acid and femoral nerve block to reduce post-operative blood loss after total knee arthroplasty

M.C. Giordano*[1], S. Rosi[1], P. Rubcich[2], E. Roefaro[1], M. Villa[1], N. Ventricelli[1], A. Mazzarino[1]

[1]Casa di Cura Guarnieri (Rome, IT);
[2]Ospedale S. Andrea (Rome, IT)

**Introduction** The blood losses that occur with total knee arthroplasty (TKA) are due to fibrinolysis stimulation after surgical trauma and tourniquet use, and to increased cardiac output secondary to the related pain and stress. The purpose of this retrospective study is to evaluate the combined effectiveness of intravenous tranexamic acid (TA) to counteract fibrinolysis and a femoral nerve block in order to reduce the post-operative pain.

**Materials and methods** We carried out from March 2011 to December 2012 113 cemented TKA (Depuy PFC-sigma). Twenty-two patients were excluded (<11.0 haemoglobin, with cardiac risk factors, with a history of thrombosis or embolic disease). The remaining 91 patients (group 1) that meet the inclusion criteria received a dose of 500 mg of tranexamic acid before tourniquet release and 500 mg 3 h after the operation. All these patients received at the end of surgery a femoral nerve block with 10 cc of 10 % ropivacaine. An equal number of patients (91) operated consecutively TKA (same model of cemented prosthesis) without the use of tranexamic acid and femoral nerve block, before March 2011, represented the control group (group 2). Drainage was removed in all patients after 24 h. Blood pressure, heart rate, haemoglobin (Hb) levels, haematocrit (Ht) levels, drained blood volume, allogenic blood transfusion rates and analgesic consumption were recorded.

**Results** The values of Hb, Ht, blood loss and units of allogenic blood transfusion were significantly lower in the first group. The average consumption of analgesics and the recovery of sitting and walking was essentially the same in both groups. No patient in the study reported episodes of deep vein thrombosis or pulmonary embolism.

**Discussion** The effectiveness of the use of tranexamic acid in reducing blood loss is demonstrated in the literature. The association of a femoral nerve block allows to further reduce these losses by acting on the effect that pain has on cardiac output.

**Conclusions** The association tranexamic acid-femoral nerve block significantly reduces blood loss and the need of blood reinfusion after TKA surgery.

### Short term follow-up in the new Persona total knee arthroplasty

M. Ghiara*, A. Combi, L. Perticarini, S.M.P. Rossi, F. Benazzo

Policlinico San Matteo (Pavia, IT)

**Introduction** In the last years the number of total knee arthroplasty is constantly increasing, but despite this quantitative rise and the innovations of the prosthetic design, the rate of satisfaction of the patients reported in literature is between 75 and 89 %. A new prosthetic design has been created, that reproduces the bone anatomy, reduces the risk of impingement with soft tissues and optimizes the tibial coverage by an asymmetric shape of the tibial baseplate.

**Materials and methods** From 15 May 2012 to February 2013 we implanted 101 Persona total knee arthroplasties, which 62 implants (one bilateral, 23 males and 38 females) with a follow-up between 3 and 8 months (mean 5 months). The mean age of the patients was 69 years, the right side was treated in 30 cases and the left one in 32 cases, the knee was varus in 50 cases and valgus in 12 cases, a preoperative flexion contracture was present in 15 cases. We have followed the patients at 1, 3 and 6 months from surgery and with X-rays at 3 and 6 months.

**Results** The patients had an excellent outcome, achieving a full range of motion and a fast functional recovery in the early postoperative months; only two patients had a new surgery for a complication related to the knee, that is a new capsular suture for the its breakage and an arthroscopic lavage for recurrent blood swelling. Other two patients presented a DVT, that had a slackening-off of the rehabilitation. At the X-rays nobody had signs of loosening or significant radiolucent lines at 3 and 6 months.

**Discussion** Despite the short follow-up the patients obtained an excellent functional recovery and a clinical improvement rather the pre-operative period. We confirmed this satisfying clinical evaluation by the Patient Reported Outcome Measures questionnaires, allowing the patients to unveil these good clinical and functional results after a short follow-up.

**Conclusions** The total knee arthroplasty Persona showed to be an innovating implant that allows the patient already after few months to obtain excellent clinical and functional results.

### Tracking of patello-femoral joint kinematic in navigated total knee replacement

A. Feliciangeli*, A. Ensini, C. Belvedere, A. Timoncini, P. Barbadoro, A. Leardini, S. Giannini

Istituto Ortopedico Rizzoli (Bologna, IT)

**Introduction** The surgical navigation in total knee replacement (TKR) provides data for osteotomies and for the implant based on the collection of known bony landmarks and measurements of the tibio-femoral articulation. The TKR system also alters the kinematics of the patello-femoral joint (PFJ) that, until now, has been excluded from kinematic measurements in navigation systems. The purpose of the study is to describe the evaluation of patello-femoral kinematics intra-operatively in two groups of patients operated with two different models of TKR with patellar resurfacing.

**Materials and methods** Twenty patients with knee osteoarthritis were divided in two groups of 10 patients each and operated with two different models of prosthesis postero-stabilized with patellar component. A standard navigation system was used for all implants. A second specific system with a dedicated tracker on the anterior patellar facet was added for PFJ tracking. After the execution of the TKR with standard navigated technique, the patella has been replaced and the resection plans were subjected to instrumental verification. An original protocol was developed for the acquisition of kinematics and landmarks also for the patella.

**Results** The new procedure was successfully performed in all cases without complications, with elongated surgical time of 30 min. The final alignment of the limbs was between 0.5°, the patella replaced joint was $0.4 \pm 1.2$ mm thinner than the pre-operative, the cut of the patella was $0.4° \pm 4.1°$ sideways tilt. The kinematics of the patella-femoral joint post-implantation in the patello-femoral joint showed on average a decline, incline and a medial–lateral translation respectively of $66.9° \pm 8.5°$ (with a reduction of at least 15.6° and a maximum deflection of 82.5°), $8.0° \pm 3.1°$ (−5.3° to 2.8°), $5.3 \pm 2.0$ mm (−5.5 to 0.2 mm) regardless of the prosthetic design.

**Discussion** This first experience supports the feasibility, relevance and effectiveness of the patello-femoral tracking in navigated TKR. The results show that these measurements of the PFJ are valid for the patellar resurfacing. The surgeon may use this system to perform a more comprehensive evaluation of the anatomy and kinematics of the knee pre-and post-implantation.

**Conclusions** In the future, if this procedure will be performed regularly in navigated TKR, abnormal sliding of the patella may be corrected intra-operatively and patellar osteotomy preparation may be performed more cautious as to achieve correct positioning of the prosthetic components of the femur, tibia and also of the patella.

### Anterior knee pain after TKA by para-patellar medial approach: ecographic assessment of quadriceps tendon

M.F. Surace, R. Alberio*, L. Monestier, G.V.M. Regazzola, P. Cherubino

Dipartimento di Biotecnologie e Scienze della Vita, Clinica Ortopedica, Università dell'Insubria (Varese, IT)

**Introduction** Anterior pain is one of the most frequent complication referred by patients who underwent TKA. The onset of patellar pain is related to different factors, as reported in literature. In this study we assessed which factors can influence clinical outcome in TKA implant.
**Materials and methods** We examined 51 patients who underwent TKA implant for primary gonarthritis. Two different sub-populations were compared: 24 patients with anterior knee pain (average age 70.5 years, BMI 30 kg/m$^2$, right knee treated in 11 cases; follow-up 37 months) and 27 asymptomatic patients (average age 73 years, BMI 26.5 kg/m$^2$, right knee treated in 13 cases; follow-up 47 months). Implant (Scorpio NRG, Stryker), surgeon, surgical approach were identical in both populations. Patients were subjected to clinical (ROM, femoral quadriceps dimension, clinical and functional KSS), radiographic (anatomic and mechanical axis of lower extremity, Insall score, femoral notching, patellar inclination and congruency with the surface of femoral trochlea) and US assessments.
**Results** Clinical evaluation of the whole population reported a significant increase of ROM and clinical-functional KSS, as well as patellar rasping regression. No considerable differences were highlighted between sub-populations. Post-operative KSS score was significantly related to BMI of the patient and anterior knee pain onset. Follow-up radiographs showed no differences compared to pre-operative ones; conversely, ultrasound assessment of quadriceps tendon reported enthesopathy features (distal insertional fibrosis and calcifications within tendon) in the whole population. No significant differences were documented between two sub-populations by radiographic and US evaluation.
**Discussion** The results could not be influenced by age, side of treated knee, surgical technique or surgeon because they all were identical for all patients. Post-operative onset of anterior knee is related to patient BMI and the consequent improvement of KSS score: in this way, obesity and pain perception of the singular patient might be the most influencing factors. At the contrary, factors reducing anterior knee onset couldn't be defined as we don't report significant differences between two sub-populations both clinical and radiographic-US assessment.
**Conclusions** Anterior knee pain in patients who underwent TKA is related to quadriceps tendon structural alterations, eventually caused by trans-tendon approach. Widespread enthesopathy and the presence of calcifications within tendon or sheath is not related to pain onset.

---

### *C55–PROSTHESES-KNEE IV*

### Scorpio implant as primary total knee arthroplasty: short-midterm results and anterior knee pain onset

M.F. Surace, L. Monestier*, D. Pin, D. Tanas, A. Testa, P. Cherubino

Dipartimento di Biotecnologie e Scienze della Vita, Clinica Ortopedica, Università dell'Insubria (Varese, IT)

**Introduction** Total knee arthroplasty (TKA) is the gold standard in treatment of primary gonarthritis; posterior stabilization is still controversial. Clinical and radiographic parameters were assessed in two different populations with CR or PS implants.
**Materials and methods** Eighty-nine cemented TKAs were implanted by medial para-patellar approach from 2007 to 2012; 33 Scorpio NRG CR and 56 Scorpio NRG PS (Stryker). Pre-operative clinical evaluation was based on ROM and Knee Society Score (KSS); at 3 years follow-up, each patient was clinically assessed by means of ROM, knee stability, anterior knee pain, KSS and Knee Injury and Osteoarthritics Outcome Score (KOOS). Pre-operative and post-operative radiographs were obtained and examined.
**Results** Patients showed a significant increase of clinical KSS (from pre-operative 45 points to post-operative 80 points) and functional KSS (from pre-operative 55 points to post-operative 86 points); ROM considerably increased after surgery (flexion from 113° to 116°, extension from −3° to 0°); no significant differences were nevertheless demonstrated between CR and PS implants. There was no difference in patients about KOOS, knee stability and anterior knee pain after surgery. Correlations were found between age and functional KSS, as the latter and anterior-medial knee pain. Active patellar rasping was positive in 11 % patients, while passive rasping in 25 %; palpation of patella was painful in 18 % cases.
**Discussion** Absence of significant differences between CR and PS populations is related to many variables (age, BMI, Q angle, pre-op ROM, personal tolerability to pain) that influence clinical post-operative outcome in TKA implant. Increase of tibial slope is recommended in CR implants, as it reduces PCL tension and improves knee flexion.
**Conclusions** TKA leads to significant improvement of life quality in patients with gonarthritis; however, CR or PS implant choice is not the relevant aspect in influencing clinical post-op outcome.

### Patient specific instruments: marketing or a real innovation in total knee replacement? Prospective study of 15 pre-navigated total knee replacements with a mean 18-month follow-up

G. Costacurta, N. Calzavara, E. Scalco, C. Chemello*

Ospedale Asiago (Asiago, IT)

**Introduction** Custom made surgery seems to be the future of total knee replacement (TKR). Patients specific instrument (PSI) helps the surgeon to perform a real tissue sparing surgery (TSS) and it allows to customize technique and to standardize results. The aim of our study was to illustrate advantages and disadvantages of PSI.
**Materials and methods** Fifteen patients, affected by knee advance osteoarthritis (10 varus, 5 valgus) and by axial deviation exceeding 3°, underwent TKR (Advance Prophecy Wright). TC was performed following strict protocols during pre-operative examination. Each planning has been revised by us and then confirmed before starting productions. Protocol included: at the baseline a preoperative lower limb teleradiography and a staging according to the Knee Society Score (KSS), 3 months after surgery a new teleradiography and a restaging with KSS after 2 years. Post-operative axes were compared to the planed ones and the following angles were examined: between anatomical and mechanical femoral axis (αAAM), femoral slope (FSF), tibial slope (ST) and tibial alignment (αCT). Negative outcome was considered as axial deviations ±2 for FSF, ST and ±3 for αCT and αAAM.
**Results** All patients achieved an excellent clinical and functional results according to KSS. In 2 cases a higher tibial insert of 12–14 mm was implanted and in only one case was necessary a 17 mm insert. Final knee flexion was necessary always more than 105° and in 84 % cases higher than 110°. Only 1 error of the final αAAM. Three errors of the ST (inversion in one case), 4 of the femoral component flexion (1 was hyperextended), 2 of αCT were observed. Mal position of both components was detected in 2 cases. Two lateral release and no patellar resurfacing were performed.
**Discussion** At the beginning we had problems with the femoral flexion of the shield which were subsequently solved by the introduction of a third point of contact. The final overall alignment of the limb was wrong only in one case. We found an increased risk of error and difficulty in the unbalanced knees, in which we initially used bigger inserts for excessive cuts.
**Conclusions** This technique allows to perform a TSS surgery, to reduce surgical time, to obtain optimal and reproducible alignments. Surely it requires an adequate learning curve as it can lead to significant and unpleasant positioning errors. We recommend to perform tibial cut $<2$ mm compared to the scheduled in instable knee to obtain balanced knees without using too thick inserts.

### How misalignment influences UKA survivorship: a comparative study between all-poly and metal back UKAs

F. Zambianchi*, V. Digennaro, A. Giorgini, G. Grandi, F. Catani

Azienda Ospedaliero-Universitaria Policlinico di Modena (Modena, IT)

**Introduction** The mid-term outcome of unicompartmental knee arthroplasty (UKA) depends on patient selection, age, gender and level of activity. Despite satisfying clinical and radiographic outcomes, UKA revision rate is still high if compared to total knee replacement (TKR). In this study we aimed to determine whether: 1. the anatomic femoro-tibial angle (aFTA) and the tibial component alignment on the sagittal plane, influence implant survivorship; 2. prosthetic design influences clinical results, through a comparison between metal back and all-poly UKAs.
**Materials and methods** Between 2003 and 2007, three surgeons performed 195 medial UKAs on 176 patients with a mean age of 67.8 years. One-hundred and forty-seven cases were included in the study: 72 all-poly, 75 metal back. On the pre- and post-operative X-rays the following angles were measured: mechanical femoro-tibial angle, aFTA, tibial posterior slope, Cartier angle (pre-operative), coronal tibial and femoral component varus/valgus alignment (post-operative). At last follow-up patients were clinically evaluated through KOOS questionnaire.
**Results** At a mean follow-up of 61 months, the mean UKA survivorship resulted 93.5 %. Eleven implants underwent revision: 10 all-poly, 1 metal-back. Although no differences were reported on the radiographic outcome between the two prosthetic designs, revisioned and not-revisioned implants reported statistically significant radiographic differences. In particular, aFTA ($p = 0.027$): $1.09° \pm 4°$ valgus for revisioned and $3.17° \pm 3.23°$ valgus for not-revisioned UKAs; varus/valgus alignment of the tibial component ($p = 0.0004$): $0.18° \pm 2.48°$ varus for revisioned and $3.75° \pm 3.73°$ varus for not-revisioned UKAs. No clinical outcome differences were reported in 4 out of 5 KOOS domains.
**Discussion** Our data suggest that tibial component valgus alignment and hypo-correction of pre-operative coronal deformity are suggestive of UKA failure. As evidenced by KOOS results, UKA reports satisfactory mid-term results. In general, there are no overall differences between all-poly and metal back implants.
**Conclusions** Experience and surgical technique are essential in UKA surgery: the preservation of the epiphyseal axis, avoiding excessive or insufficient corrections of the pre-operative limb alignment predict better UKA outcomes, while prosthetic design, models and fixation geometry do not influence clinical results and mid-term survivorship.

### Bi-unicompartmental knee implants: long-term results

M. Corbella*, M. Massaro, F. Verde, S. Romagnoli

Istituto Ortopedico Galeazzi (Milan, IT)

**Introduction** The bi-unicompartmental knee prosthesis is a system that uses two independent Uni implants, femoro-tibial medial and lateral, to preserve the tibial eminence with the anterior cruciate ligament (ACL). Although in most of the prosthetic knee systems the cruciate ligaments are sacrificed or only the LCP is preserved, a goal of the research was to reproduce the normal kinematics of the knee joint, replacing only the worn areas and respecting capsule and ligaments.
**Materials and methods** From January 2001 to January 2012, we performed 71 Bi-unicompartmental knee implant in femoral-tibial medial and lateral OA (osteoarthritits), which were followed prospectively. Mean follow-up was 6 years (max. 11 years). Patients were followed-up every year for the first 3 years and every two years later on. At the most recent follow-up, 71 knees in 68 patients were evaluated.
**Results** Clinically, the Hospital for Special Surgery Knee Score increased from an average of 59 points preoperatively (42 to 68) to 92 (70 to 100). The mean pre-operative ROM was 104.7° (80° to 130°) while at final follow-up the mean ROM was 124.7 (from 102° to 136°). 64 patients had no pain (92%), 2 patients had a slight or occasional pain (4%). At the time of the last follow-up 64 patients (92%) were enthusiastic regarding the procedure, 2 patients (4%) were satisfied with the procedure, 2 patients experienced a failure of the implant. The cumulative survival rate at ten years was 96.01% (CI 97% to 100%). At 14 years, the cumulative survival rate was 94.90% (CI 81% to 100%).
**Conclusions** The Bi-UNI procedure, although not commonly performed, seems to provide a high level of functionality and knee kinematics retaining, essential characteristics of the normal knee. However, it requires a relevant learning curve.

### Unicompartmental medial knee replacement with patient-specific instrumentation: preliminary evaluation

E. Prospero*, C. Bait, M. Cervellin, M. Denti, A. Quaglia, A. Redaelli, P. Volpi

Humanitas Clinical and Research Centre (Rozzano, IT)

**Introduction** In recent years we observed an increased number of patients affected by knee osteoarthritis, with a consequent raising in knee replacement procedures. The constant development and research on biocompatible materials, biotechnology and the improvement of surgical techniques, has led to the development and design of unicompartmental prosthesis ever more efficient, able to better restore biomechanics and kinematics allowing a more rapid return to daily life.
**Materials and methods** A pilot study was conducted with 16 patients (16 knees) with isolated medial unicompartmental osteoarthritis of the knee in order to evaluate the alignment of the knee and the tibial slope before and after implantation of unicompartmental prosthesis using a patient specific instrumentation (PSI). We performed the pre-operative planning using a dedicated software. We performed standing long leg X-rays before surgery and after 15 days from surgical procedure. We measured mechanical axis of lower operated limb and tibial slope in degrees comparing these data with the ones obtained in pre-operative planning.

**Results** Regarding the mechanical axis, the acquired data showed a discrepancy maximum of 0.5° between pre-operative planning and post-operative data. We obtained a mean value of mechanical axis of 1.73° ± 1.21° in varus compared with a mean of 1.58° ± 1.21° of pre-operative varus alignment. Concerning the tibial slope we observed post-operative value of 5.12° ± 0.64° compared to a pre-operative tibial slope value of 5.37° ± 0.64°. Only in one case there was a discrepancy between the size of the final implant and the size estimated during the pre-operative planning.
**Discussion** The data obtained showed that we were able to obtain a correction of the mechanical axis (hip–knee–foot) similar to that estimated during the preoperative planning. Concerning the tibial slope, we observed a small difference between pre-operative planning and post-operative control.
**Conclusions** Using patients specific instrumentation, we were able to achieve very satisfactory results with regard to the correction of the mechanical axis and the tibial slope. We found a nearly complete concordance between the estimated prosthetic components and the implanted ones.

### Long-term survival results in lateral unicompartmental knee replacement

F. Verde*, M. Massaro, M. Corbella, S. Romagnoli

Istituto Ortopedico Galeazzi (Milan, IT)

**Introduction** Unicompartmental knee replacement (UKR) has well known advantages over total knee replacement such as less invasivity, respect of both cruciate ligaments, better function and less morbidity. However, survival rates of UKR were typically inferior to the survival rates of total knee replacement. The main opinion was that higher failure rate compared to the total knee replacement was due to the effects of other compartments degeneration and polyethylene wear. Despite the past, recent surveys show an evident improvement in survivorship rate, probably based on the large diffusion of the UNI implants surgical technique and prosthesis, and in a consequent improvement in indication. The long term clinical results are the hardest base in order to confirm or not the general opinion.
**Materials and methods** From February 1991 to January 2010 we performed 184 UKR side in 176 patients. The follow-up was completed in February 2012. Clinically, the indications for surgery were: knee pain, absence of patello-femoral joint symptoms, flexion contracture of <10°, range of motion> 80°, and valgus deformity <15°. The functionality of the post-operative knee was evaluated with the Hospital for Special Surgery Knee Score before and after the surgery.
**Results** At the time of the last follow-up, 129 patients (82.4%) were enthusiastic regarding the procedure, 27 patients (17.6%) were satisfied with the procedure, and only one patient (0.6 %) was not satisfied. There were 11 revisions: one occurred five years after surgery for the capsule-ligament instability and the patellofemoral joint degeneration. In two more cases, 5,5 and 3 years after implantation respectively, failure of LCA and capsulo-ligamentous instability were reported.
**Conclusions** The indications for UKR are defined. The procedure is recommended for osteoarthritis in both medial and lateral compartments. The axial deformity, varus or valgus, must be corrected during surgery. Although not well reported in the literature, the treatment of lateral fractures of the tibial plateau is, in our view, a problem especially in young patients. We believe that the use of a UNI in these cases, when possible, and reduces the risk of complications such as post-operative infections and the rate of stiffness seen with TKA. Based on the correct indications, surgical experience and an appropriate instrument trays, the lateral unicompartmental prosthesis is a safe and reliable choice for the treatment of primary and secondary unicompartmental osteoarthritis of the knee.

### *C56–PROSTHESES-HIP I*

### The epidemiology of hip and knee osteoarthritis: focus on the gender differences analysing the data of the Puglia Region Register of Orthopaedic Prosthetic Implants

S. Mudoni*[1], G. Gravina[1], P. Maggiolini[1], M.T. Balducci[1], C. Germinario[1], A. Aloisi[2], U. Biasi[3], A. Bozzi[4], L. Brina[5], V. Caiaffa[6], M. Capozzi[7], G. Carchia[8], R. Colasuonno[9], G. De Giorgi[10], F.P. Di Carlo[11], V. Fantetti[12], F. Fitto[13], V.N. Galante[14], P. Galluccio[15], C. La Macchia[16], R. Laforgia[17], D. Laghezza[18], F. Lagrasta[19], A. Latela[20], A. Leo[21], L. Limonciello[11], G. Lo Bianco[22], F. Loconte[23], S. Lotito[24], A. Macchiarola[25], R. Mangialardi[26], W. Mega[27], A. Mocci[28], C. Monteleone[29], B. Moretti[10], T. Nevoli[30], A. Pagano[31], D. Panetta[32], L. Petrelli[33], R. Recchia[34], L. Refolo[35], N. Ricchiuti[36], A. Ricciardiello[37], G. Rollo[38], V. Ruggiero[39], F. Russo[40], V. Scarano[41], M. Scialpi[42], F. Specchiulli[25], G. Spera[43], V. Tempesta[44], E. Vigilante[45], A. Villani[46]

[1]Osservatorio Epidemiologico Regione Puglia (Bari, IT);
[2]P.O. "S. Caterina Novella" (Galatina, IT);
[3]P.O. "Vito Fazzi" (Lecce, IT);
[4]Casa di Cura Bernardini (Taranto, IT);
[5]P.O. "San Paolo" (Bari, IT);
[6]P.O. SS. Annunziata (Taranto, IT);
[7]Casa di Cura Santa Maria (Bari, IT);
[8]P.O. "S. Camillo De Lellis" (Manfredonia, IT);
[9]P.O. "Monsignor R. Dimiccoli" (Barletta, IT);
[10]A.O.U. Policlinico (Bari, IT);
[11]Cliniche Riunite Villa Serena e San Francesco (Foggia, IT);
[12]P.O. "T. Masselli Mascia" (San Severo, IT);
[13]Casa di Cura Città di Lecce (Lecce, IT);
[14]P.O. Occidentale (Castellaneta, IT);
[15]Casa di Cura Villa Bianca (Lecce, IT);
[16]P.O. "Don Tonino Bello" (Molfetta, IT);
[17]Casa di Cura Santa Rita (Bari, IT);
[18]P.O. Bisceglie-Trani (Bisceglie-Trani, IT);
[19]P.O. "Caduti In Guerra" (Canosa, IT);
[20]P.O. "San Giacomo" (Monopoli, IT);
[21]P.O. "S. Giuseppe da Copertino" (Copertino-Nardò, IT);
[22]Casa Di Cura Villa Lucia (Conversano, IT);
[23]P.O. Ostuni (Ostuni, IT);
[24]P.O. "L. Bonomo" (Andria, IT);
[25]A.O.U. Ospedali Riuniti (Foggia, IT);
[26]Casa di Cura San Camillo (Taranto, IT);
[27]P.O. "Sacro Cuore di Gesù" (Gallipoli, IT);
[28]Casa di Cura Anthea (Bari, IT);
[29]P.O. Valle d'Itria (Martina Franca, IT);
[30]P.O. "San Marco" (Grottaglie, IT);
[31]P.O. "A. Perrino" (Brindisi, IT);
[32]Casa di Cura F. D'Amore (Taranto, IT);
[33]P.O "Umberto I" (Altamura, IT);
[34]P.O. "S. Maria degli Angeli" (Putignano, IT);
[35]P.O. "Veris delli Ponti" (Scorrano, IT);
[36]P.O. "Camberlingo" (Francavilla-Ceglie, IT);
[37]Casa Bianca Hospital (Cassano, IT);
[38]E.E. Miulli (Acquaviva delle Fonti, IT);
[39]P.O. "Umberto I" (Corato, IT);
[40]P.O. "F. Ferrari" (Casarano, IT);
[41]P.O. "G. Tatarella" (Cerignola, IT);
[42]P.O. Orientale (Manduria, IT);
[43]P.O. Di Venere (Bari, IT);
[44]E.E. "Cardinale G. Panico" (Tricase, IT);

[45]IRCCS Casa Sollievo della Sofferenza (S.G. Rotondo, IT);
[46]P.O. "Lastaria" (Lucera, IT)

**Introduction** Coxarthrosis and gonarthrosis are the most important forms of osteoarthritis for frequency and for induction of disability, affecting more female than male, especially after the age of 55. The aim of our study is to provide a focus on gender differences in evaluation of primary arthrosis, analysing the data of the Puglia Region Register of Orthopaedic Prosthetic Implants (RIPO).
**Materials and methods** Descriptive analysis has been led on the data presents in the paper RIPO cards, transmitted from the Puglia Orthopaedics Unit to the "Osservatorio Epidemiologico Regione Puglia" (OER), the regional centre of coordination. OER staff entered collected data and analysed them using the statistical software Epi Info 3.5. The data refers to the year 2011: 4,238 hip prosthesis and 2,823 knee replacements; the record linkage with the hospital discharge forms (SDO) showed a completeness of accession to the register of 96 % for the hip and 90 % for the knee.
**Results** Ninety-four percent (94 %) (n = 3,977) of hip prosthesis surgery are primary; of these, fractures of the neck and/or femoral head and the primary arthrosis were the most common causes. Eighty-six point nine percent (86.9 %) of coxarthrosis surgery occurred after 55 years, 12.2 % between 40 and 55 years and 0.9 % before the age of 40. In females, prevalence increases with age: 38 % before the age of 40, 53 % between 40 and 55 years and 62 % after 55. For knee prostheses (2,681 primary surgery), the most (89.4 %) frequent cause was the gonarthrosis. Female prevalence was equal to 75 % for all age groups, except before the age of 40 where there has been equality between the sexes (50 %).
**Discussion** Females and advanced age are the two constitutional factors that increase the prevalence of degenerative disease process. Our study underlines the relationship, putting themselves in line with the scientific literature.
**Conclusions** Our findings highlight the importance of preventing this disease, which represents a major cause of disability in millions of people. Adopting correct lifestyles could control risk factors such as for obesity and the overhead joints. The prosthetic implantology register allows the monitoring of implanted medical devices and their traceability.

### Total hip arthroplasty after acetabular fracture: our experience

A. Aprato*[1], M. Gastaldo[2], M. Favuto[1], D. Colombero[1], A. Massè[1]

[1]Orthopaedic Department, School of Medicine, San Luigi Hospital of Orbassano, University of Turin (Turin, IT);
[2]Orthopaedic Department, School of Medicine, University of Turin (Turin, IT)

**Introduction** Aim of this study is to evaluate the clinical results of total hip arthroplasty after conservative treatment or internal fixation for an acetabular fracture.
**Materials and methods** Twenty-seven patients were enrolled: gender, age, time from trauma to arthroplasty and follow–up were recorded. Analysing the pre-operative X-rays, the acetabular fractures were classified as elementary or complex with a custom made modified Letournel classification. Pre-implant X-rays were evaluated according to AAOS and Paprosky classifications. Used stem and cup were classified as primary, revision and reconstruction implants. All patients were clinically evaluated with modified Harris Hip Score (mHHS) and Western Ontario and McMaster Universities Arthritis Index (WOMAC) before arthroplasty and at the last follow-up.
**Results** Average age was 50 years, average time from trauma to arthroplasty was 59 months. Fractures were classified as elementary in 13 cases and as complex in 14 cases. Internal fixation has been performed in 16 patients, 11 cases underwent to surgical treatment at the time of fracture, 2 patients underwent a primary delayed treatment. As regards to implant, primary, revision and reconstructive implants were used respectively in 15, seven and five cases. Average surgical time has been 104 min. Average mHHS was 26 point pre-operatively and 83 points at an average follow-up of 30 months. Average WOMAC was 73 points pre-operatively and 15 points at the last follow-up. Patients who received a reconstruction arthroplasty showed worst results than patients who received a primary arthroplasty. Furthermore average time was significantly correlated with the type of implant (primary vs. reconstruction $p = 0.003$). Patients who underwent a conservative treatment showed a correlation with higher value of superior migration ($p = 0.014$) and teardrop osteolysis ($p = 0.045$) when compared to patients who underwent an internal fixation. Initial treatment time showed a correlation with type of used implant.
**Discussion** This study has several limits but presents the personal experience of the authors.
**Conclusions** This study suggests that arthroplasty after acetabular fracture has a good efficacy. Type of fracture may not influence clinical outcome and Paprosky's classification on pre-implant X-rays. Conservative treatment was correlated with a worse radiographic presentation before arthroplasty, furthermore outcome seems to be influenced by implant type.

### Reconstruction of hip biometry with the Fitmore stem: 5-year experience

M. Bombelli*

Ospedale Centrale (Bolzano, IT)

**Introduction** Modern THR must satisfy the criteria of long term durability and long term functional performance and both are related to bone preservation and correct biometric articular reconstruction.
**Materials and methods** Two hundreds Fitmore stems have been implanted between April 2008 and February 2013. All patients have been followed-up clinically and radiographically and evaluated by standard parameters and in 150 patients post-operative offset and leg length was compared by digital measurements to the contralateral not affected side.
**Results** In 129 patients leg length was restored and was equal within a margin of error of ±2 mm. In 45 patients the limb was lengthened on average of 0.44 mm, in 26 patients it was shortened on average of 0.26 mm. On 150 patients offset was identical within ±2 mm in 110 patients, increased by mean 4 mm in 29 patients and reduced by mean 3 mm in 11 patients.
**Discussion** Clinical and radiological results reflect the theory that short stems with adequate proximal metaphyseal stability preserve bone morphology within the first years of implantation and no evidence exists that this condition may alter with time.
**Conclusions** The Fitmore stem with various design configurations represents a valid tool for an accurate biometric reconstruction of the hip joint after THR.

### Total hip arthroplasty with stemless implants and neck preservation: our experience with Proxima Depuy Fitmore Zimmer and CFP Link

F. Cartesio*, G. Italiano, U. Corapi

Asp Messina P.O. "Fogliani" Milazzo (Milazzo, IT)

**Introduction** Hip prosthesis surgery with tissue sparing methods allows us to save the femoral neck and bone size heritage, important condition for both young and old people, given by the lengthening of life expectancy.

**Materials and methods** In our UOC from 2005 to 2012 were implanted 220 prosthesis stemless and neck preservation; the 95 % of patients were submitted to clinical and Rx-controls 35 days then chart and 3, 6 and 12 months and then every year after the first.

**Results** About 90 % of patients were checked. No septic or aseptic mobilisation to cases of surgical wound dehiscence with culture swabs negatives, a case of moderate heterotopic calcifications: one reduced post-traumatic dislocation with narcosis, a death after a year for aggravation o co-morbidity. The Harris hip score pre-operative average was 55, 90 results later controls.

**Discussion** We analysed the results obtained from the use of implants stemless and neck preservation Proxima Depuy Fitmore Zimmer and CFP Link, even in subjects no more young, still active and with bone quality still suitable for traditional implants. The clinical and X-rays results obtained and the high degree of patients satisfaction lay for this indication.

**Conclusions** The difference in design and the tribology of materials didn't determine significant differences in favour of a system than the other. The results will encourage the use even in old age patients provided with bone quality suitable for a stem. Surgical techniques, though not particularly complex, need a good learning curve and are therefore not entrust to surgeon neophytes.

### The importance of the Register of Orthopaedic Prosthetic Implantology (RIPO) in times of economic crisis

M.T. Balducci*[1], S. Mudoni[2], G. Gravina[2], P. Maggiolini[1], V. Siciliani[1], D. Parisi[1], M. Torre[3], C. Germinario[4], Gruppo di Lavoro Ortopedici Pugliesi[5]

[1]Osservatorio Epidemiologico Regione Puglia (Bari, IT);
[2]Scuola di Specializzazione Igiene e Medicina Preventiva, Università degli Studi di Bari (Bari, IT);
[3]Ufficio Statistica, CNESPS, Istituto Superiore di Sanità (Rome, IT);
[4]Dipartimento di Scienze Biomediche ed Oncologia Umana, Università degli Studi di Bari Aldo Moro (Bari, IT);
[5]Dirigenti Medici Unità Operative di Ortopedia e Traumatologia (Regione Puglia, IT)

**Introduction** A register of prosthetic implants, in addition to provides reliable observational statistics and collecting systematically essential information on the surgery and single endpoint, allows the a monitoring over time of the devices after their introduction in the market. The aim of the study was to perform a descriptive analysis of prosthetic implants of hip through the analysis of implanted devices registered in the Register of Orthopedic Prosthetic Implantology (RIPO).

**Materials and methods** On 15 January 2010, with Article 46 of the omnibus law, the Puglia Region established the register of orthopaedic implants from 2010, managed the Regional Epidemiological Observatory (OER). The transmission of data from the Divisions of Orthopaedics and Traumatology to OER is done by paper forms. The input data is performed by specialized personnel at OER, which takes care of the aspects of accuracy and security of the data. The reliability of the transmitted data to RIPO is ensured by the use of self-adhesive labels describing the prosthesis. From 1 January 2010 to 31 December 2011, OER received 8,368 forms. The data processing was performed using STATA software.

**Results** Among the causes of primary arthroplasty there were fractures of the femoral neck and primary osteoarthritis, mainly in the age group between 70 and 80 years, while the aseptic loosening of the cup and the dislocation were main cause for replanting. 58.5 % of cups/domes was of metallic material and was not cemented. Five percent (5 %) of the femoral stems was modular, 8 % was made of uncemented femoral stems for primary non-modular systems and anatomical, while most of the rods were in storage or non-modular. In 31.1 % of cases the head had a diameter of 28 mm. The coupling joint most commonly used in 2011 was the ceramic-polyethylene (40.8 %). Various models have been used to answer all surgical needs. Specifically, the number of manufacturers used was equal to 42.

**Discussion** The arthroplasty represent one of the greatest achievements of modern medicine and the current offer has allowed us to extend the range to diseases and age groups considered at risk.

**Conclusions** The orthopaedic registers represent a source of reliable scientific information and contribute to the establishment of a virtuous circle of learning/improvement useful to report more reliable facilities.

### *C57–PROSTHESES-HIP REVISIONS*

No Abstracts were received for this Section

### *C58–PROSTHESES-HIP II*

### Soft-tissue MRI changes in hip abductor muscles and peri-prosthetic tendons after total hip replacement: comparison between the postero-lateral and the transgluteal approaches

G. Ferretti*[1], A. Impagliazzo[2], S. Pelle[3]

[1]Azienda Ospedaliera San Giovanni, Addolorata (Rome, IT);
[2]Casa di Cura Paideia (Rome, IT);
[3]Dipartimento di Scienze Anatomiche Istologiche Medico-Legali e dell'Apparato Locomotore, Università degli Studi di Roma "La Sapienza" (Rome, IT)

**Introduction** In the last years a growing interest has been recorded towards less invasive surgical approach techniques for the anatomical structures around the hip in primary total hip arthroplasty, aiming at a reduction in the iatrogenic injury, pain, recovery and rehabilitation time. The literature review does not identify which is the best surgical approach today in terms of postoperative pain, functional recovery and peri-skeletal tissue damage. Among the classical approaches there is not a clear trend in favour of a specific one. Even if there seems to be a favourable consensus regarding the efficacy of the direct anterior approach in reducing postoperative pain and soft tissue injuries, whereas lateral and posterior approaches are the most widely employed.

**Materials and methods** A retrospective MRI study has been performed to compare transgluteal and posterior approaches in operative soft tissue injury. Modern MRI methods provides a multiplanar display with greater detail in resolving peri-skeletal structures with optimal qualities of resolution and contrast specific for different tissues. Radiological investigations involved more than 20 patients, with well functioning hip prostheses implanted by the same surgeon.

**Results** A radiologist evaluated MRI images and staged postoperative peri-articular soft tissue changes with particular attention to abductor muscles. Lateral and posterior approach groups were similar for age, gender, BMI and total hip prosthesis features.

**Discussion** Different findings in muscular injury were recorded: partial detachment and medium gluteal tendonitis, trochanteric bursitis and muscular fat replacement were significantly more frequent in lateral approach group.

**Conclusions** In the second group, accurate extrarotator tendons and posterior capsule reconstruction produced minimal morphological variations without muscular degeneration. No differences were found out in tensor fascia lata.

### A prospective randomized study about unicompartmental knee arthroplasty with all-polyethylene versus metal backed tibial component: a clinical and RSA evaluation

P. Barbadoro*, A. Ensini, A. Leardini, A. Feliciangeli, A. Timoncini, F. Cenni, S. Giannini

Istituto Ortopedico Rizzoli (Bologna, IT)

**Introduction** While excellent clinical results can be obtained with unicompartmental knee arthroplasty (UKA), higher revision rates are reported when compared to total knee arthroplasty. The tibial component aseptic loosening represents the main cause of UKA revision. The aim of this study was to evaluate: 1. if there are differences in fixation between all-polyethylene tibial component and metal-backed tibial component; 2. if the clinical scores are different in the two groups.
**Materials and methods** We recruited 40 patients undergoing medial UKA. They were randomized in 2 groups: 20 patients with an all-polyethylene tibial component (group A) and 20 patients with a metal-backed tibial component (group B), with the same femoral component for the two groups (UNI-Journey S&N). A clinical evaluation (by IKS scoring system) and a radio-stereophotogrammetric analysis (RSA) were performed at 3, 6, 12 and 24 months of follow-up. We report the results of 12 patients (6 of the group A and 6 of the group B) at 6 months of follow-up. Regarding the RSA analysis, we calculated translations and rotations of the tibial component along and about the three anatomical axes and the Maximum Total Point Motion (MTPM). Statistical analysis was performed, considering $p < 0.05$ as significant.
**Results** From the analysis of the average of the absolute values it was possible to observe that the maximum translations and rotations were respectively in medio-lateral (0.09 ± 0.06 mm) and in varus-valgus (0.5 ± 0.5°) in the group A and respectively in anterior-posterior (0.17 ± 0.19 mm) and in varus-valgus (0.7 ± 0.8°) in the group B. The mean MTPM at 6 months was 0.28 ± 0.13 mm in the group A and 0.49 ± 0.30 mm in the group B. The mean clinical score (IKS-K and -F) at 6 months was 84 ± 6 and 92 ± 13 in the group A and 89 ± 8 and 97 ± 5 in the group B.
**Discussion** In the literature, biomechanical studies describe a higher stress in the medial tibial cortex in the all polyethylene tibial component UKA respect to the metal backed ones. No differences in fixation and in clinical scores are reported at 6 months of follow-up. At 6 months of follow-up, one patient of the group B presented higher migration: posterior translation higher than 0.5 mm, internal and varus rotation both higher than 2° and a MTPM higher than 1 mm.
**Conclusions** The limited number of patients analyzed and the early follow-up do not allow to draw a conclusion on this study as yet. At this point of the study we cannot consider the all-polyethylene tibial component inferior to metal-backed one.

### Outcome of Fitmore stem in total hip arthroplasty: our experience at 24 months

E. Gasbarra*, M. Celi, F.L. Perrone, J. Baldi, N. Cuozzo, U. Tarantino

Università Tor Vergata (Rome, IT)

**Introduction** The use of femoral stems with a bone conserving related to mini-invasive surgery, able to guarantee a lesser wound of soft tissues and greater preservation of metaphyseal bone-stock than standard stems, has increased more and more in recent years. Aim of this study is to evaluate bone-remodelling, recovery of range of motion, improvement of quality of life, after 24 months in patients treated with the bone conserving Fitmore stem.
**Materials and methods** Forty bone conserving stems were studied (Fitmore-Zimmer). Mean age of patients was 60.3 years old (range 24–83). All patients were analyzed pre-operatively, with lumbar spine and femoral DEXA scans, to value bone stock quality before surgery. Bone-remodelling was estimated in early post-operative time and at 3, 6, 12 and 24 months after implant, through DEXA scans according to Gruen protocol. Functional recovery was assessed through Harris Hip Score (HHS) and Timed Up and Go (TUG) test. Quality of Life (QoL) was evaluated by SF-36 questionnaire.
**Results** The analysis of DEXA data showed BMD values of −0.56 % in ROI 1; an increasing of +4.7 % in ROI 3 and +4.2 % in ROI 7 at 24 months. Analysis of results highlighted an early functional recovery, assessed through TUG test; mean value of HHS after 24 months was 95.3. The results were better in young patients in which pre-operative lumbar spine and femoral BMD values were higher.
**Discussion** The study of bone-remodelling through DEXA scan showed not excessively marked values of metaphyseal resorption; this results can probably be due to a reduced cross-sectional area of the stem in proximal femur and a physiological re-distribution of loading forces. Mini-invasive incision, preserving gluteus muscle, allowed a rapid functional recovery and a poor blood loss.
**Conclusions** Our experience underlined that the use of Fitmore-Zimmer stem, with bone conserving concept, is surely indicated not only in young patients, with a good bone quality, but also in elderly thanks to bone-stock preserving feature. Moreover, the sparing of muscle and tendon tissues guarantees a good functional recovery already at few months after implant.

### Antero-longitudinal osteotomy of the trochanter and early dislocation prevention in hip arthroplasty through a postero-lateral approach

M.F. Surace, G.V.M. Regazzola*, L. Murena, N. Guindani, P. Cherubino

Dipartimento di Biotecnologie e Scienze della Vita, Clinica Ortopedica, Università dell'Insubria (Varese, IT)

**Introduction** Early dislocation is a foremost complication of total hip arthroplasty through a postero-lateral approach. The extraarticular impingement of the anterior part of the great trochanter with ileum bone, with or without soft tissue interposition is a well recognized but underestimated etiopathogenetical cause reported in literature. In this retrospective study through the assessment of clinical and radiographical follow-up at a minimum of 6 months, the effectiveness of an antero-longitudinal osteotomy of the great trochanter for early dislocation prevention is evaluated.
**Materials and methods** One hundred patients underwent a total hip arthroplasty from June 2011 to June 2012, with surgery being performed by the same surgeon. Sixty percent (60 %) was female and 40 % was male. A modified postero-lateral approach was used according to the tissue-sparing criteria, in all the cases an anterior longitudinal osteotomy of the great trochanter has been performed at 90° to the anteversion angle of the implant and aligned posteriorly with the prosthesis. All the patients underwent a clinical and radiological follow-up at 1, 3, and 6 months.
**Results** In this study, no dislocation was reported. One patient suffered from a wound infection which was subsequently treated with antibiotics and had complete remission. All patients demonstrated a fast recovery of ROM and walking, obtaining an Harris Hip Score of 80.15 at 3 months, and 94.7 at 6 months of follow up. After surgery and during the follow-up period, there were no trochanteric fractures detected.

**Discussion** The correct positioning of the implants, the head diameter, offset, soft tissues repair, absence of impingement, and patients compliance are all elements that define the prosthetic stability. Literature shows and incidence of primary total hip arthroplasty dislocation between 0.8 and 10 %. The incidence of dislocation reported in a preliminary study in our Institute is 1.1 %. The osteotomy of the great trochanter shows an evident decrease of incidence under 1 %.
**Conclusions** The osteotomy of the great trochanter is an effective surgical technique used to decrease the anterior impingement and early dislocation incidence. It is particularly effective on patients with good compliance and correctly implanted prosthetic components.

### *C59–PROSTHESES-HIP III*

### Analysis and comparison of peri-prosthetic BMD, hip prosthesis, between straight stem and anatomical stem

F. Bove*[1], F. Volpato[1], G. Fabiani[1], Gi. Bove[2], Ga. Bove[2]

[1]INI-Istituto Neurotraumatologico Italiano (Grottaferrata, IT)
[2]Università "La Sapienza" (Rome, IT)

**Introduction** Implanting a prosthesis causes the bone segment to adapt its macro- and microstructure to the new mechanical stresses. Two processes are then activated: bone ingrowth and bone remodelling. The prosthetic design can contribute towards the implant's fixation and stability.
**Materials and methods** Patients operated on by the same surgeon over the past year, with bilateral hip replacement with straight stem and anatomical stem coated in hydroxyapatite were assessed, with the purpose of analyzing any differences in the criteria of prosthetic fixation. They were subjected to a DEXA (dual X-ray absorptiometry) densitometric analysis using software for this purpose (metal removal) and subdividing the areas of interest in accordance with the Gruen scheme (VII zones), and to clinical/functional analysis (Harris score).
**Results** The results obtained through statistical analysis with significance by DEXA following the seven Gruen areas showed a constant increase of peri-prosthetic BMD with regard to the prosthetic design.
**Discussion** From the clinical standpoint, there are no significant differences; the prosthetic design, also with regard to the subject's physical characteristics, influences joint biomechanics and consequently peri-prosthetic BMD.
**Conclusions** The differences between the various experiences found in the literature makes interpreting the results difficult, even with regard to certain variables. The results are probably to be attributed both to the prosthetic design of both models, and to the surgical techniques, given that the criteria for a proper positioning of the prosthetic implant were at any rate respected. Analysis of a group of patients with bilateral prostheses of a different model made by the same operator reduces the aforementioned major variables, making the result more significant.

### Stems prosthetic bone preservation: why doing it, and which choices. Our experience with Fitmore stem

A. Lispi*, A. Formica, A. Campi

[1]Azienda Ospedaliera S. Giovanni (Rome, IT)

**Introduction** The Zimmer Fitmore Hip Stem is a bone-preserving system of implants that is specifically designed for use with less invasive surgical procedures. The system aims to provide stable reconstruction of each individual's anatomy through its Innovative concept of matching stem offset with different medial curvatures. The triple taper design with proximal Ti-Plasma coating creates a press fit and proximal transfer of forces, which Is supported by the apposition on the cancer region. Primary fixation and rotational stability is ensured by the trapezoidal cross-section of the stem, apposition on the calcar region, and the lateral cortex in the subtrochanteric region. The Fitmore system is comprised of three different families, with Family B available in an extended offset option. The families were developed to address the relationship between head centre and medial curvature of the femoral canal. Each stem family differs in respect to its medial contour, neck shaft angle, and range of stem offsets.
**Materials and methods** Prospective study from January 2010 to December 2012. All patients undergoing first implantation of hip replacement were included. The very few exclusion criteria were essentially the contraindication to implant of a femoral stem not cemented, or the necessity of a tapered stem. All stems were implanted coupled with cementless cups, different construction characteristics and tribology, depending on age, functional requirements of the patient and the need for fixation. The clinical evaluation was performed by calculating the Harris Hip Score and recording adverse events.
**Results** The clinical and radiographic results of the prosthesis Fitmore are satisfactory. At a mean follow-up of 12 months over 98 % of patients were reported to be painless. At last follow-up, the average Harris Hip Score improved significantly from 58.1 to 95.1.
**Discussion** The rationale of saving trochanteric is the strong point of this rod because, in spite of the improvement of tribology, the functional degradation of the stems upright standards depends largely on the trochanteric osteolysis and related complications.
**Conclusions** Our experience with the stem Fitmore is certainly positive, with clinical and radiographic results in the short term comparable or better than those previously observed for standard uncemented stems. Spreader is a reconstruction of the appropriate morphotype and morphometric of the pre-operative in a very wide range of patients without a noticeable increase in complications.

### Severe retroperitoneal migration of THA

A. Belluati*, C. Goretti, E. Mariani, G. Maldini, A. Colombelli

Santa Maria delle Croci (Ravenna, IT)

**Introduction** The acetabular cup prosthesis has an intrapelvic migration when it exceeds the ileo-pectineal line and penetrates inside of the small pelvis, coming in contact with the abdominal organs. It's an unusual complication which poses the problem not only of reconstruction, but especially removal of the cup inside of the pelvis. A traditional approach to the hip raises the risk of serious complications particularly with vessels, could cause bleeding sometimes uncontrollable and deadly. In these cases it is preferable an abdominal way.
**Materials and methods** From April 2008 to date we have treated 4 patients with THA severe intrapelvic migration. All patients underwent to a dual surgical procedure performed in the same operative session: a first time performing ad anterior abdominal way for retroperitoneal removal of the cup and a second with postero-lateral access to hip joint for reconstruction of THA. The first time was performed always by a vascular surgeon. For the reconstruction we used a revision cup in porous surface with fins that allowed early mobilization and the load to 40 days from surgery.
**Conclusions** The retroperitoneal way has proven to be the ideal procedure in case of intrapelvic migration of a THA. It allows you to remove the adhesions between the prosthesis and the intrapelvic structures, isolate and mobilize the vessels, blocking the bleeding in case of laceration of a vessel allowing the repair. In this way it is possible to extract safely through the dome or the same retroperitoneal or through a standard approach to hip. An adequate preoperative planning and a multidisciplinary surgical team are essentials. The THA revision can be performed during the same operative session as long as the patient is stable from a hemodynamic point of view and has been ruled out the presence of infections.

### *C60–PROSTHESES-MISCELLANEOUS*

### Unicompartmental knee arthroplasty provides early improvements

M. Vasso*, S. Cerciello, A.M. Felici, K. Corona, A. Schiavone Panni

Università del Molise (Campobasso, IT)

**Introduction** Improvement of surgical technique, materials and prosthetic design made the unicompartmental knee replacement an efficient and reliable procedure, with excellent or good mid- to long-term results in most patients. The average 10 year survival rate for unicompartmental knee arthroplasties, in selected patients, is approximately 90 %, comparable to that of a primary total knee replacement. Unicompartmental knee replacement preserves uninvolved osteo-cartilagineous and soft tissue structures, thereby allowing a more physiological and early clinical and functional recovery. The aim of this study was to report the results of ZUK unicompartmental fixed metal-back prosthesis, and how these results change over time.
**Materials and methods** Eighty ZUK prostheses were implanted in 80 patients for unicompartmental osteoarthritis or osteonecrosis. Patients were clinically assessed using the International Knee Society Scores. Post-operative values of mechanical axis were calculated 12 months after surgery and compared to the pre-operative ones.
**Results** The mean International Knee Society knee and function scores improved respectively from $46 \pm 9$ and $54 \pm 8$ pre-operatively to $82 \pm 5$ and $94 \pm 3$ at the last follow-up ($p < 0.001$). Average flexion increased from $110° \pm 9°$ to $127° \pm 8°$ ($p < 0.01$). Patients with unicompartmental knee reached good clinical outcome very early and remained at the same level. Age didn't significantly influence clinical and functional scores.
**Discussion** This study confirmed that patients with an unicompartmental knee reach good clinical and functional scores and the maximum range of movement very early. IKS knee scores and knee flexion reached the highest values very early, respectively at 6 months and at 3 months, then levelled out over time. IKS functional scores stabilized 1 year after operation, but patients referred to obtain complete functional recovery earlier, generally 8–9 months after surgery. Moreover, there weren't any significant differences in terms of IKS scores and knee flexion at the last follow-up between the patients over 60 and those younger.
**Conclusions** High success rates of the modern unicompartmental knee implants depend on the materials and design evolution, improvement of the surgical technique, and on the strong restriction of indications. Unicompartmental knee arthroplasties can provide good clinical and functional outcomes both in patients over 60, than in younger more demanding patients.

### Unicompartmental knee revision

M. Massaro*, F. Verde, M. Corbella, S. Romagnoli

Istituto Ortopedico Galeazzi (Milan, IT)

**Introduction** Revision can be performed differently depending on the cause of failure and the elapsed time since the first implant. Considering the kind of prosthesis implanted during revision, the failure causes and the prosthesis survivor rate, we can observe that it represents a standard procedure with good results. Among the group of failure cases, a comparison between the groups of UNI revisions performed with the use of another UNI or a TKR was made and no significative differences were highlighted.
**Materials and methods** From February 2001 to December 2011, we performed 220 unicompartmental revisions in 213 patients. The clinical examination included the evaluation of the skin incision, the condition of the soft tissues, the range of motion, the ligamentous stability, the integrity of the extensor mechanism and the neurovascular structures. Radiographic analysis evaluated the mechanical axis, the position of the components on the frontal and sagittal planes, the signs of loosening of the components, the lack of bone stock and the level of the joint line.
**Results** The average follow-up of the 220 patients was 7.4 years. In 138 cases (62.7%) we performed a TKA and in 82 cases (37.3%) another unicompartmental implant. In 19 (13.8%) cases we used a semi-constrained prosthesis. In 13 cases the review was carried out in two steps due to septic loosening. Instead, in 82 cases we reviewed the unicompartmental with another UNI.
**Discussion** The results depend on the correct indications. In some cases, UNI to UNI revision was performed or, when all the conditions were respected, an implant of another unicompartmental prosthesis in the contralateral compartment or the implantation of a patello-femoral prosthesis (resulting in a bi-unicompartmental implant) were performed. The advantages of these solutions are based on the mini invasive surgical procedure, and consequently on the rapid functional recovery. Conversely, when it is indicated, a total knee prosthesis is carried out and the results depend on the characteristics of the patient, the quality of the soft tissues and the bone defect.
**Conclusions** This work reports no significative difference in survival rate between TKR and UNI revisions, with rather little advantage of these latter ones. These results can be explained with a simple surgical rule: with the same appropriate indication and surgical technique there is a lower risk of complications in less invasive surgeries.

### The register for shoulder implant replacements in Puglia Region: analysis and perspectives

G. Gravina*[1], S. Mudoni[1], V. Siciliani[1], M.T. Balducci[1], C. Germinario[1], A. Aloisi[2], G. Bernardini[3], U. Biasi[4], A. Bozzi[5], L. Brina[6], M. Capozzi[7], V. Fantetti[8], V.N. Galante[9], P. Galluccio[10], R. Laforgia[11], D. Laghezza[12], A. Latela[13], A. Leo[14], G. Lo Bianco[15], S. Lotito[16], A. Macchiarola[17], W. Mega[18], A. Mocci[19], C. Monteleone[20], B. Moretti[21], T. Nevoli[22], D. Panetta[23], L. Petrelli[24], R. Recchia[25], L. Refolo[26], A. Ricciardiello[27], G. Rollo[28], M. Scialpi[29], F. Specchiulli[17], V. Tempesta[30], E. Vigilante[31], A. Villani[32]

[1]Osservatorio Epidemiologico della Regione Puglia (Bari, IT);
[2]P.O. "S. Caterina Novella" (Galatina, IT);
[3]Casa di Cura Bernardini (Taranto, IT);
[4]P.O. "Vito Fazzi" (Lecce, IT);
[5]P.O. "A. Perrino" (Brindisi, IT);
[6]P.O. "San Paolo" (Bari, IT);
[7]Casa di Cura S. Maria (Bari, IT);
[8]P.O. "T. Masselli Mascia" (San Severo, IT);
[9]P.O. Occidentale (Castellaneta, IT);
[10]Casa di Cura Villa Bianca (Lecce, IT);
[11]Casa di Cura Santa Rita (Bari, IT);
[12]P.O. (Bisceglie-Trani, IT);

[13]P.O. “San Giacomo” (Monopoli, IT);
[14]P.O. (Copertino-Nardò, IT);
[15]Casa Di Cura Villa Lucia (Conversano, IT);
[16]P.O. “L. Bonomo” (Andria, IT);
[17]A.O.U. Ospedali Riuniti (Foggia, IT);
[18]P.O. “Sacro Cuore di Gesù” (Gallipoli, IT);
[19]Casa di Cura Anthea (Bari, IT);
[20]P.O. Valle d’Itria (Martina Franca, IT);
[21]A.O.U. Policlinico (Bari, IT);
[22]P.O. “San Marco” (Grottaglie, IT);
[23]Casa di Cura F. D’Amore (Taranto, IT);
[24]P.O. “Umberto I” (Altamura, IT);
[25]P.O. “S. Maria degli Angeli” (Putignano, IT);
[26]P.O. “Veris delli Ponti” (Scorrano, IT);
[27]Casa Bianca Hospital (Cassano, IT);
[28]E.E. Miulli (Acquaviva, IT);
[29]P.O. Orientale (Manduria, IT);
[30]E.E. “Cardinale G. Panico” (Triggiano, IT);
[31]IRCCS Casa Sollievo della Sofferenza (San Giovanni Rotondo, IT);
[32]P.O. “Lastaria” (Lucera, IT)

**Introduction** The Regional Law No. 4 of 25 February 2010 marks the beginning of a Register for Shoulder Implant Replacement (RIPO) with the aim of creating a regional database, which is essential to ensure the traceability of implants and having the endpoint detection as the failure of implant. The target of this paper is to describe the results of the first 2 years of the RIPO.

**Materials and methods** From 1 January 2010 to 31 December 2011, both public and private Divisions of Orthopaedics and Traumatology posted 384 survey forms to the Epidemiological Observatory of the Puglia Region (OER), the regional coordination centre. The survey form provides a variety of information: personal, clinical and surgical data, medical devices implanted and possible complications. It is filled by the ward doctor when the patient is discharge, sent to OER, where it is entered in a database created with software FileMakerPro. A record linkage with the hospital discharge forms (SDO) is performed. Epi-Info software 6.00 is used for data analysis.

**Results** The record linkage with the SDO showed a level of completeness of 85 % in 2010 and 76 % in 2011. The comparison of the rates of hospitalization for primary implant in Puglia and Italy in the period 2001–2010 showed a steady growth, with an average annual increase of 12 %. The records were from all public and private accredited health facilities (36.9 vs 63.1 %). In 2011, 61.2 % (54.1 % in 2010) of the patients were subjected to a total joint surgery, 36.3 % (43.7 % in 2010) to a partial arthroplasty and 2.5 % (2.2 % in 2010) for a revision. The median age was 72 ± 8 years. Main cause for the surgery was the fracture for the first implant and the mobilization of the humeral component for the replanting. In 40.7 % the stems used were anatomical, of these 54.6 % modular and the remaining monoblock.

**Discussion** The analysis of the data showed that, in 2001–2010, the time trend in hospitalization rates for primary implant in Puglia is constantly increasing and comparable to the national average. The compliance of the register is satisfactory.

**Conclusions** Our short-term goal is to achieve the completeness of RIPO close to 100 %. A valid database is essential to establish long-term results of orthopaedic treatment and to allow adequate traceability of implanted devices.

### Clinical experience with Dabigatran in prosthetic hip and knee surgery

M. Scardino*, G. Mazziotta, F. Traverso, F. Astore

Istituto Clinico Humanitas (Milan, IT)

**Introduction** This protocol was developed to overcome the drawbacks places for the implementation of regional anaesthesia and management of epidural catheters or perinervous to solve the problems of recruitment in the immediate post-operative patients, particularly in advanced age or in the presence of nausea and/or vomiting and control in the immediate post-operative blood loss.

**Materials and methods** In the evening of surgery and for the next 2 days, however, until the presence of perinervous catheters, drains and postoperative epidural anaesthesia, patients will be administered by subcutaneous doses of LMWH and prescription based on the molecule choice. The morning after the last administration of 220 mg of Dabigatran LMWH unless otherwise specified folder according to the clinical condition of the patient. Patients involved in this protocol must be followed for the entire stay in the hospital until discharge and control the following 28 days after discharge, indicating in each case up to the suspension of antithrombotic therapy, using the control card prepared. The data collected and analyzed for: Hb, Ht, Plt, Pt, Ptt, complications, effectiveness, adherence to prescription possible reasons for the interruption. Involved 356 patients in 6 months.

**Results** Adherence to therapy in all cases, with no adverse effects, no complication related, Hb, Ht, RC in the normal range, no cases of deep vein thrombosis or major thrombotic complications.

**Discussion** The protocol described thus proved efficacy did not alter either the medical management of the nursing department. There was no problem with the administration of absolute Dabigatran in patient management.

**Conclusions** We believe that this protocol is the best way to make it easier and safer to use this new oral anticoagulant.

### Proximal humeral fractures treatment in elderly using a reverse prosthesis

C. Iacobellis*, A. Camporese, R. Aldegheri

Clinica Ortopedica, Università di Padova (Padua, IT)

**Introduction** Complex proximal humeral fractures in the elderly are difficult, due to problems of friable bone which obstructs proper insertion of screws and plates and which often does not allow early mobilization of the shoulder, resulting in serious stiffness. Solutions were studied on a sample of patients over 64 years.

**Materials and methods** This study examines 39 patients (5 men, 34 women), mean age 77 (range 64–85 years), with humeral fractures with 4 fragments, fractures of the anatomic neck with dislocation of the head, and dislocated fractures of the head itself. The deltoid-pectoral pathway was used, with re-insertion of tuberosities. After surgery, a brace allowing 15° of abduction was applied for 4 weeks. Patients were re-examined according to Constant’s score, DASH score and X-ray examination. The percentage ratio between operated and opposite shoulders was calculated at each check-up.

**Results** Mean follow-up was 4 years. Total Constant score was 65 (pain 13/15, daily activities 16/20, power 4/25, ROM 28/40). Mean joint movements were: forward 120°, abduction 100°, extrarotation 8/10, intrarotation 6/10. DASH score 39. Total Constant score for opposite shoulder was 76 (pain 15/15, daily activities 20/20, strength 7/25, ROM 34/40). Mean movements of opposite shoulder: forward 150°, abduction 130°, extrarotation 10/10, intrarotation 8/10. There were 5 complications during surgery (4 diaphyseal fractures; 1 lesion of the subclavian artery adherent to an old fracture) and 2 immediately after surgery (prosthesis dislocation), all resolved. Long-term complications (mean 5 years) were 4 cases of scapular notching which, in 2 cases, required re-operation.

**Discussion** The deltoid-pectoral pathway was the most suitable, especially with meta-diaphyseal fractures which were not evident in pre-surgery X-rays. Re-insertion of tuberosities should always be carried out in cases with partial damage to the rotator cuff, in order to increase the range of rotations. The glenosphere should be tilted at $<10°$, to avoid notching, which occurred in the first cases operated without a tilt.

**Conclusions** Reverse prosthesis for patients with complex humeral fractures offers good results, and follow-ups show that prostheses now have increasingly longer duration. The best results are seen in younger patients. Resolution of pain is always satisfactory. However, in the elderly, the problem of good functional recovery can only be solved if patients are carefully selected. The degree of collaboration is important for effective and prolonged physiokinesitherapy.
